# Supplementary material for: Evolutionary relaxation and functional change of INSL3 and RXFP2 may underlie natural cryptorchidism in mammals
Source: EMBO Rep. 2025 Nov 11;26(24):6418–36. doi: 10.1038/s44319-025-00636-w (PMC12714730; doi:10.1038/s44319-025-00636-w)
Supplement: Supplementary file 1 — Table EV1 [file 44319_2025_636_MOESM1_ESM.docx]

**Table EV1 Evolutionary rates of INSL3 and RXFP2 in mammalian clades**

| Gene | Foreground branch | lnL (one ratio) | lnL (two ratio) | ω in background branch | ω in foreground branch | P value | FDR |
| --- | --- | --- | --- | --- | --- | --- | --- |
| *INSL3* | Cryptorchid mammals | -3768.995064 | -3761.088812 | 0.17555 | 0.37219 | 6.99E-05 | 0.000629 |
|  | Scrotal mammals | -3768.995064 | -3765.07672 | 0.26746 | 0.15835 | 0.00512 | 0.011519 |
|  | Cetaceans | -3768.995064 | -3766.885279 | 0.21679 | 1.02108 | 0.03996 | 0.05994 |
|  | Hippopotamus | -3768.995064 | -3765.599296 | 0.21507 | 1.40146 | 0.009159 | 0.016486 |
|  | Afrotherians | -3768.995064 | -3764.936929 | 0.20779 | 0.56916 | 0.004387 | 0.011519 |
|  | Pinnipeds | -3768.995064 | -3768.373512 | 0.2228 | 0.07123 | 0.264874 | 0.264874 |
|  | Cryptorchid rodents | -3768.995064 | -3764.450898 | 0.20399 | 0.51677 | 0.002572 | 0.011519 |
|  | Flying foxes | -3768.995064 | -3768.108972 | 0.22016 | 0.246591 | 0.183112 | 0.23543 |
|  | Eulipotyphlans | -3768.995064 | -3768.365505 | 0.22852 | 0.16644 | 0.261818 | 0.264874 |
| *RXFP2* | Cryptorchid mammals | -22928.9352 | -22918.61941 | 0.20889 | 0.29051 | 5.57E-06 | 4.69E-05 |
|  | Scrotal mammals | -22928.9352 | -22927.87189 | 0.23994 | 0.21526 | 0.144759 | 0.325708 |
|  | Cetaceans | -22928.9352 | -22919.21962 | 0.22696 | 0.60546 | 1.04E-05 | 4.69E-05 |
|  | Hippopotamus | -22928.9352 | -22928.23162 | 0.23097 | 0.32961 | 0.235528 | 0.423951 |
|  | Afrotherians | -22928.9352 | -22922.23467 | 0.22214 | 0.33096 | 0.000251 | 0.000754 |
|  | Pinnipeds | -22928.9352 | -22928.87677 | 0.23216 | 0.18397 | 0.732452 | 0.824009 |
|  | Cryptorchid rodents | -22928.9352 | -22928.84442 | 0.23117 | 0.24557 | 0.670033 | 0.824009 |
|  | Flying foxes | -22928.9352 | -22928.69872 | 0.23221 | 0.1353 | 0.491628 | 0.737441 |
|  | Eulipotyphlans | -22928.9352 | -22928.9352 | 0.23199 | 0.23191 | 0.997743 | 0.997743 |
