# Supplementary material for: Evolutionary relaxation and functional change of INSL3 and RXFP2 may underlie natural cryptorchidism in mammals
Source: EMBO Rep. 2025 Nov 11;26(24):6418–36. doi: 10.1038/s44319-025-00636-w (PMC12714730; doi:10.1038/s44319-025-00636-w)
Supplement: Supplementary file 2 — Appendix [file 44319_2025_636_MOESM2_ESM.pdf]

Table of Content

Appendix Figure S1......1
Appendix Figure S2......2
Appendix Table S1 3

Appendix Figure S1

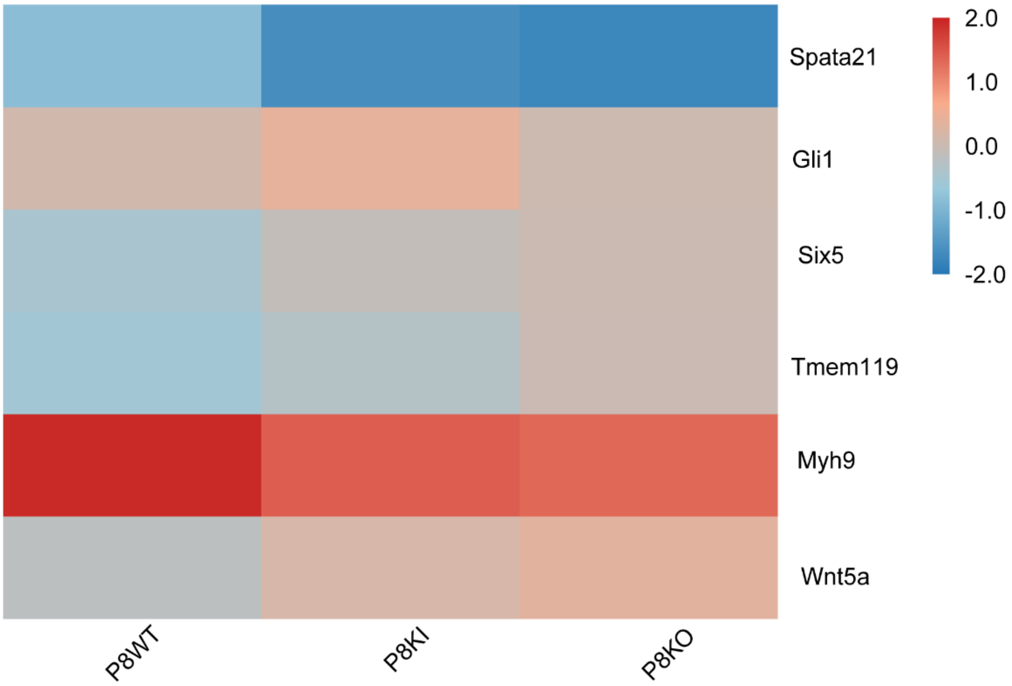

Appendix Figure S1 Heat map of the expression of meiosis and spermatogenesis related genes in the testes of mice at P8

Appendix Figure S2

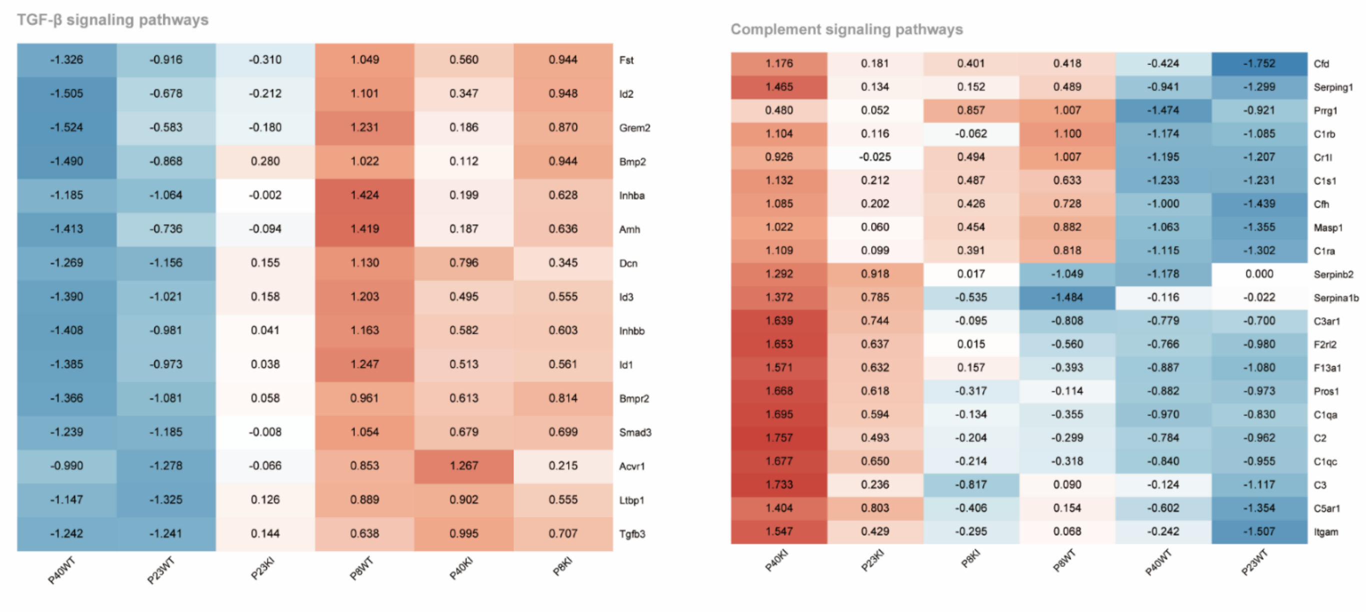

Appendix Figure S2 Heatmaps showing gene expression changes in the complement and TGF-β signaling pathways.

Appendix Table S1

Appendix Table S1 Primers used in this study to identify the genotype of transgenic mice.

Primer	Sequence
**F-INSL3**	5'- TTCAAGGTCCCAAGCTGGACA -3'
**R-INSL3**	5'- GACTCCAGTCTTTGCTATCAGGAGG -3'
**F-RXFP2**	5'- CCTTACCCTTCTTACACCGTGATGA -3'
**R-RXFP2**	5'- GGGATGTCTATCTTCCAAACACCG -3'
**F-musINSL3**	5'- CTGCTACTGATGCTCCTGGC -3'
**R-musINSL3**	5'- CAGACCCAACAGGTCTTGCT -3'
**F-balINSL3**	5'- GAAGCGCCAGAGAAGCTGT -3'
**R-balINSL3**	5'- CGAGTACCAGCATGGGGTC -3'
