# Supplementary figures and images for: Evolutionary relaxation and functional change of INSL3 and RXFP2 may underlie natural cryptorchidism in mammals
Source: EMBO Rep. 2025 Nov 11;26(24):6418–36. doi: 10.1038/s44319-025-00636-w (PMC12714730; doi:10.1038/s44319-025-00636-w)

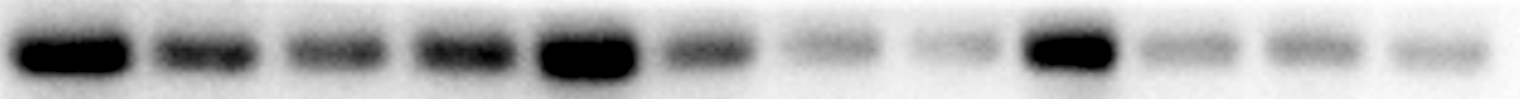

Supplement: Supplementary file 4 — Source data Fig. 1 [file 44319_2025_636_MOESM4_ESM.zip › Figure1/1E/creb1.jpg]

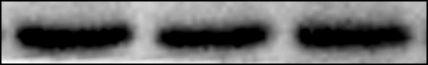

Supplement: Supplementary file 4 — Source data Fig. 1 [file 44319_2025_636_MOESM4_ESM.zip › Figure1/1E/tubulin7-1-.jpg]

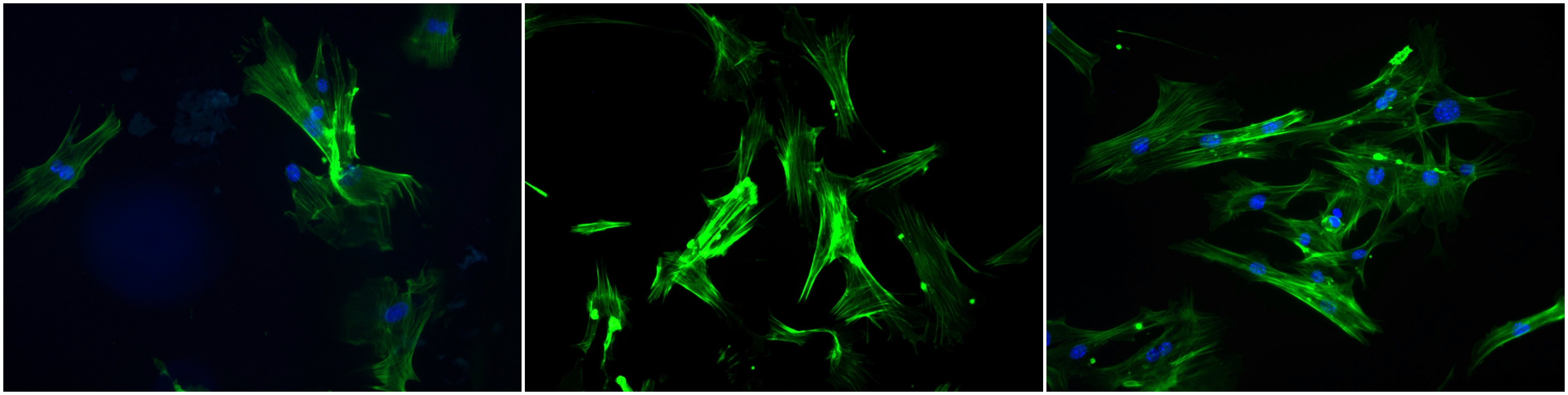

Supplement: Supplementary file 5 — Source data Fig. 2 [file 44319_2025_636_MOESM5_ESM.zip › Figure2/2B/24h.jpg]

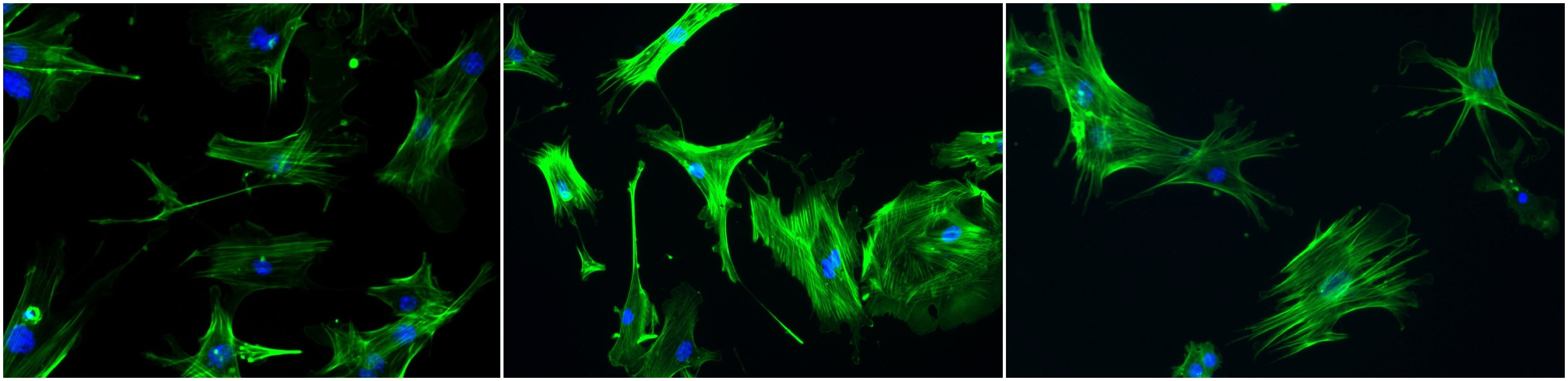

Supplement: Supplementary file 5 — Source data Fig. 2 [file 44319_2025_636_MOESM5_ESM.zip › Figure2/2B/48h.jpg]

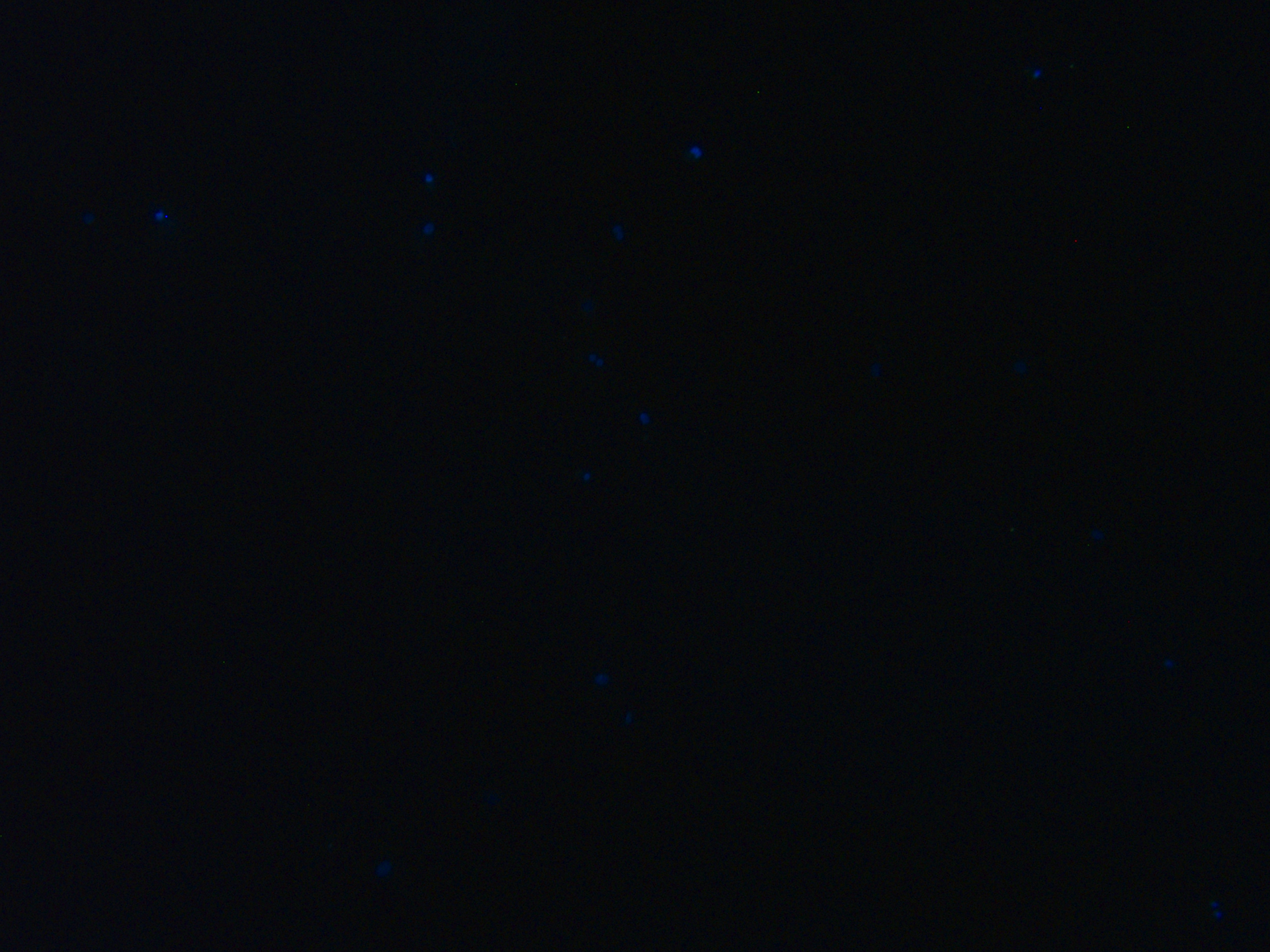

Supplement: Supplementary file 5 — Source data Fig. 2 [file 44319_2025_636_MOESM5_ESM.zip › Figure2/2B/image0001.tif]

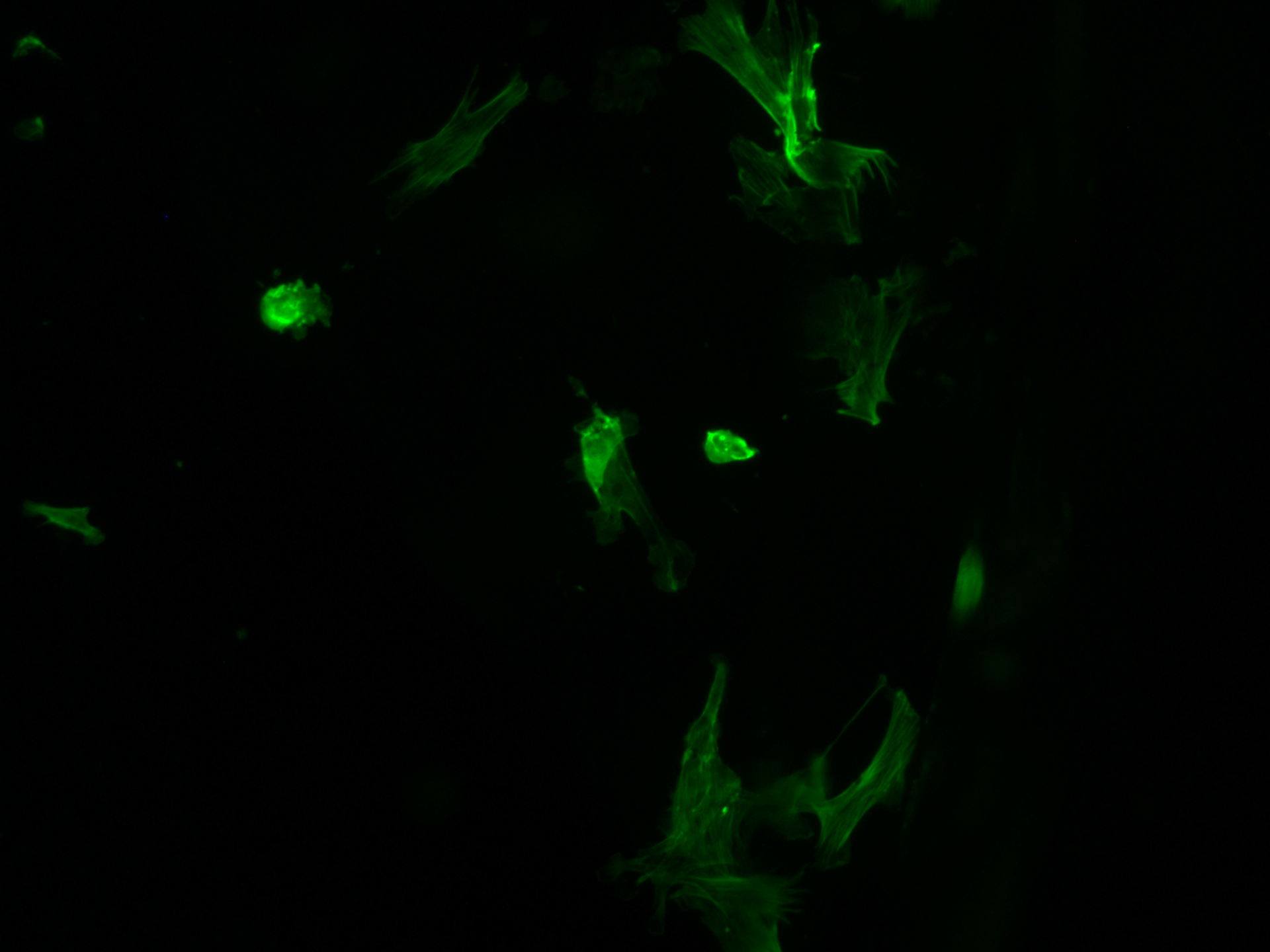

Supplement: Supplementary file 5 — Source data Fig. 2 [file 44319_2025_636_MOESM5_ESM.zip › Figure2/2B/image0002鼠1.tif]

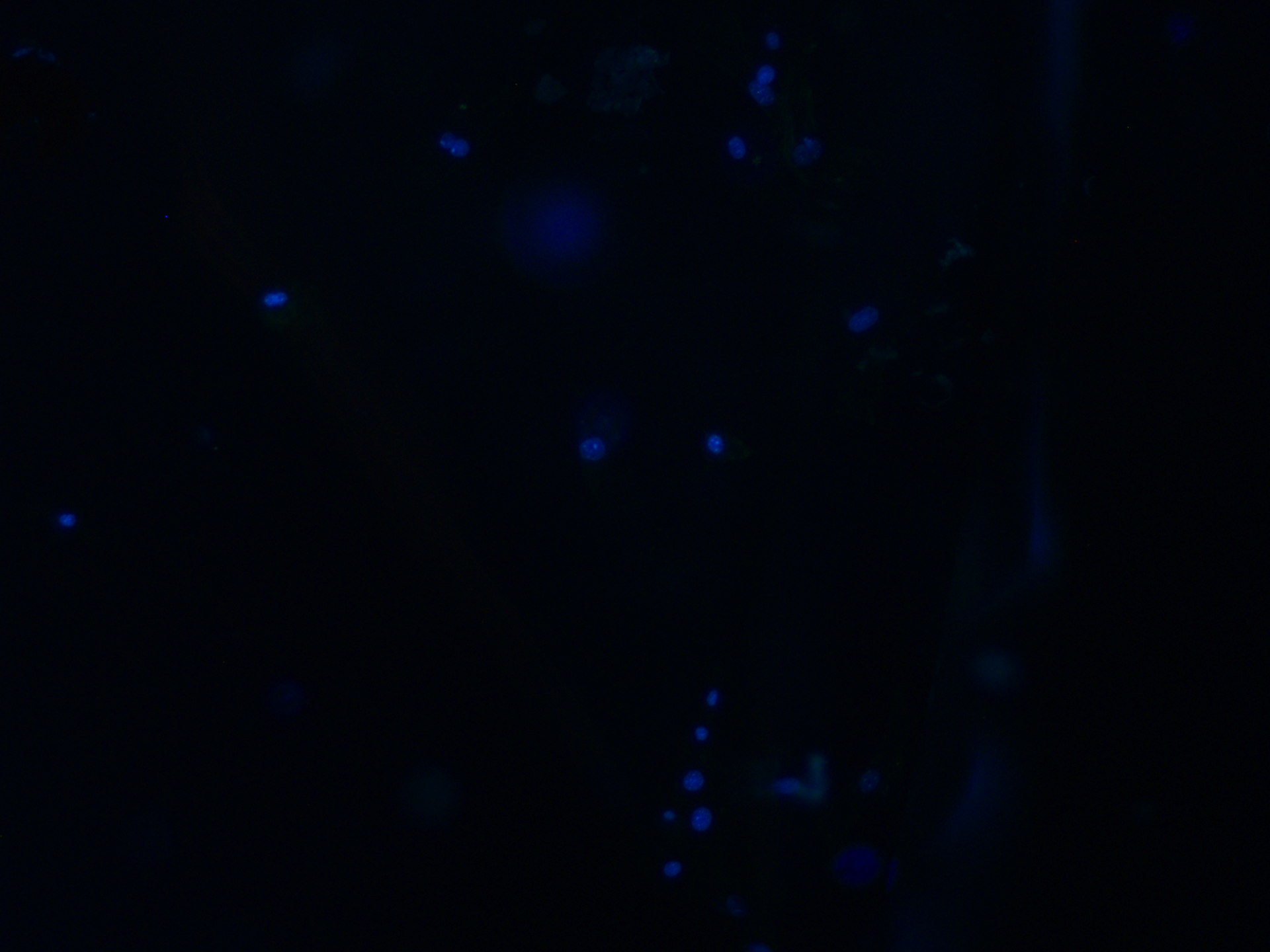

Supplement: Supplementary file 5 — Source data Fig. 2 [file 44319_2025_636_MOESM5_ESM.zip › Figure2/2B/image0003.tif]

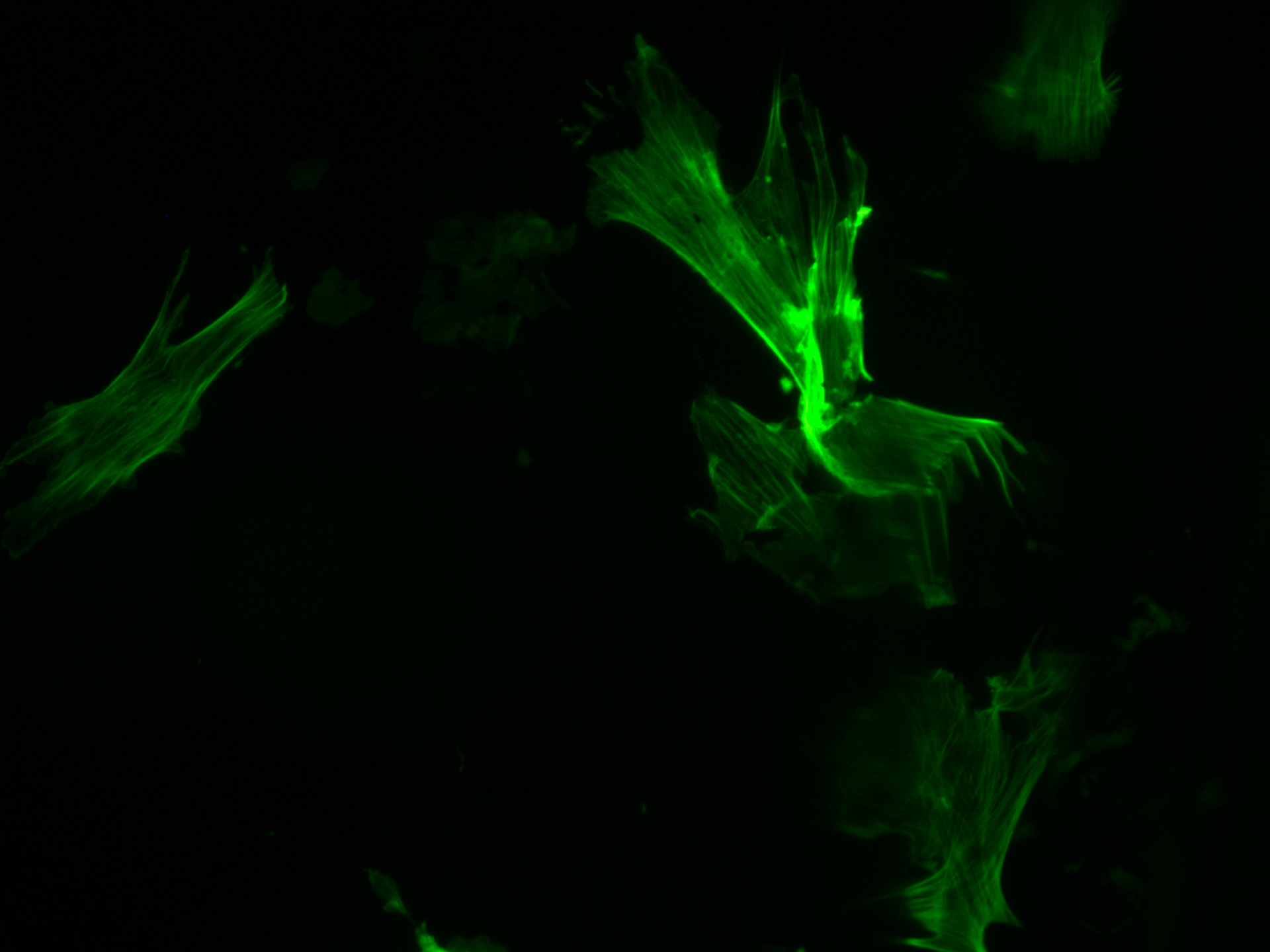

Supplement: Supplementary file 5 — Source data Fig. 2 [file 44319_2025_636_MOESM5_ESM.zip › Figure2/2B/image0004对照.tif]

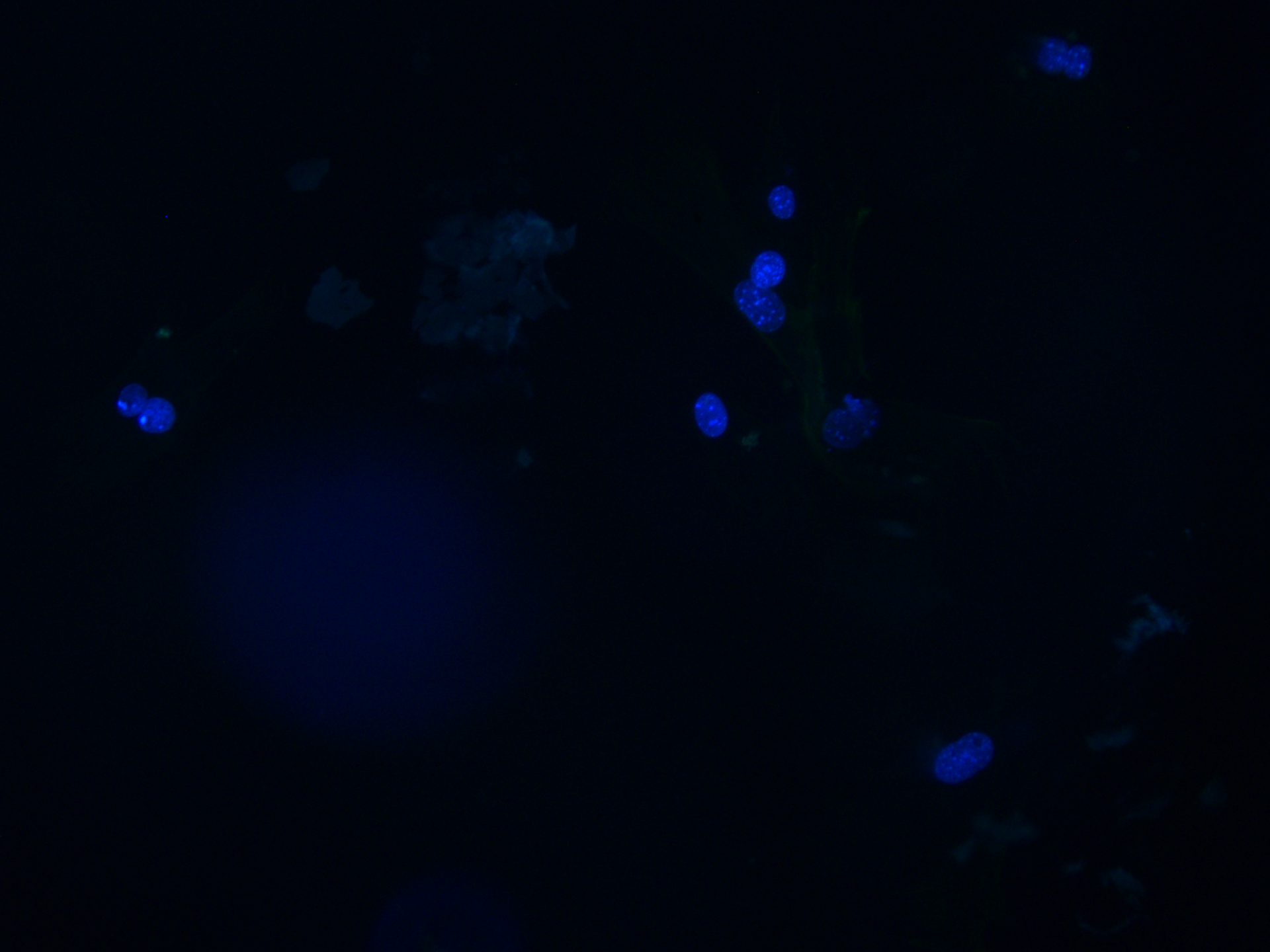

Supplement: Supplementary file 5 — Source data Fig. 2 [file 44319_2025_636_MOESM5_ESM.zip › Figure2/2B/image0005.tif]

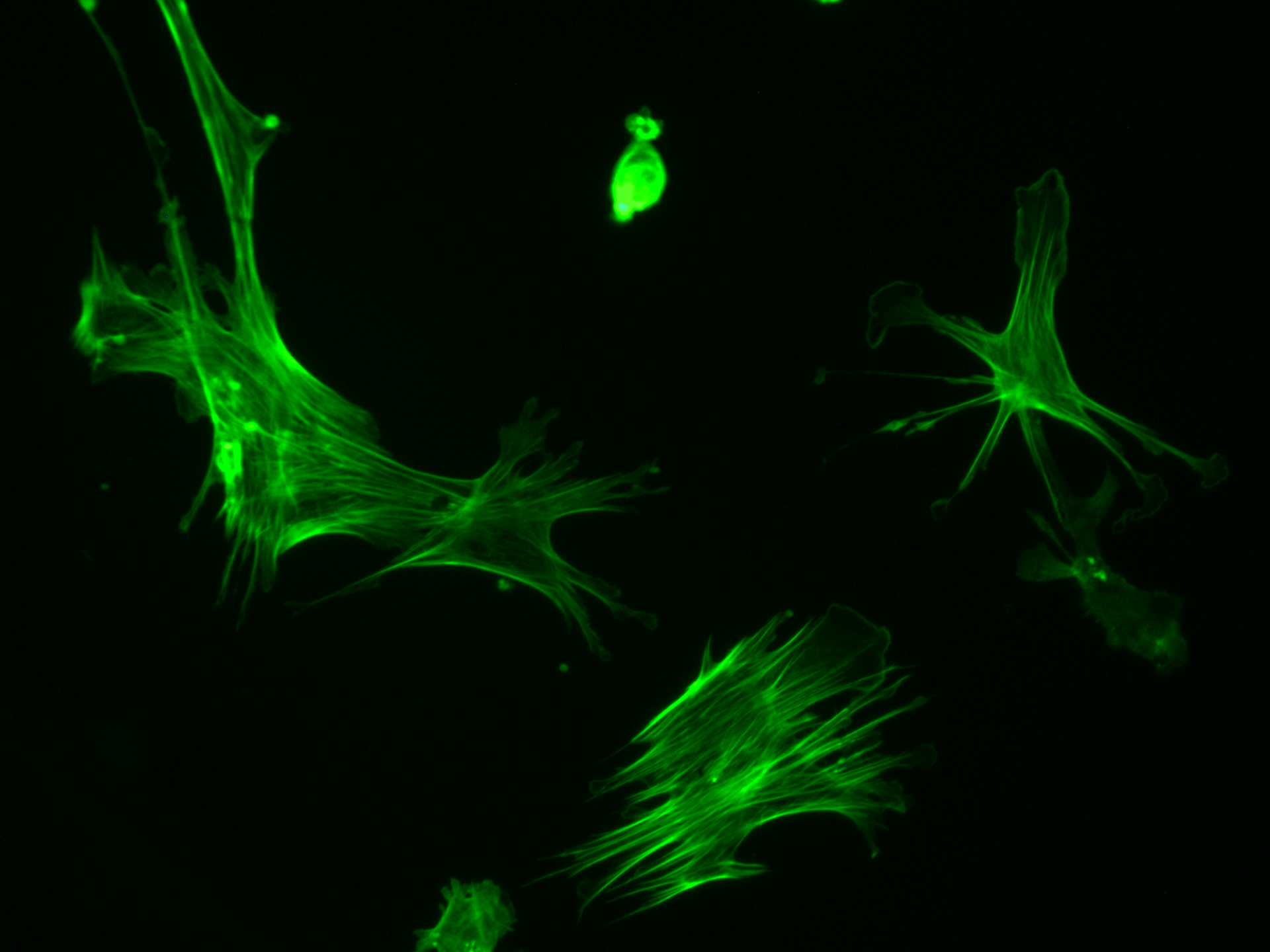

Supplement: Supplementary file 5 — Source data Fig. 2 [file 44319_2025_636_MOESM5_ESM.zip › Figure2/2B/image0006鲸.tif]

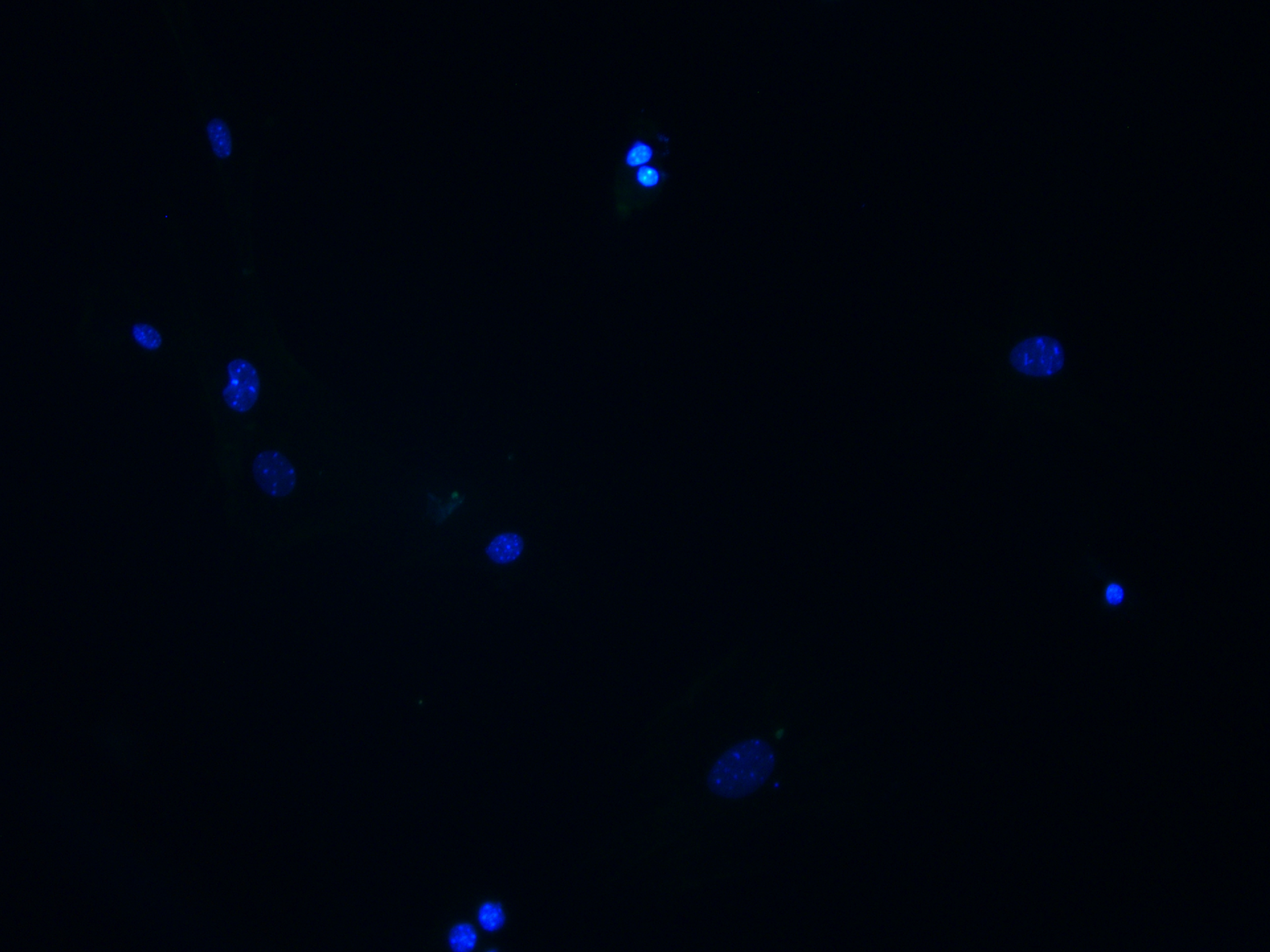

Supplement: Supplementary file 5 — Source data Fig. 2 [file 44319_2025_636_MOESM5_ESM.zip › Figure2/2B/image0007.tif]

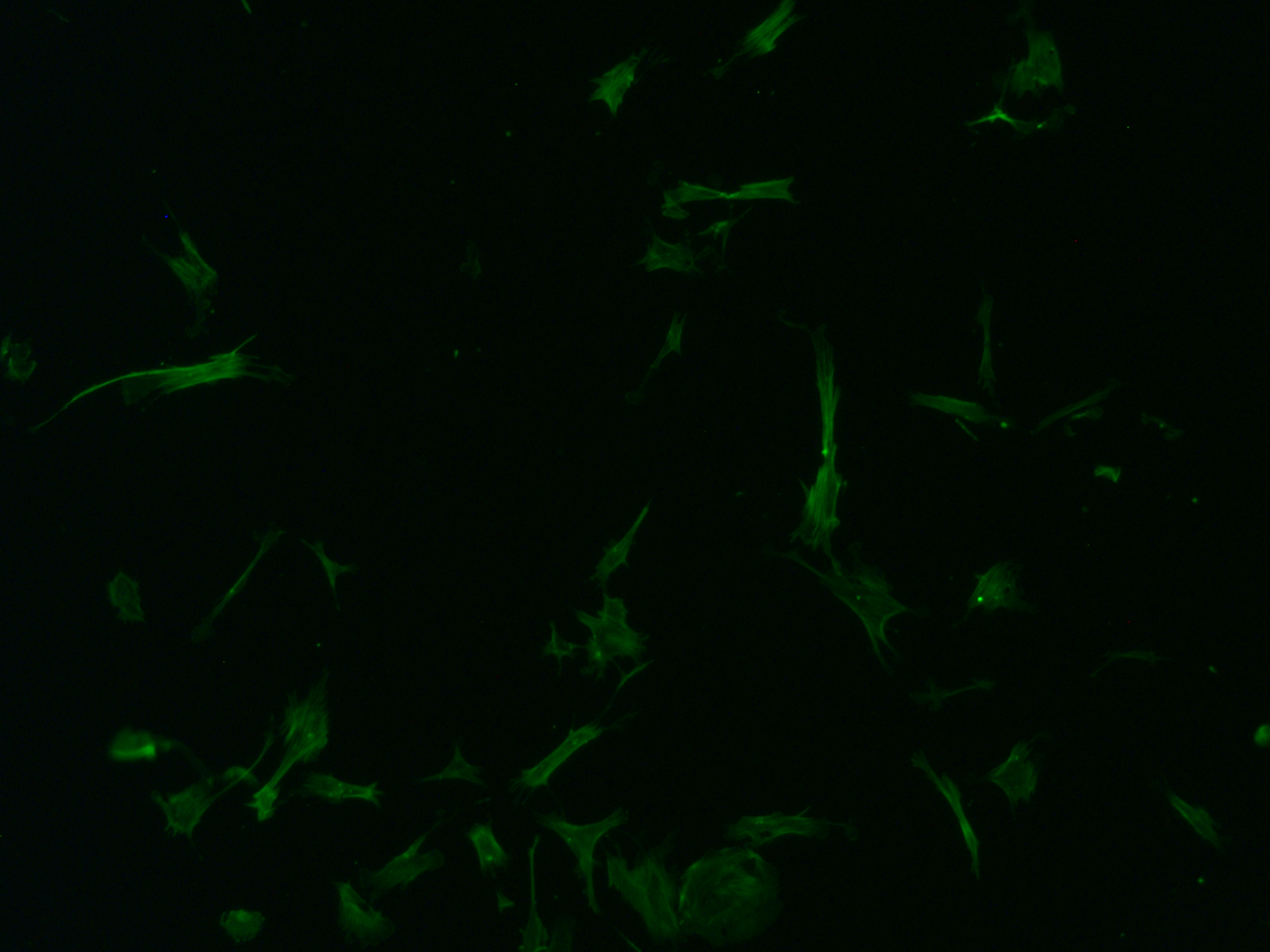

Supplement: Supplementary file 5 — Source data Fig. 2 [file 44319_2025_636_MOESM5_ESM.zip › Figure2/2B/image0008鲸1.tif]

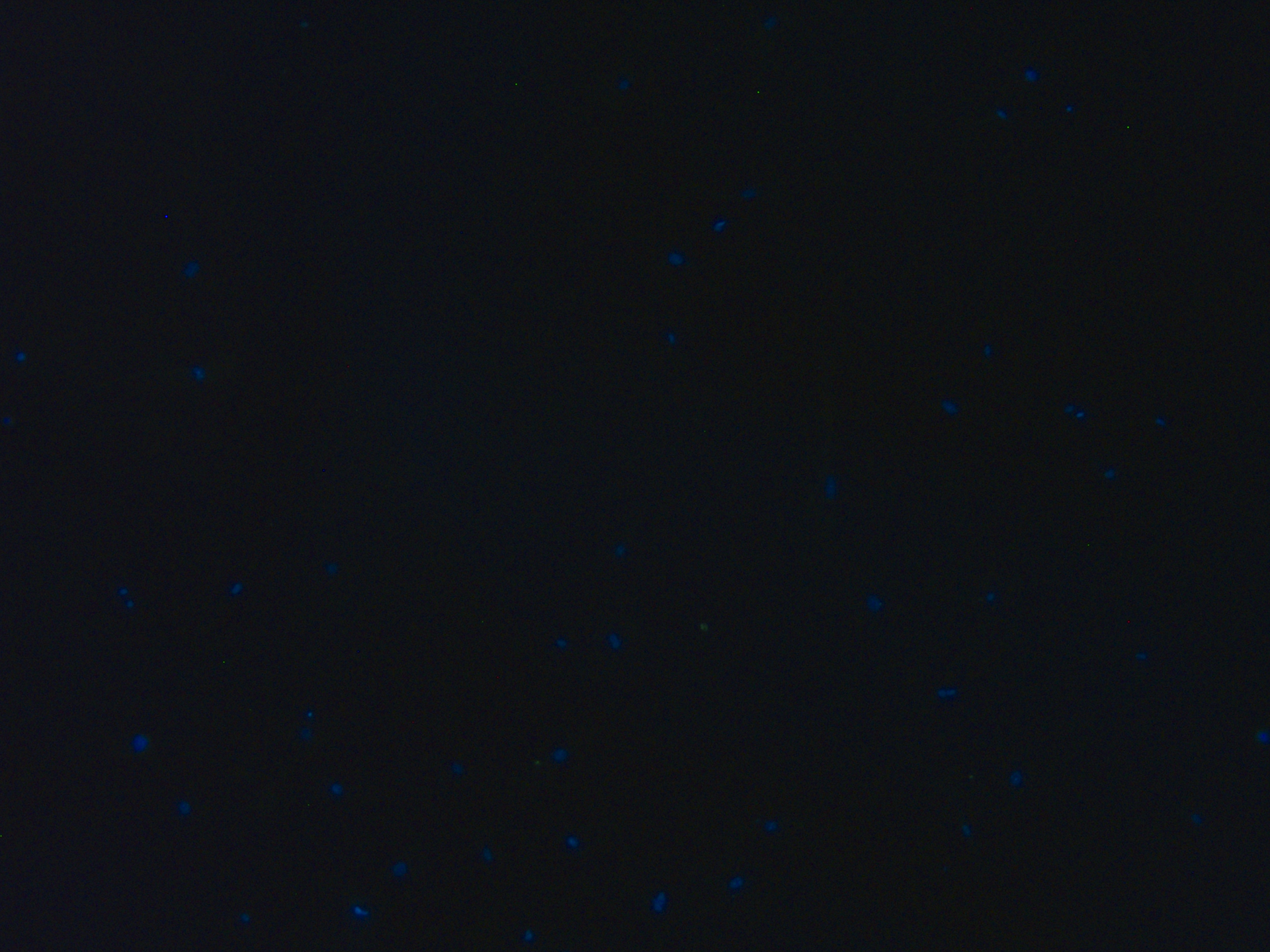

Supplement: Supplementary file 5 — Source data Fig. 2 [file 44319_2025_636_MOESM5_ESM.zip › Figure2/2B/image0009.tif]

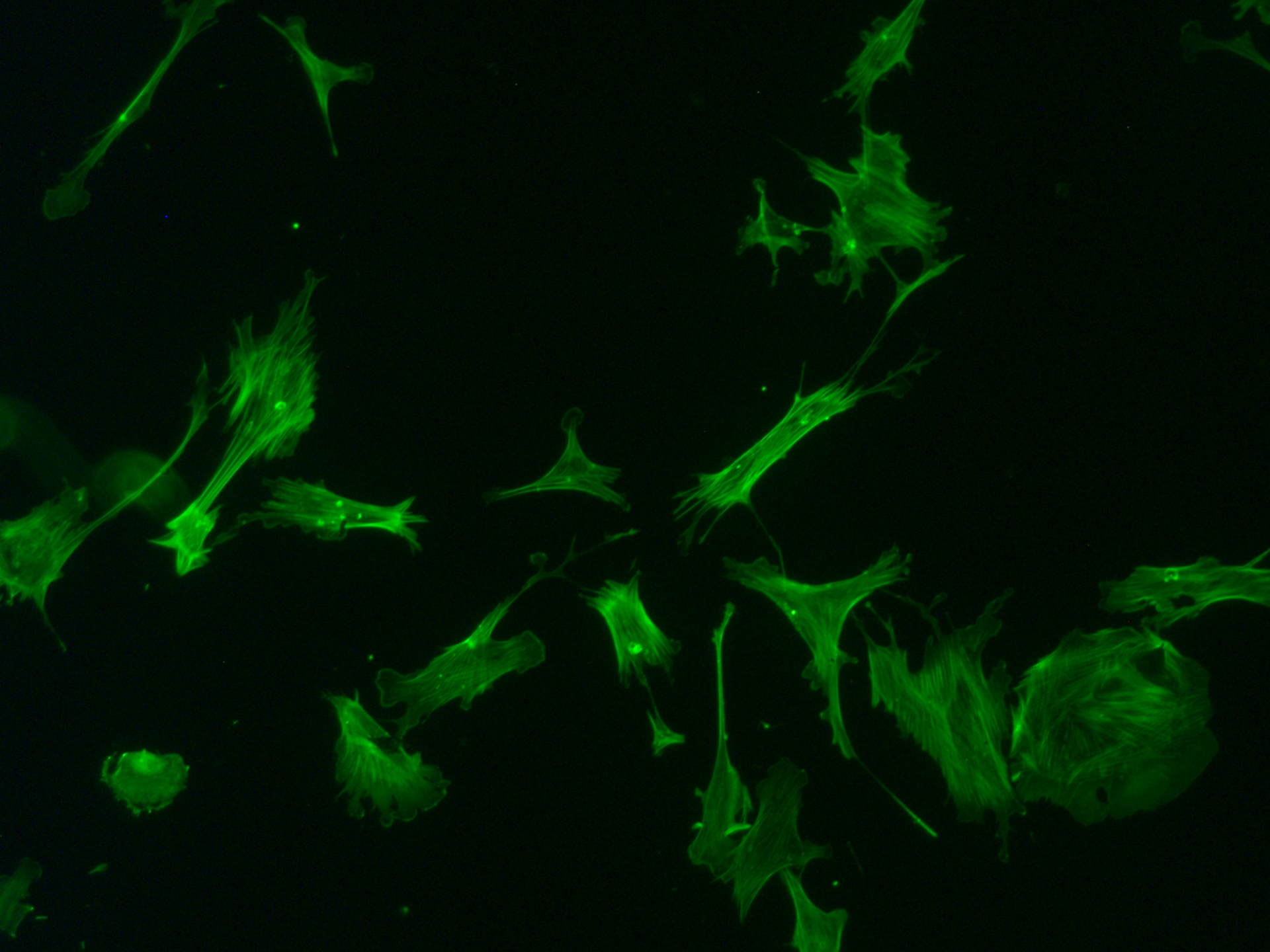

Supplement: Supplementary file 5 — Source data Fig. 2 [file 44319_2025_636_MOESM5_ESM.zip › Figure2/2B/image0010鼠1.tif]

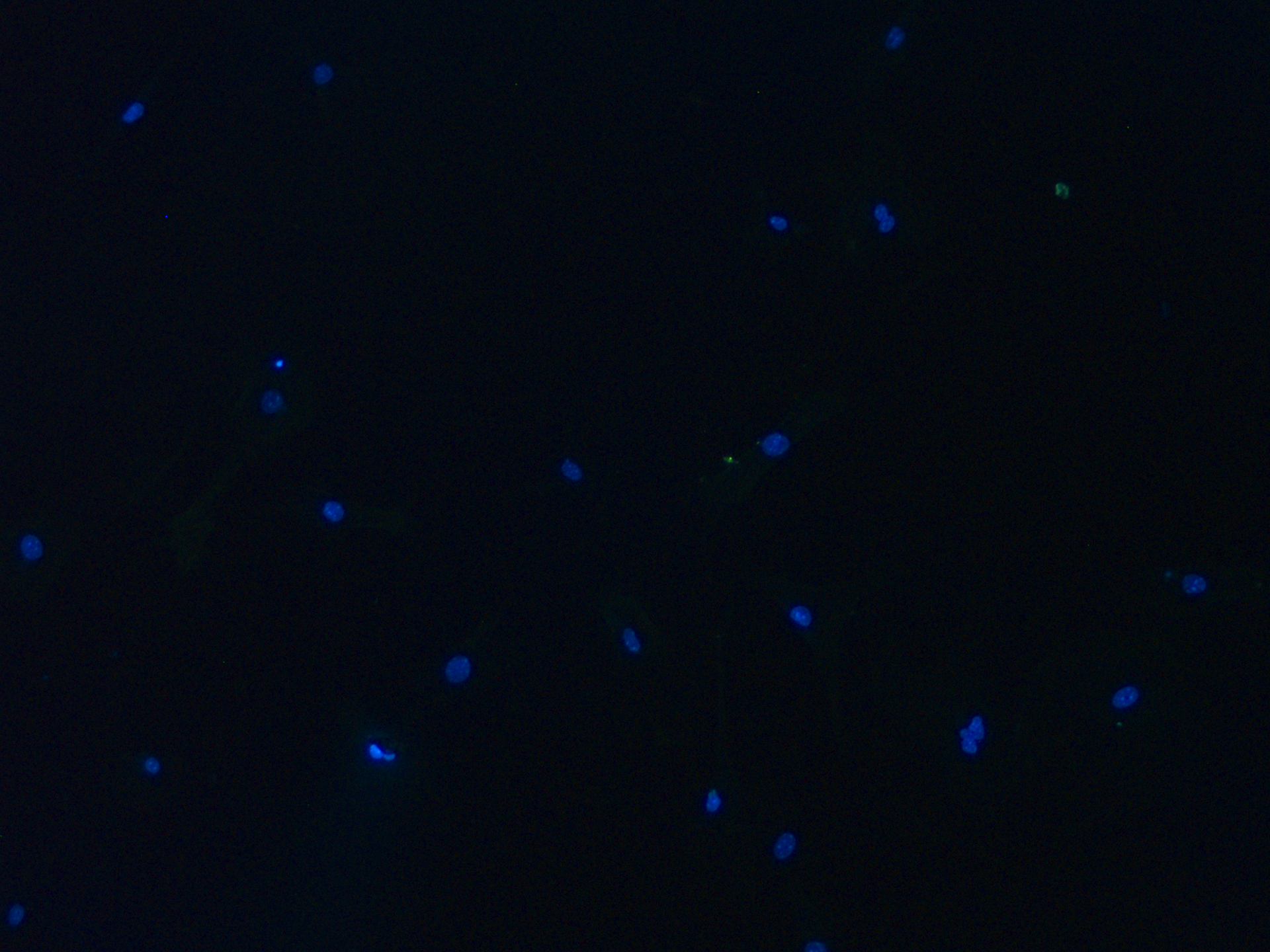

Supplement: Supplementary file 5 — Source data Fig. 2 [file 44319_2025_636_MOESM5_ESM.zip › Figure2/2B/image0011.tif]

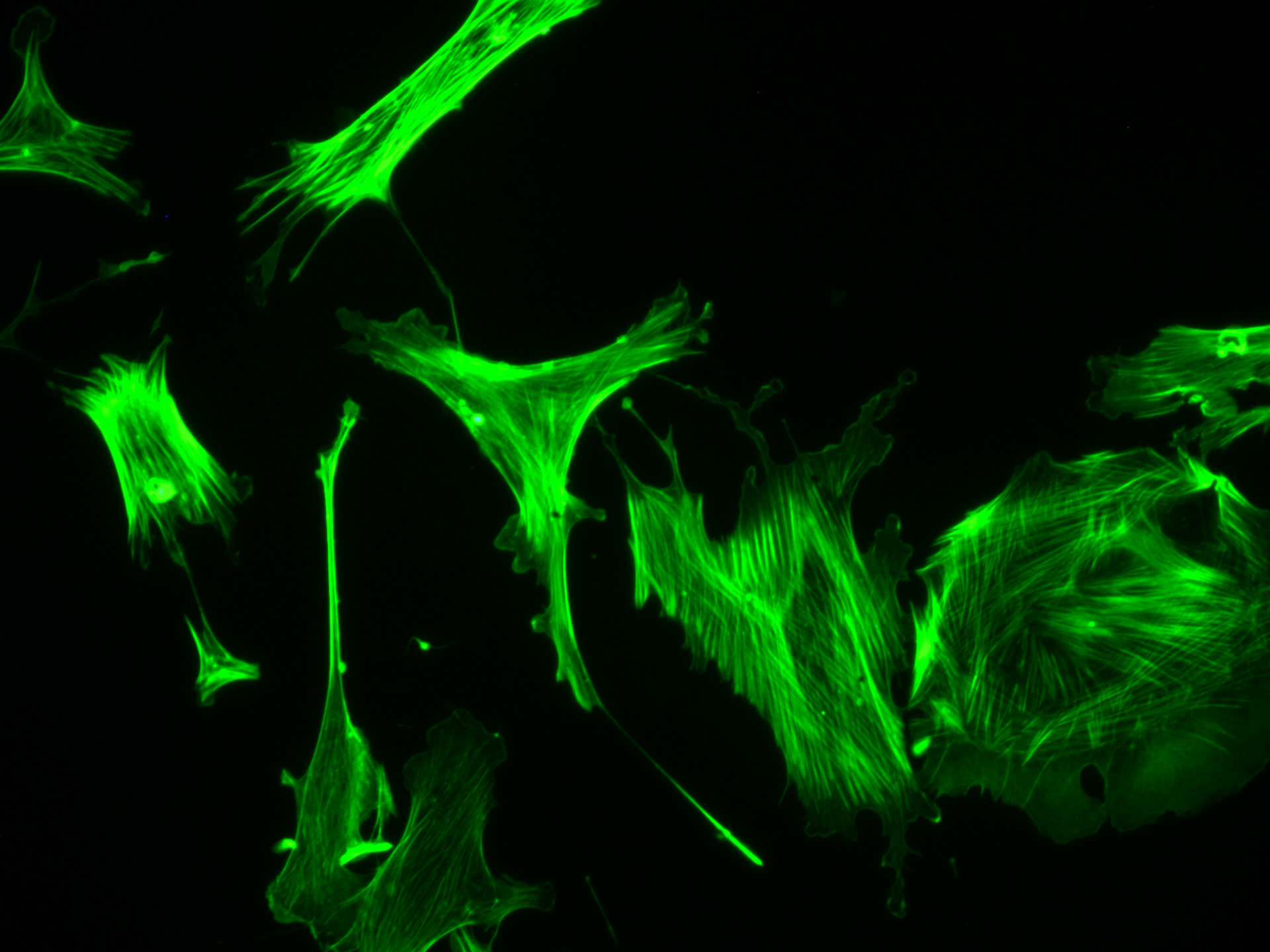

Supplement: Supplementary file 5 — Source data Fig. 2 [file 44319_2025_636_MOESM5_ESM.zip › Figure2/2B/image0012鼠.tif]

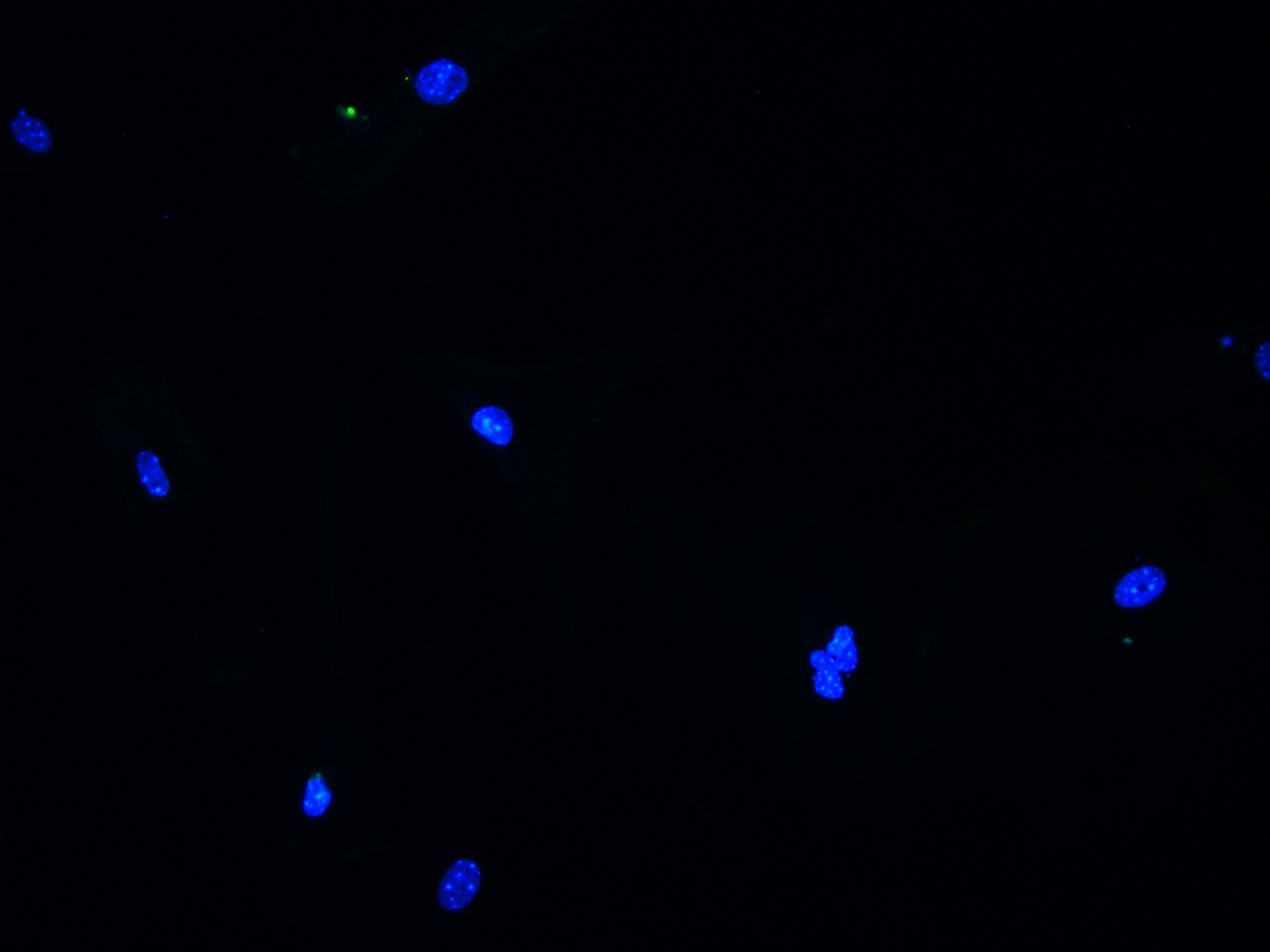

Supplement: Supplementary file 5 — Source data Fig. 2 [file 44319_2025_636_MOESM5_ESM.zip › Figure2/2B/image0013.tif]

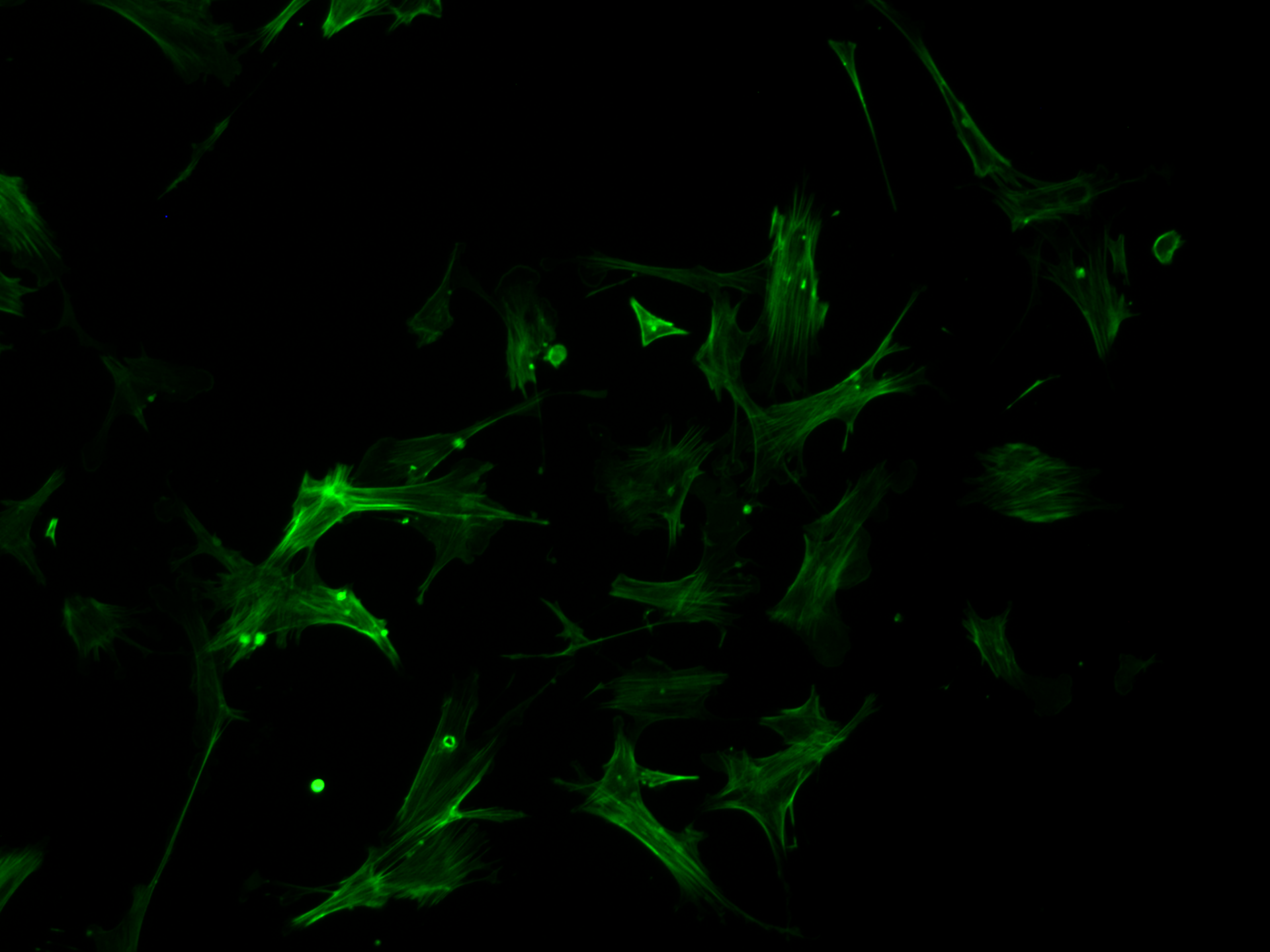

Supplement: Supplementary file 5 — Source data Fig. 2 [file 44319_2025_636_MOESM5_ESM.zip › Figure2/2B/image0014鲸1.tif]

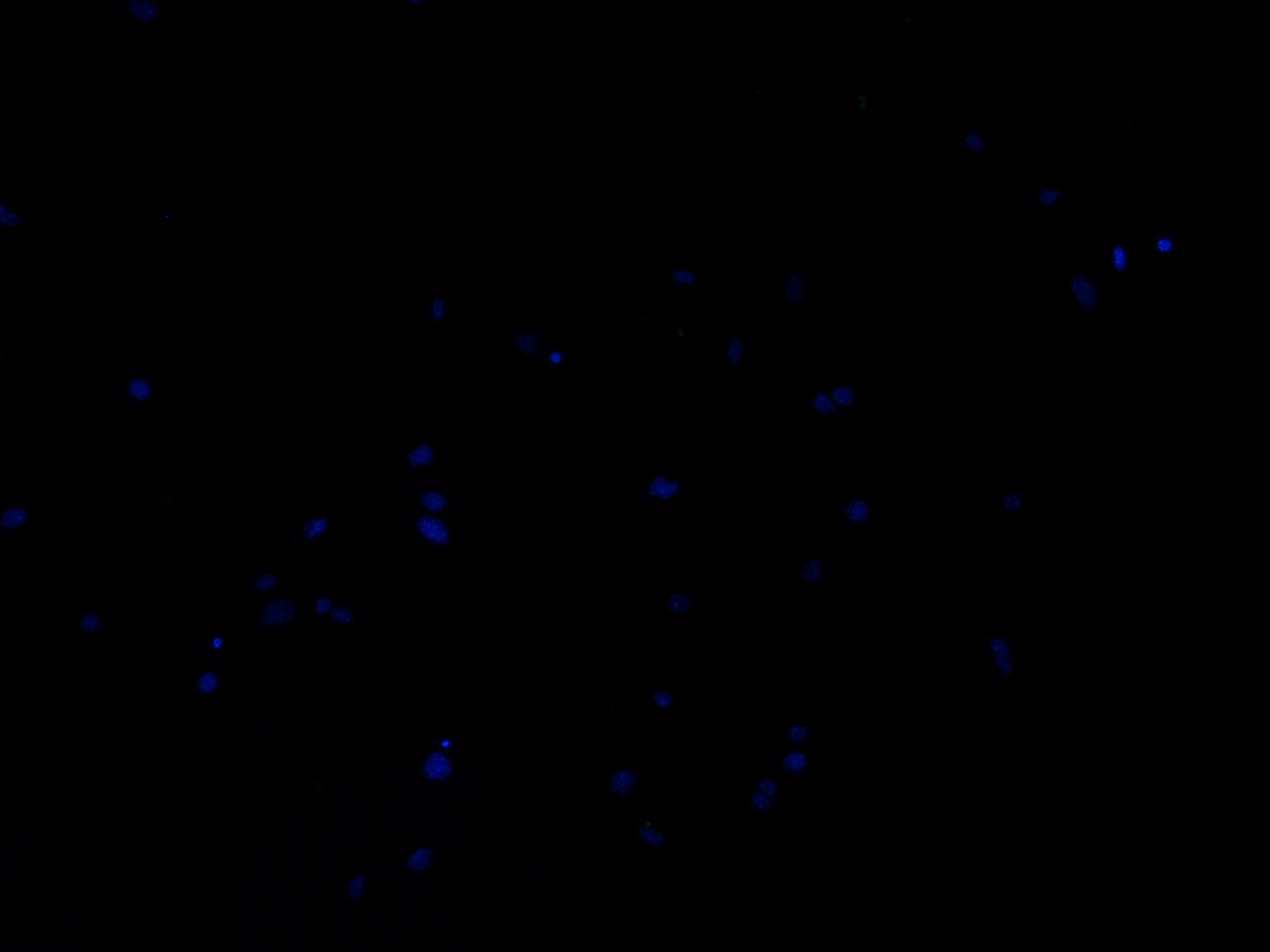

Supplement: Supplementary file 5 — Source data Fig. 2 [file 44319_2025_636_MOESM5_ESM.zip › Figure2/2B/image0015.tif]

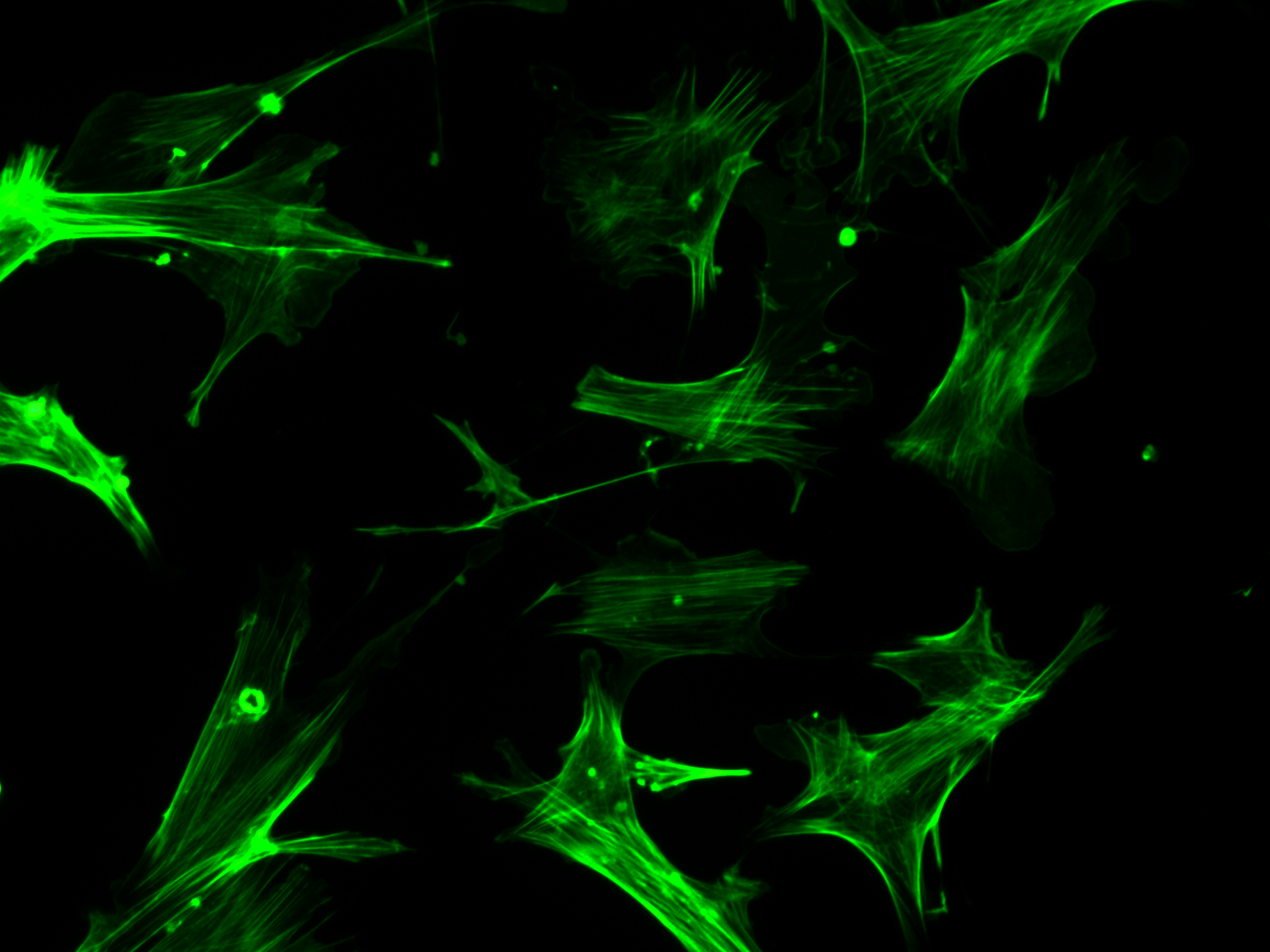

Supplement: Supplementary file 5 — Source data Fig. 2 [file 44319_2025_636_MOESM5_ESM.zip › Figure2/2B/image0016对照1.tif]

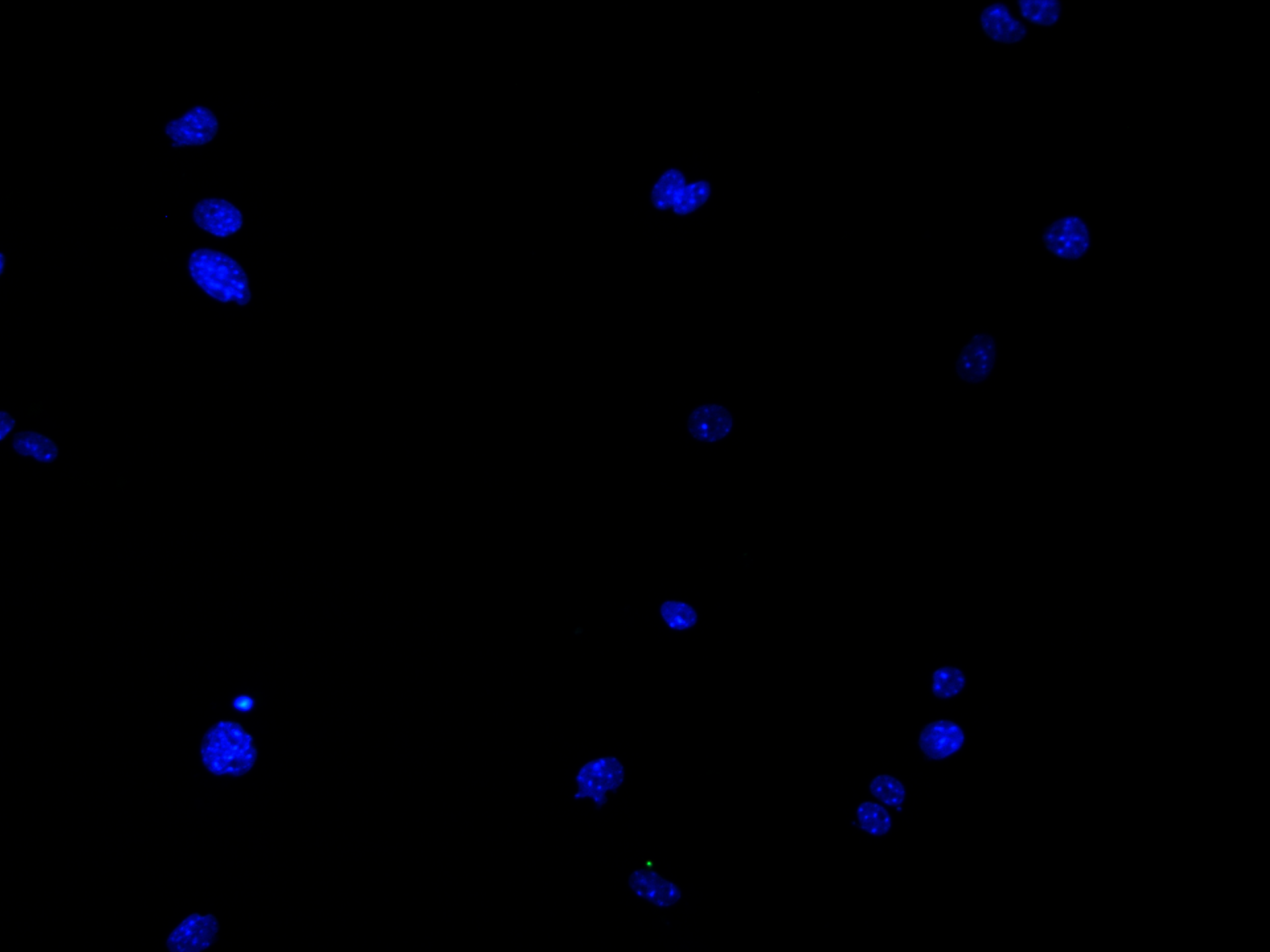

Supplement: Supplementary file 5 — Source data Fig. 2 [file 44319_2025_636_MOESM5_ESM.zip › Figure2/2B/image0017.tif]

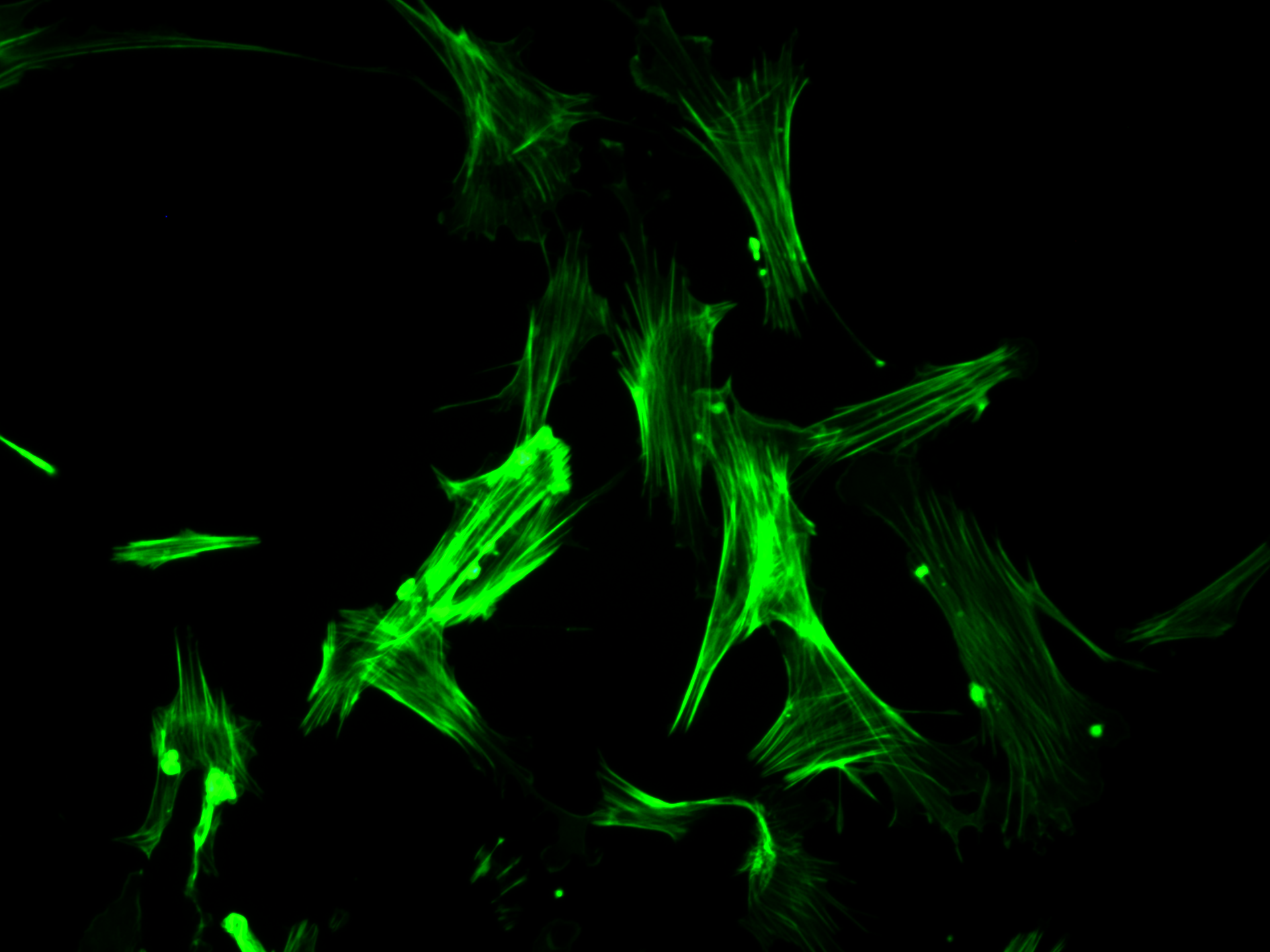

Supplement: Supplementary file 5 — Source data Fig. 2 [file 44319_2025_636_MOESM5_ESM.zip › Figure2/2B/image0018鼠.tif]

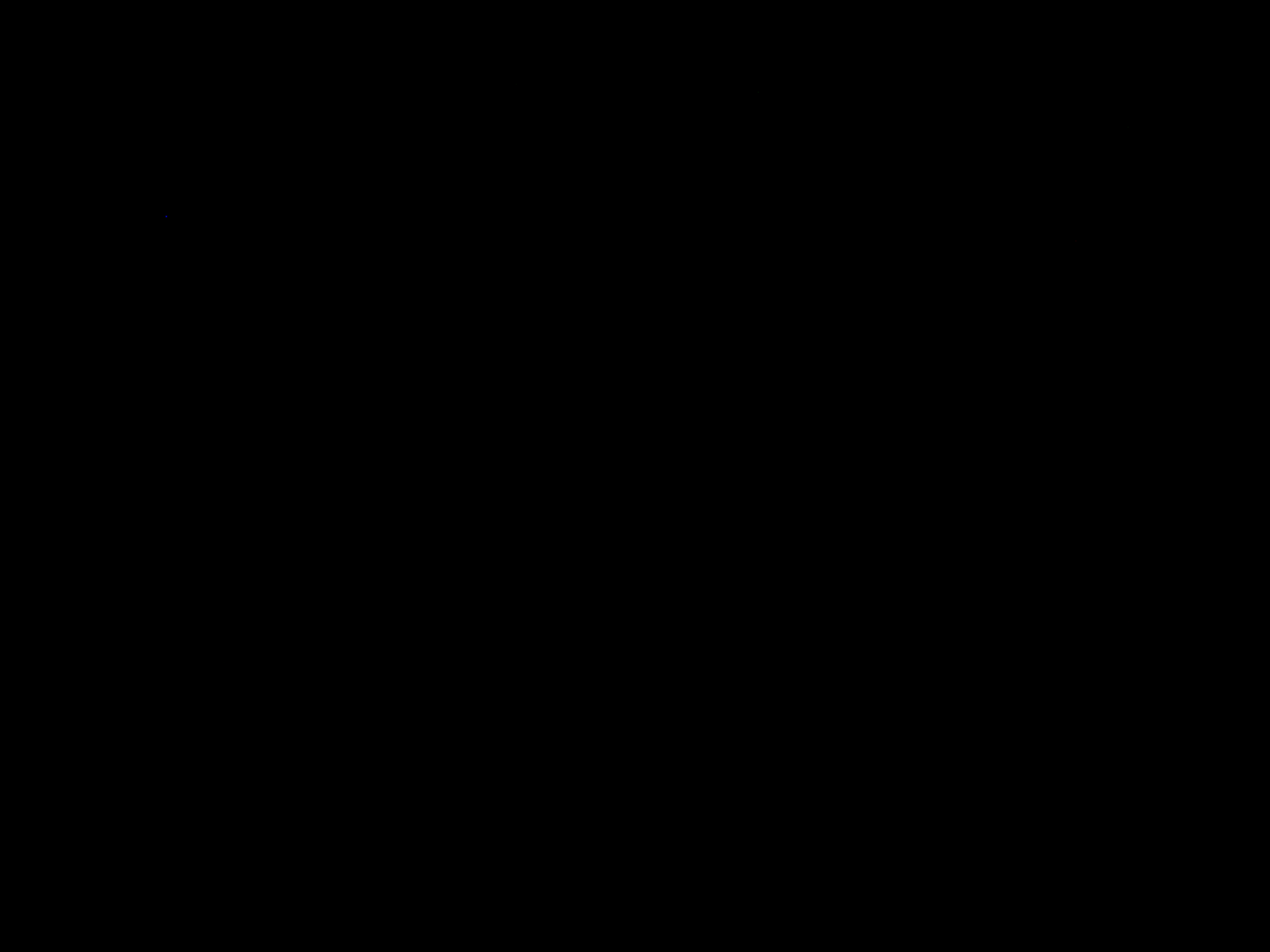

Supplement: Supplementary file 5 — Source data Fig. 2 [file 44319_2025_636_MOESM5_ESM.zip › Figure2/2B/image0019.tif]

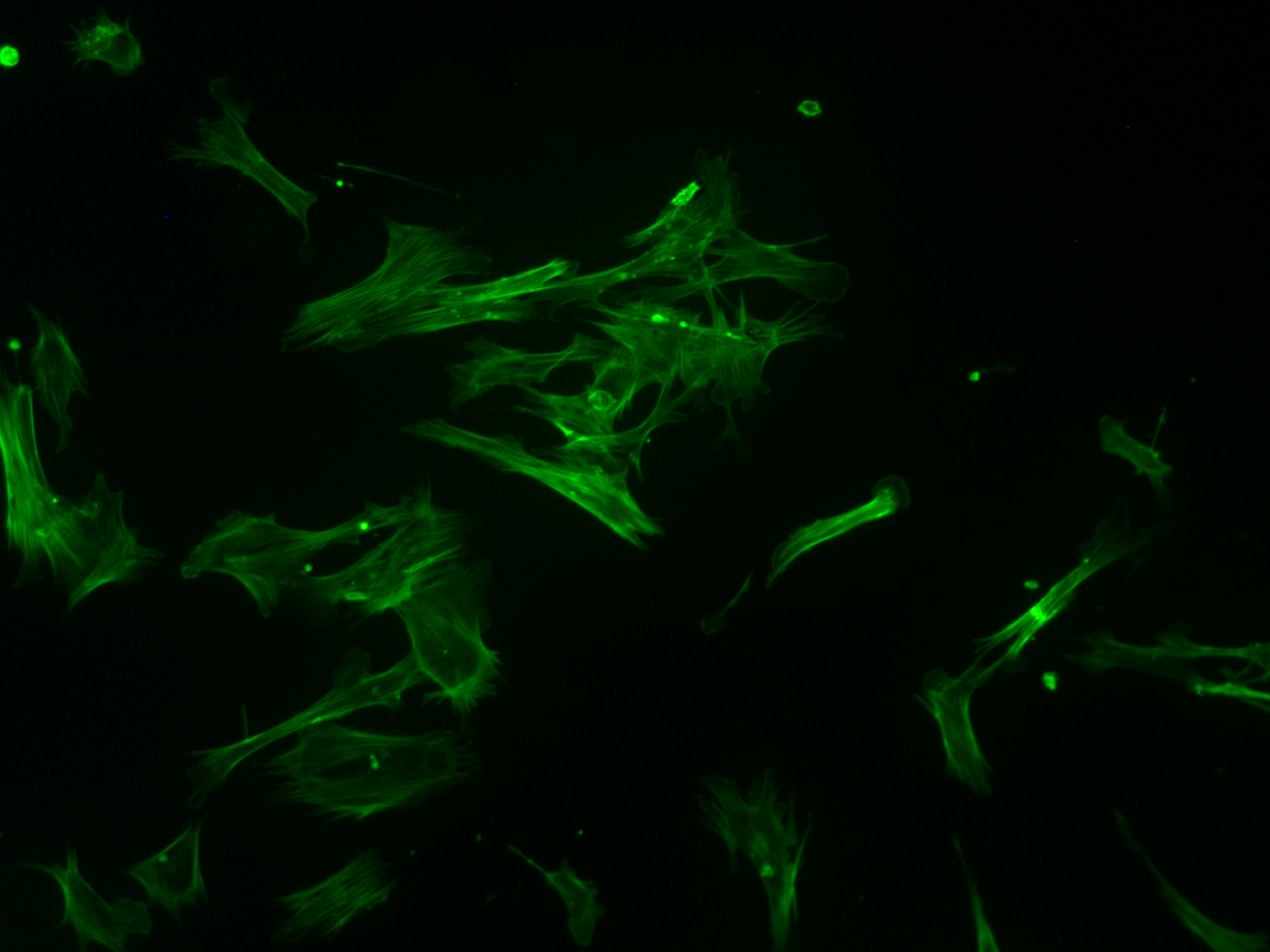

Supplement: Supplementary file 5 — Source data Fig. 2 [file 44319_2025_636_MOESM5_ESM.zip › Figure2/2B/image0020对照1.tif]

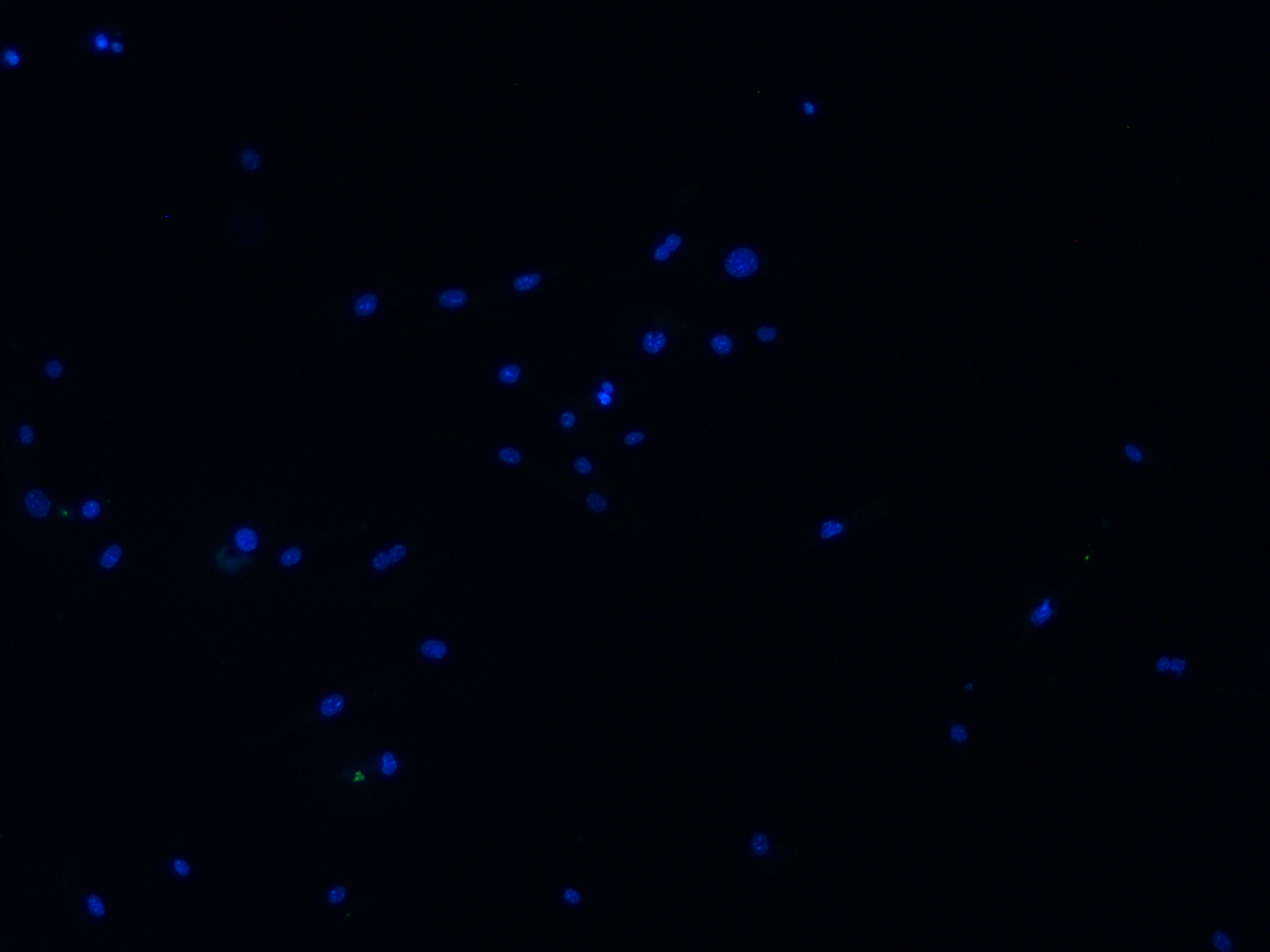

Supplement: Supplementary file 5 — Source data Fig. 2 [file 44319_2025_636_MOESM5_ESM.zip › Figure2/2B/image0021.tif]

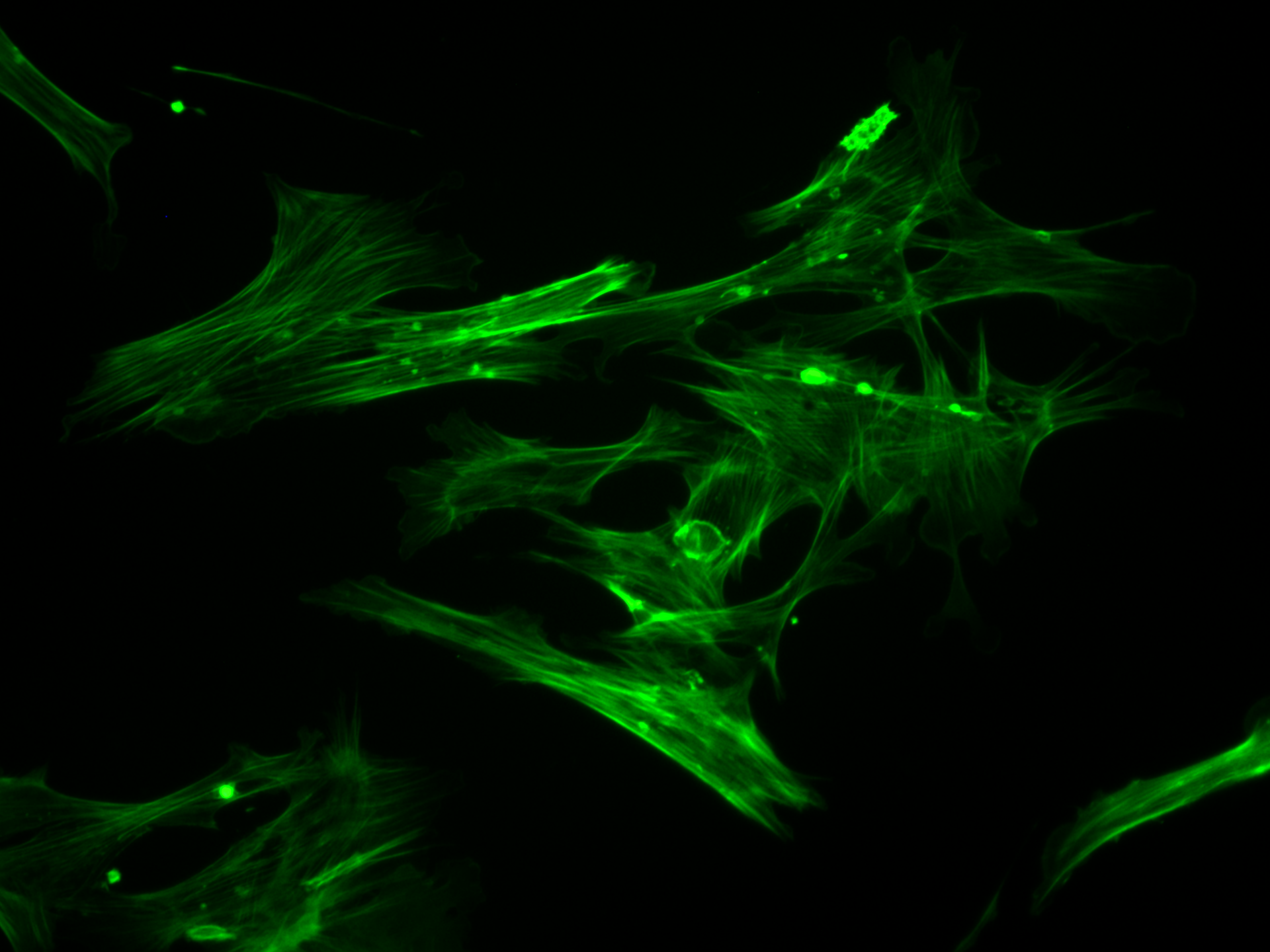

Supplement: Supplementary file 5 — Source data Fig. 2 [file 44319_2025_636_MOESM5_ESM.zip › Figure2/2B/image0022鲸.tif]

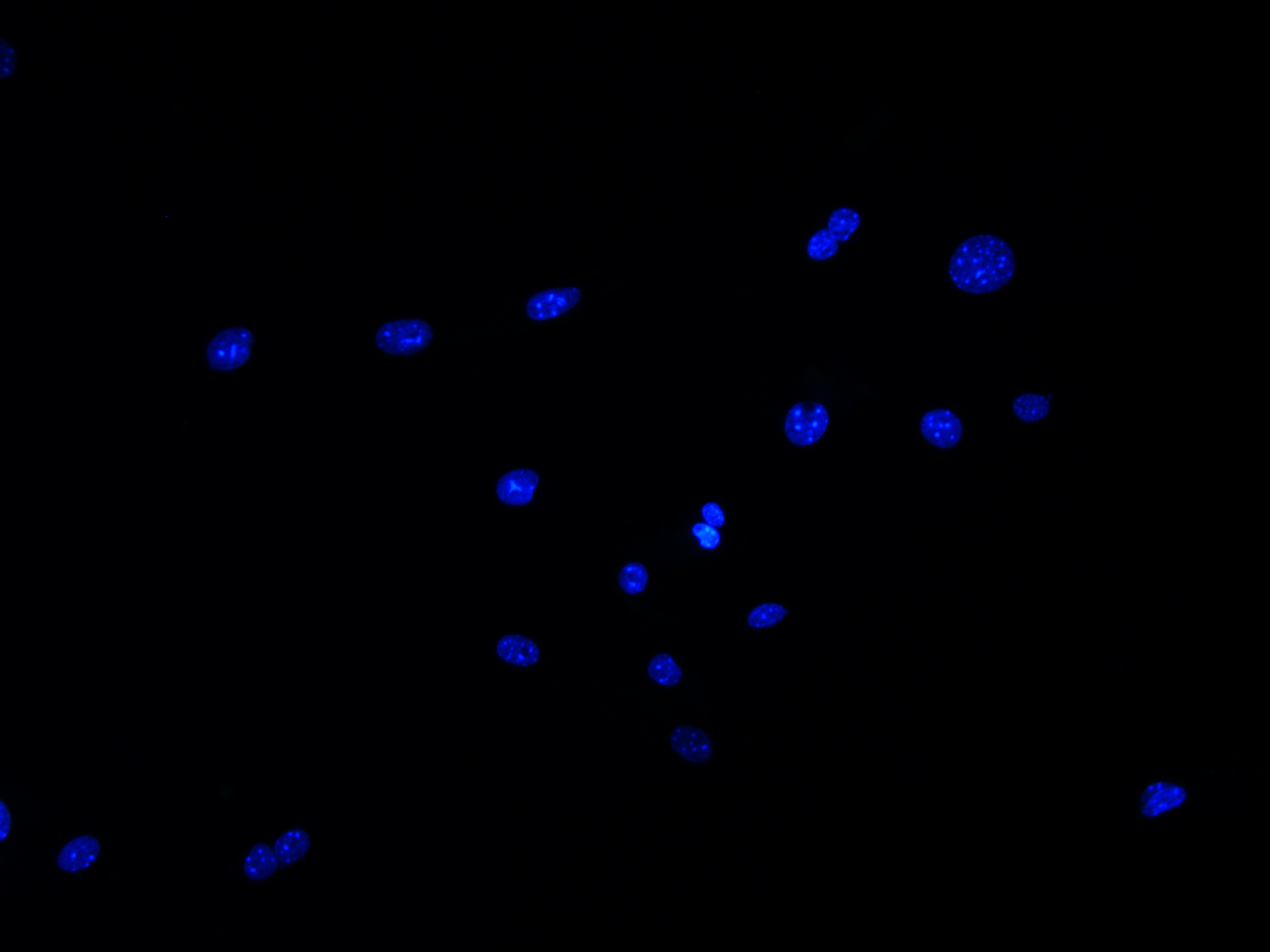

Supplement: Supplementary file 5 — Source data Fig. 2 [file 44319_2025_636_MOESM5_ESM.zip › Figure2/2B/image0023.tif]

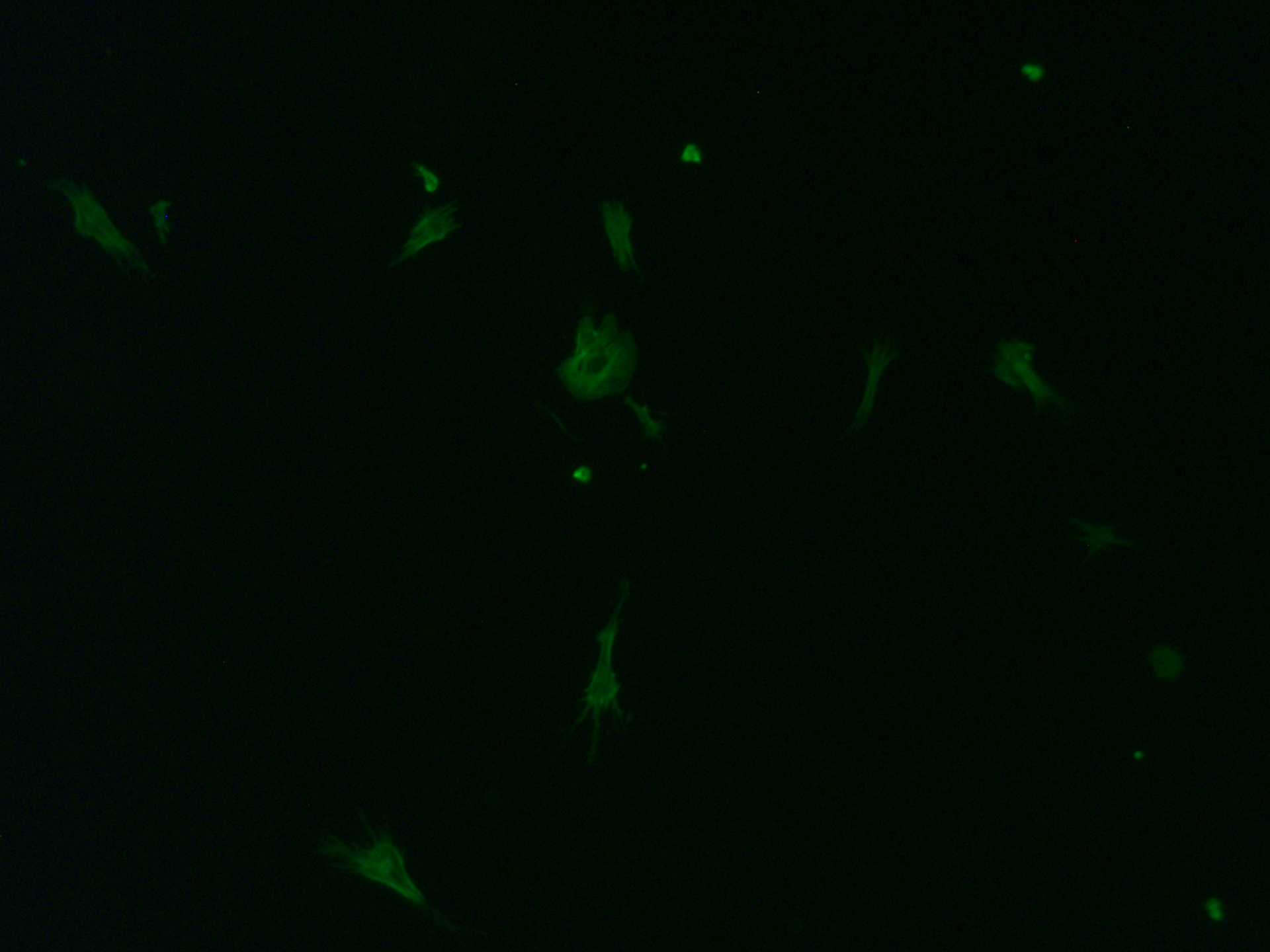

Supplement: Supplementary file 5 — Source data Fig. 2 [file 44319_2025_636_MOESM5_ESM.zip › Figure2/2B/image对照1.tif]

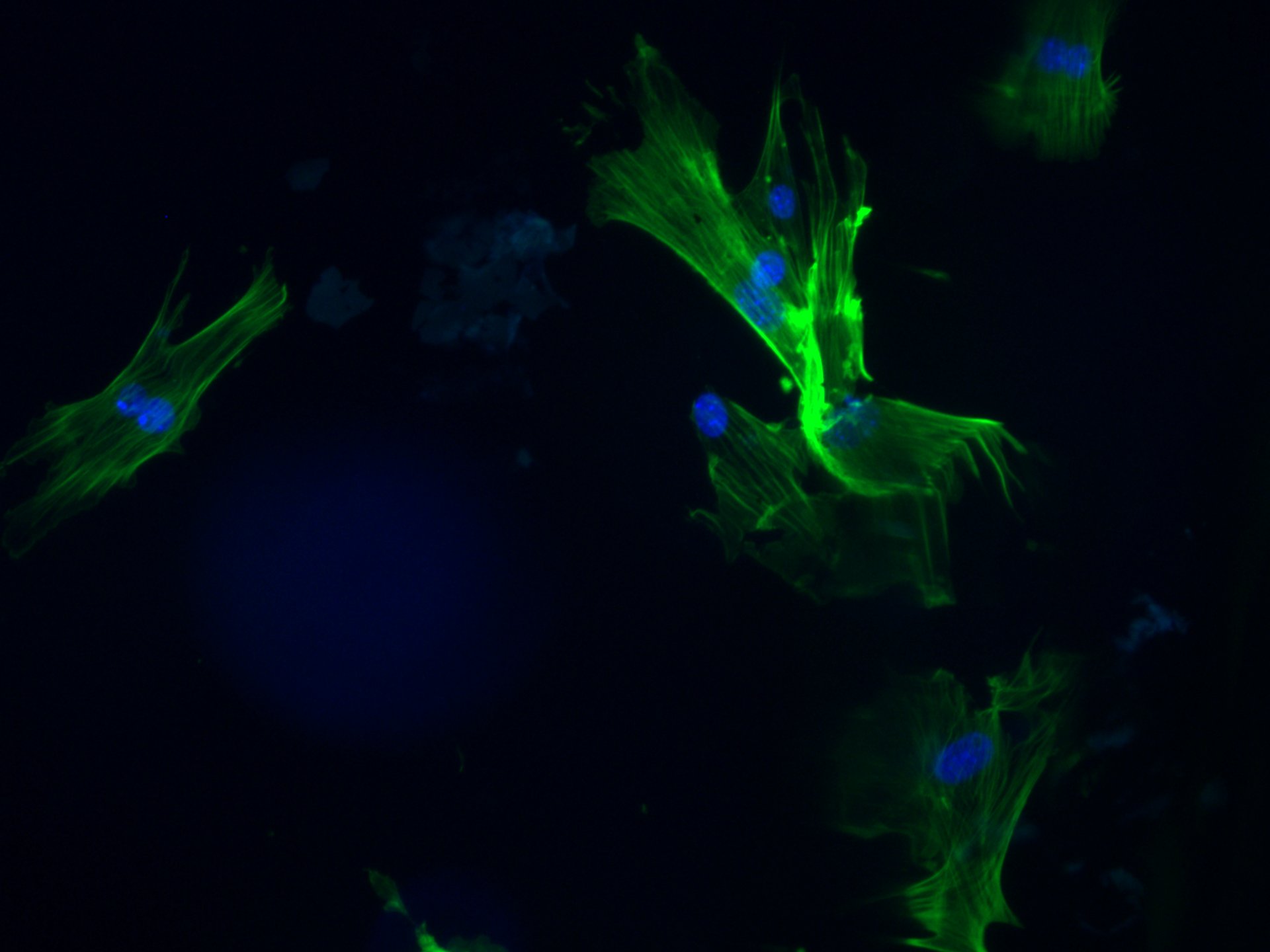

Supplement: Supplementary file 5 — Source data Fig. 2 [file 44319_2025_636_MOESM5_ESM.zip › Figure2/2B/对照24h.jpg]

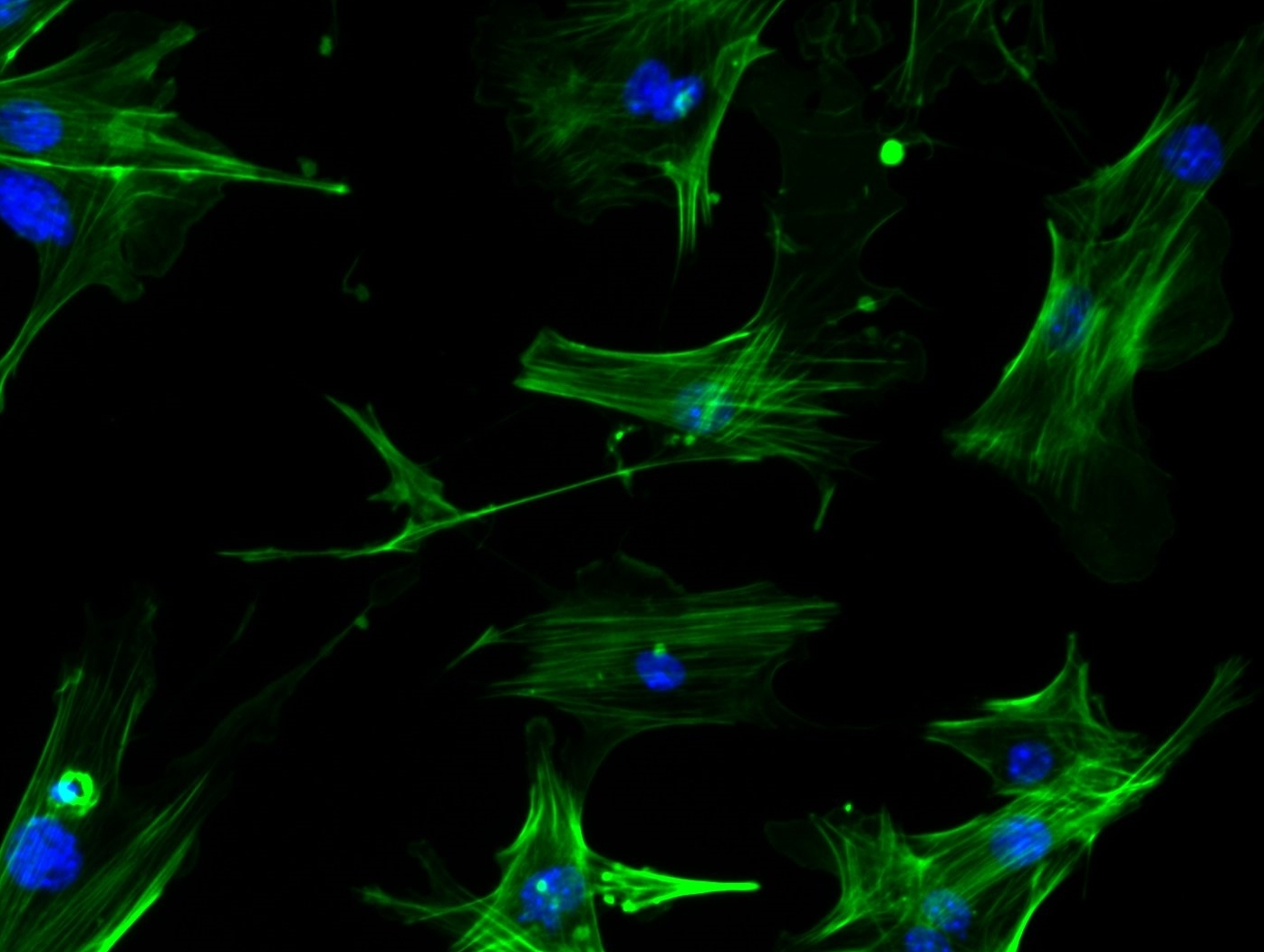

Supplement: Supplementary file 5 — Source data Fig. 2 [file 44319_2025_636_MOESM5_ESM.zip › Figure2/2B/对照48h.jpg]

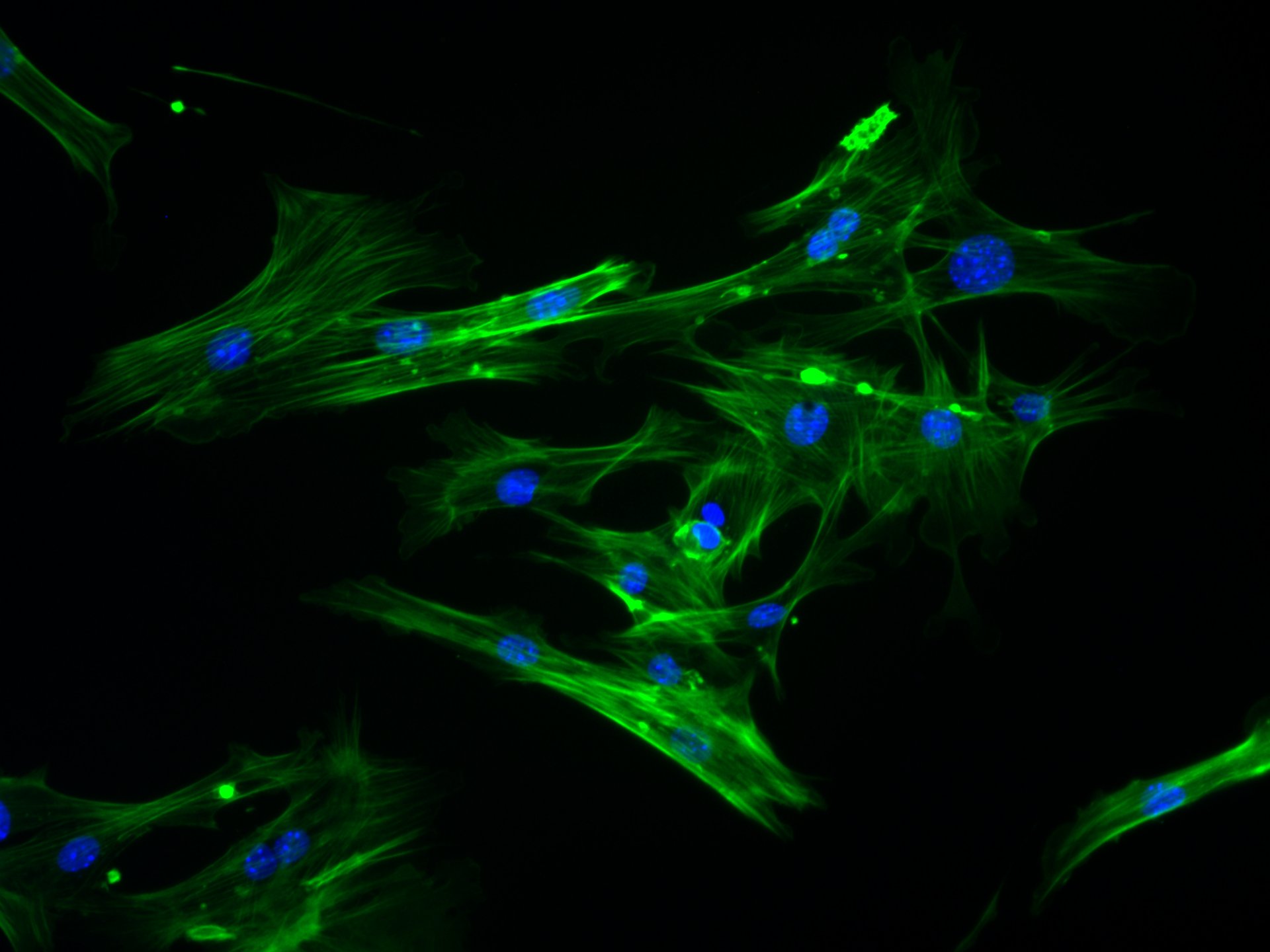

Supplement: Supplementary file 5 — Source data Fig. 2 [file 44319_2025_636_MOESM5_ESM.zip › Figure2/2B/鲸24h.jpg]

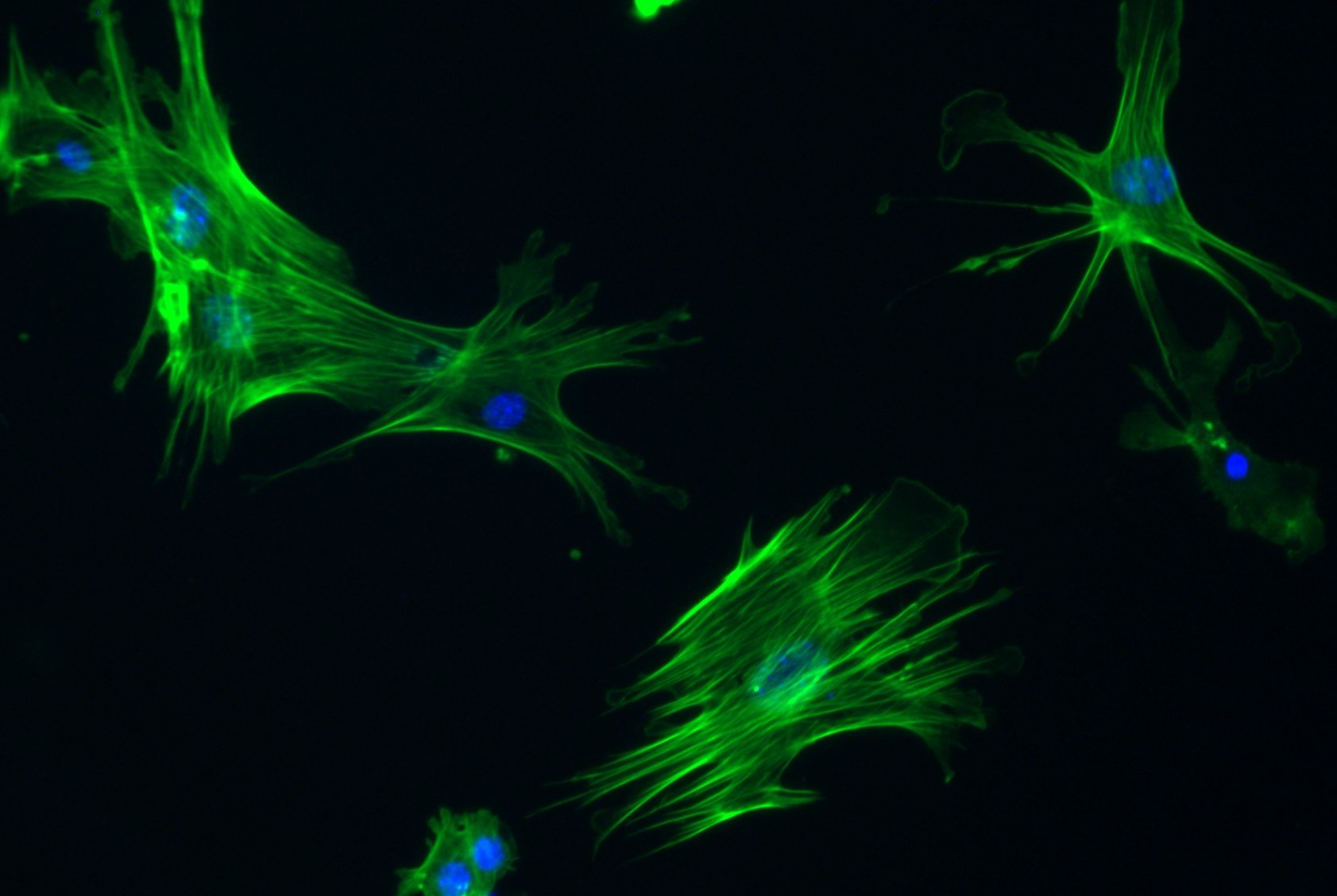

Supplement: Supplementary file 5 — Source data Fig. 2 [file 44319_2025_636_MOESM5_ESM.zip › Figure2/2B/鲸48h.jpg]

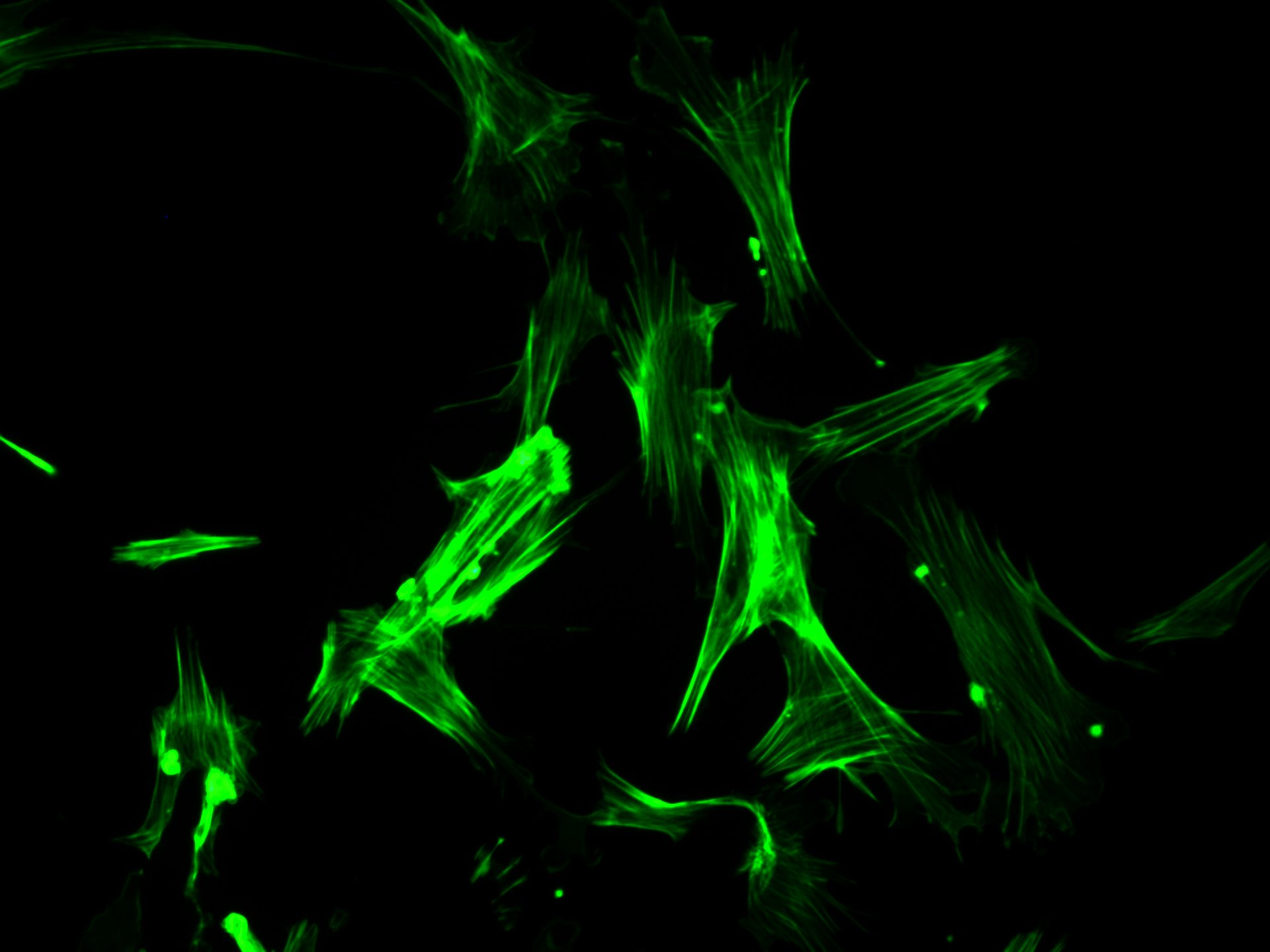

Supplement: Supplementary file 5 — Source data Fig. 2 [file 44319_2025_636_MOESM5_ESM.zip › Figure2/2B/鼠24h.jpg]

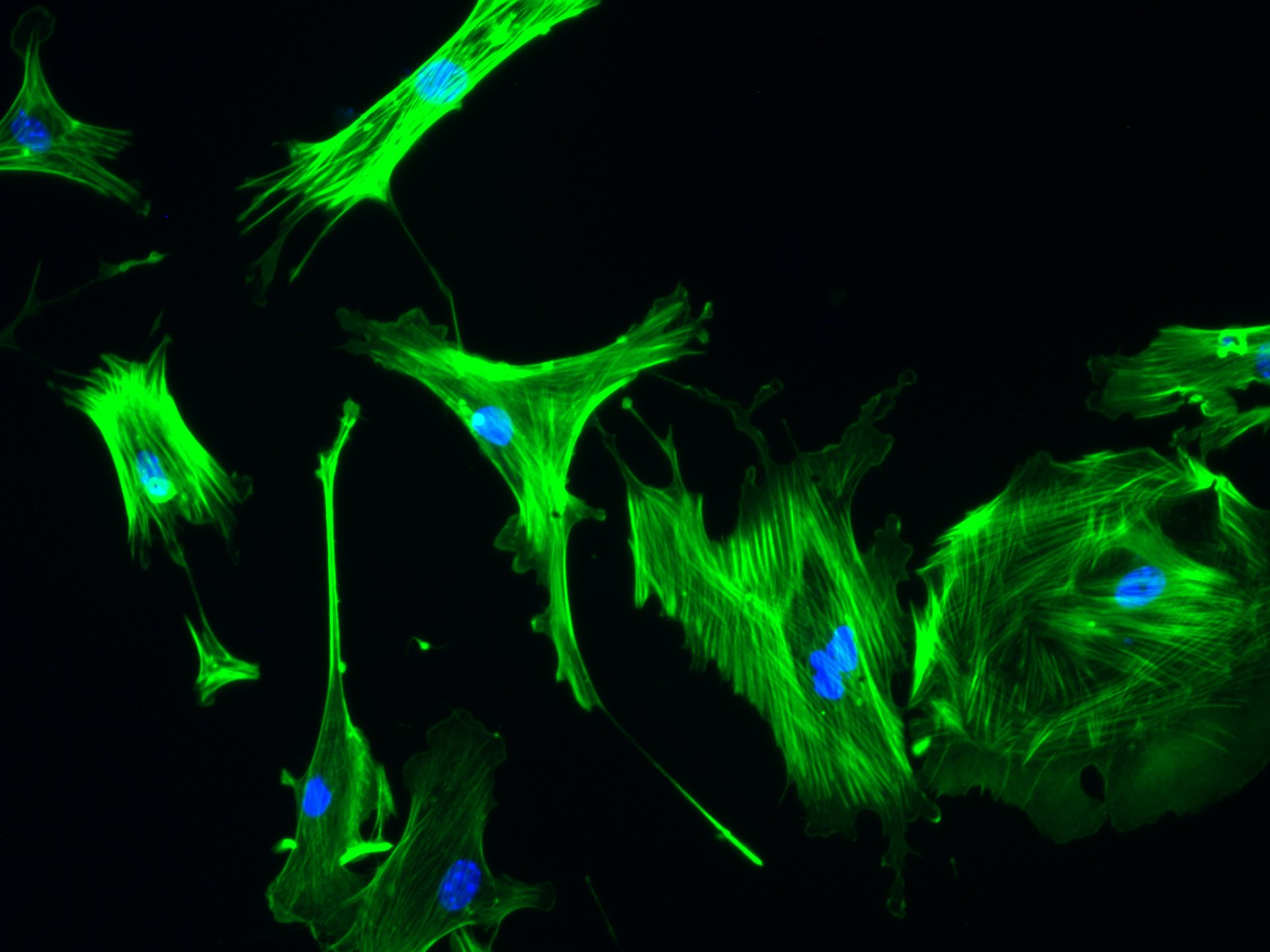

Supplement: Supplementary file 5 — Source data Fig. 2 [file 44319_2025_636_MOESM5_ESM.zip › Figure2/2B/鼠48h.jpg]

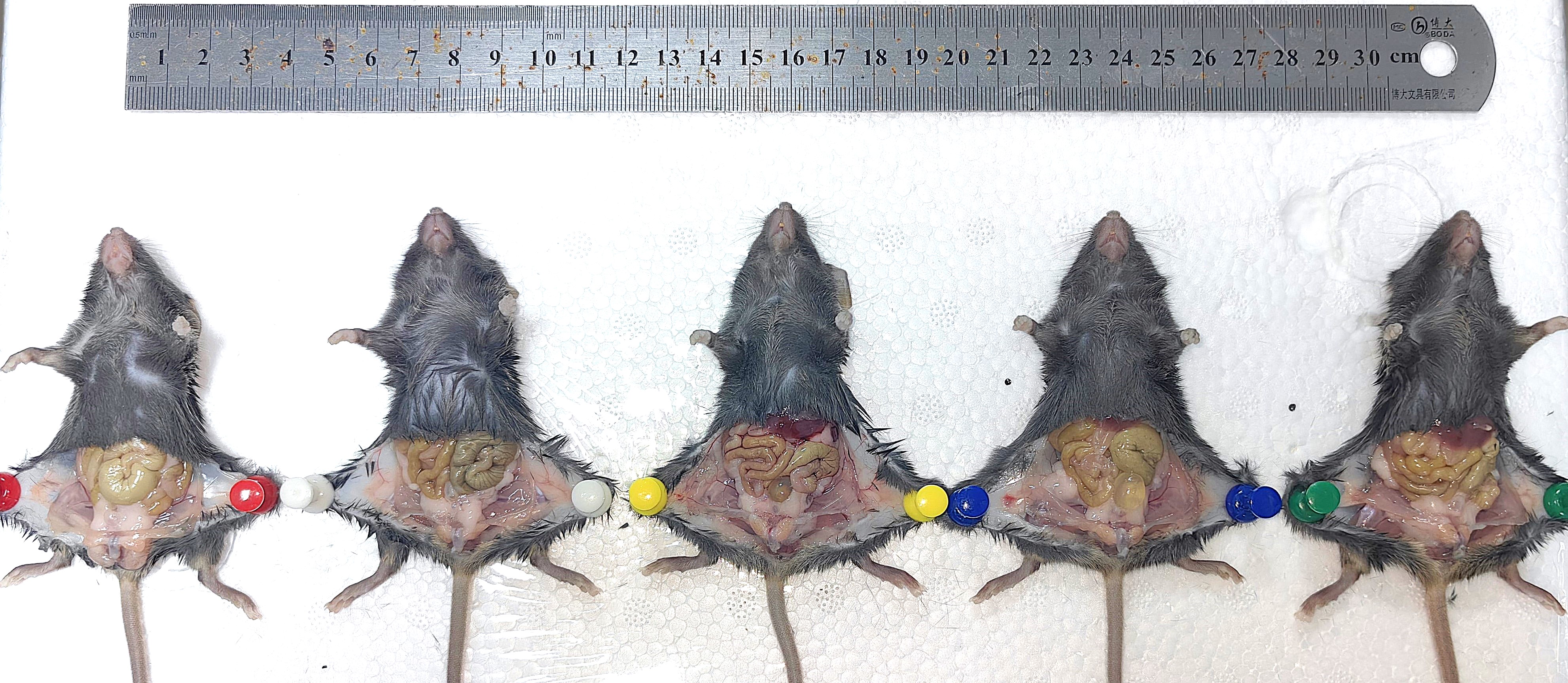

Supplement: Supplementary file 6 — Source data Fig. 3 [file 44319_2025_636_MOESM6_ESM.zip › Figure3/3A/fig S2.jpg]

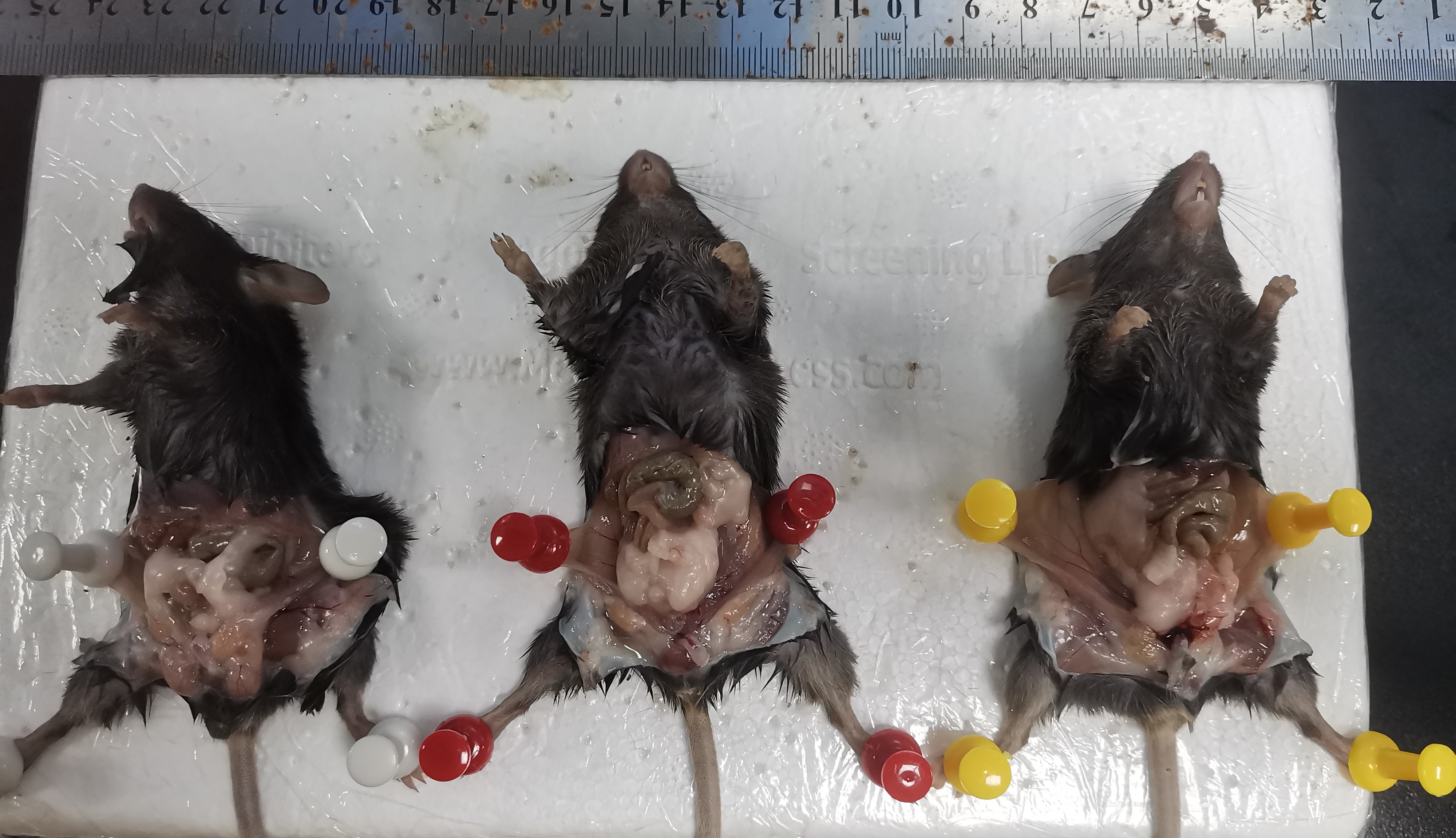

Supplement: Supplementary file 6 — Source data Fig. 3 [file 44319_2025_636_MOESM6_ESM.zip › Figure3/3A/IMG_20230817_170436(1).jpg]

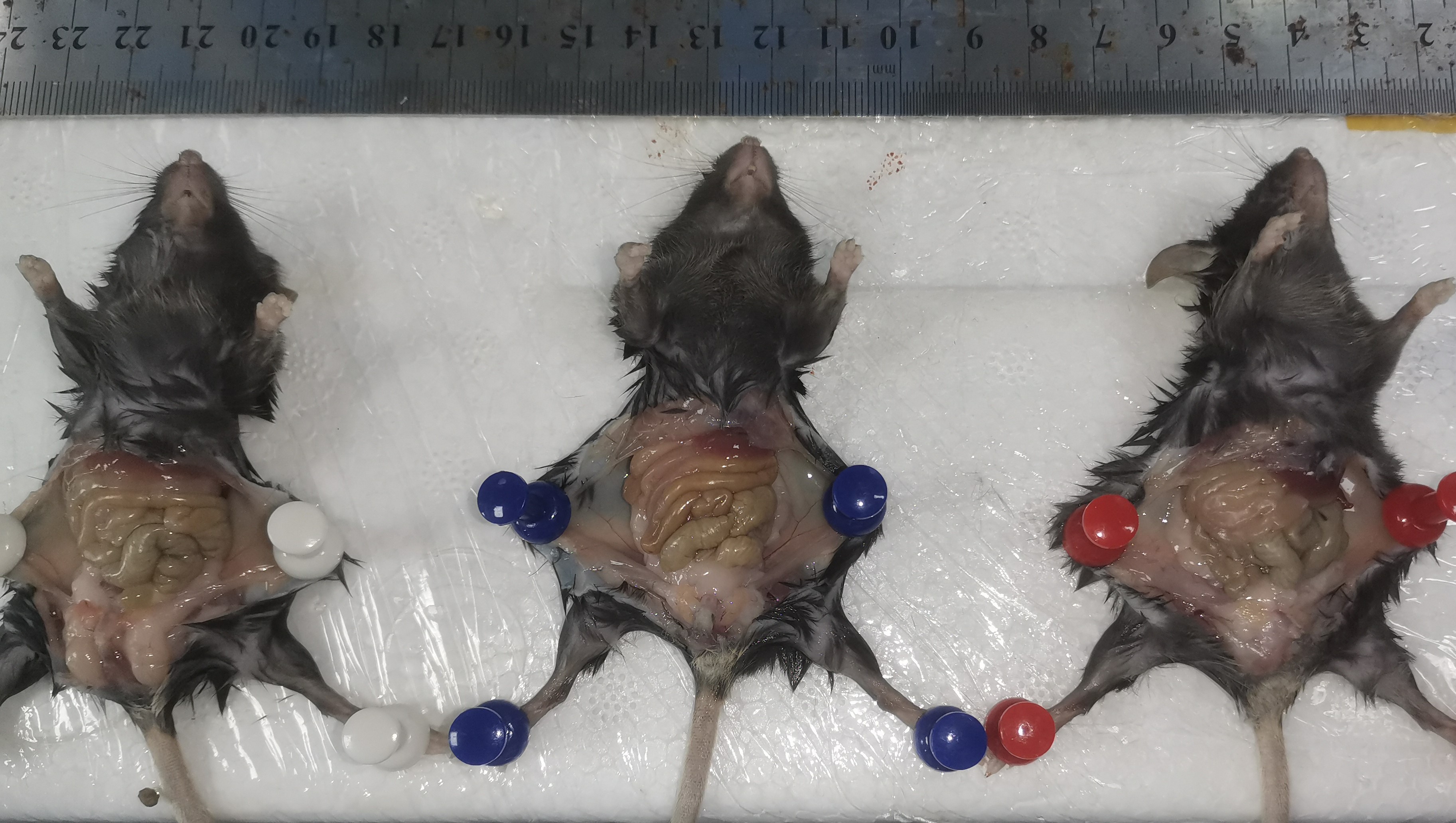

Supplement: Supplementary file 6 — Source data Fig. 3 [file 44319_2025_636_MOESM6_ESM.zip › Figure3/3A/Repeat the experiment.jpg]

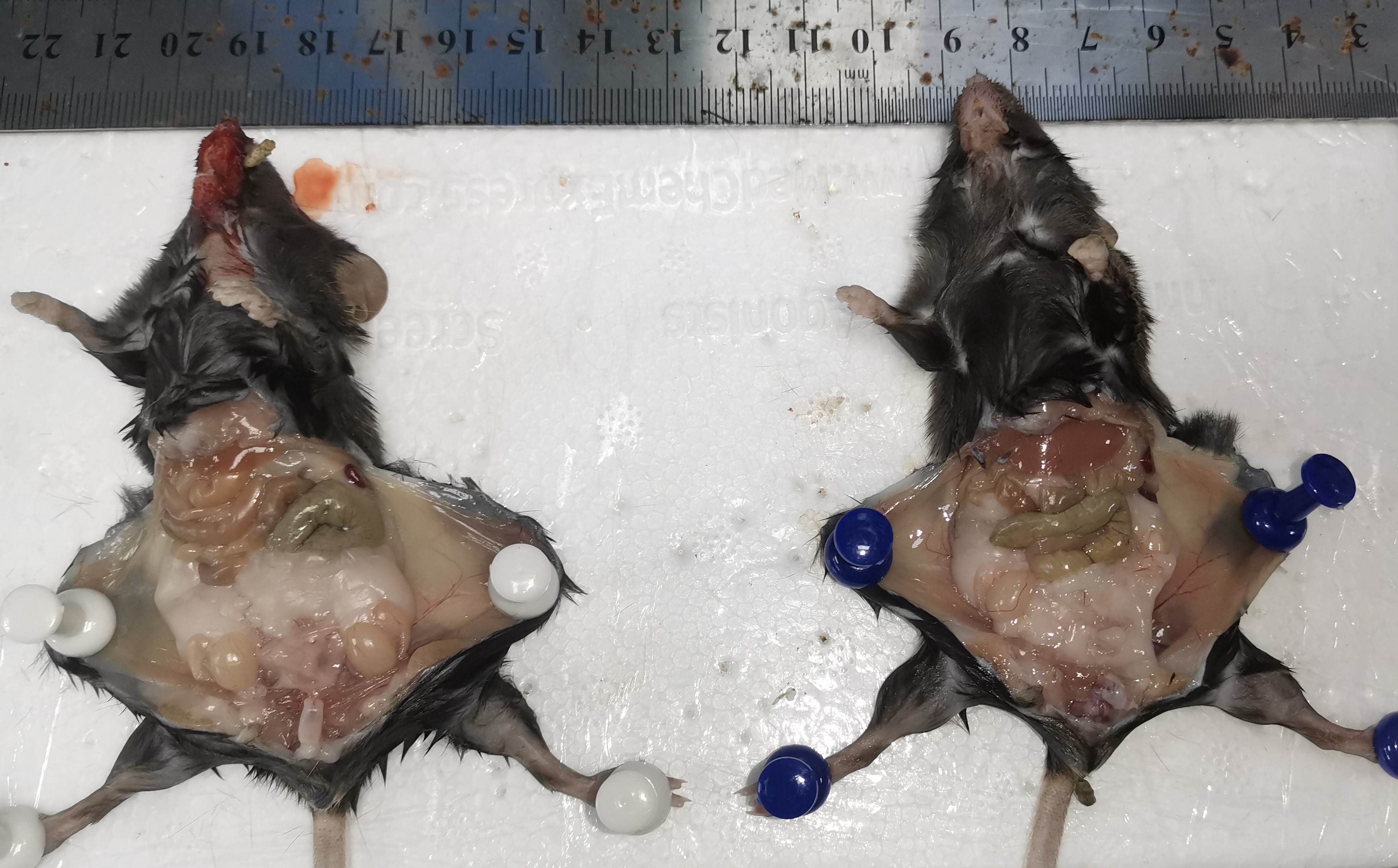

Supplement: Supplementary file 6 — Source data Fig. 3 [file 44319_2025_636_MOESM6_ESM.zip › Figure3/3A/WT V.S. INSL3-1.jpg]

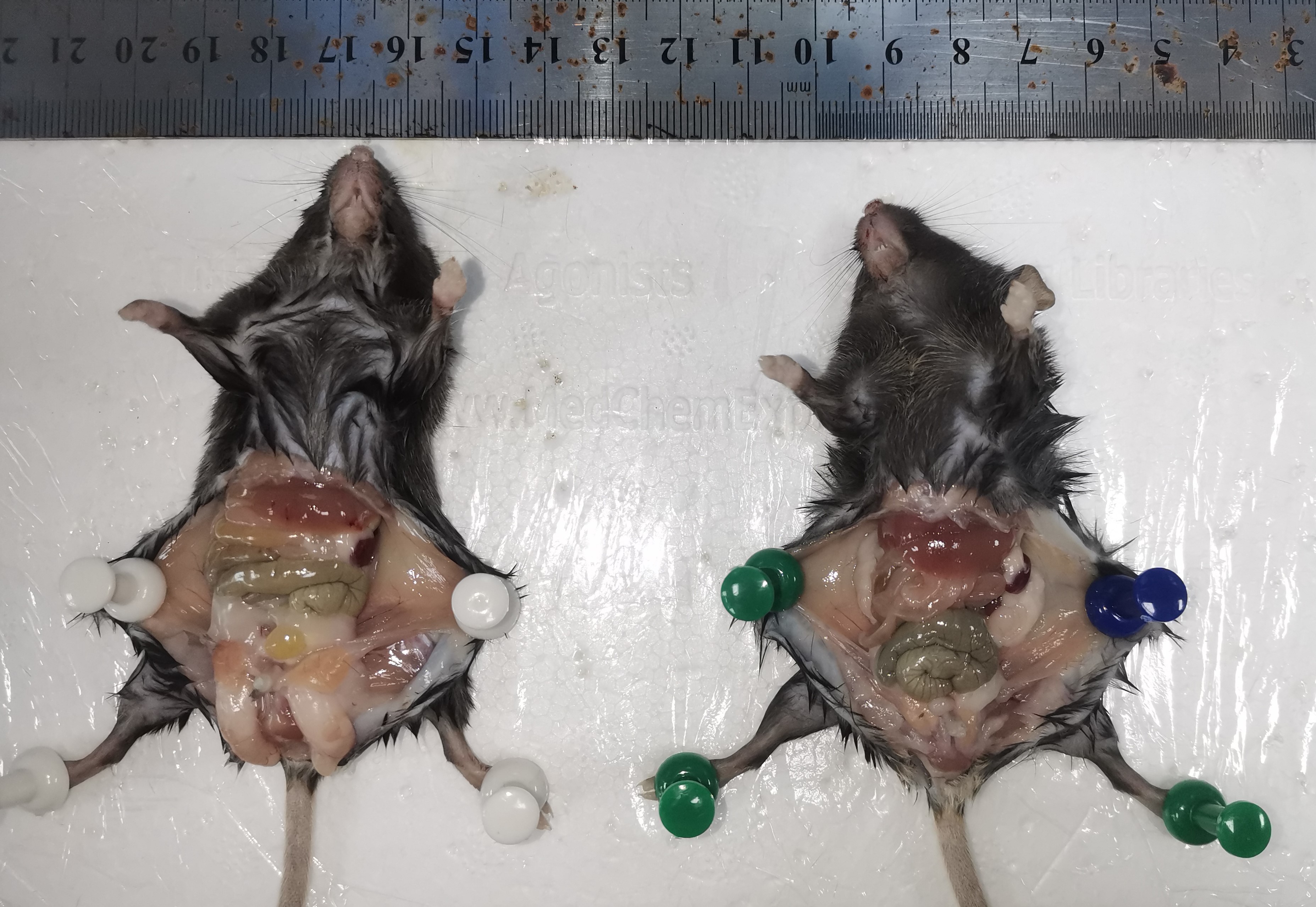

Supplement: Supplementary file 6 — Source data Fig. 3 [file 44319_2025_636_MOESM6_ESM.zip › Figure3/3A/WT V.S. KO-2.jpg]

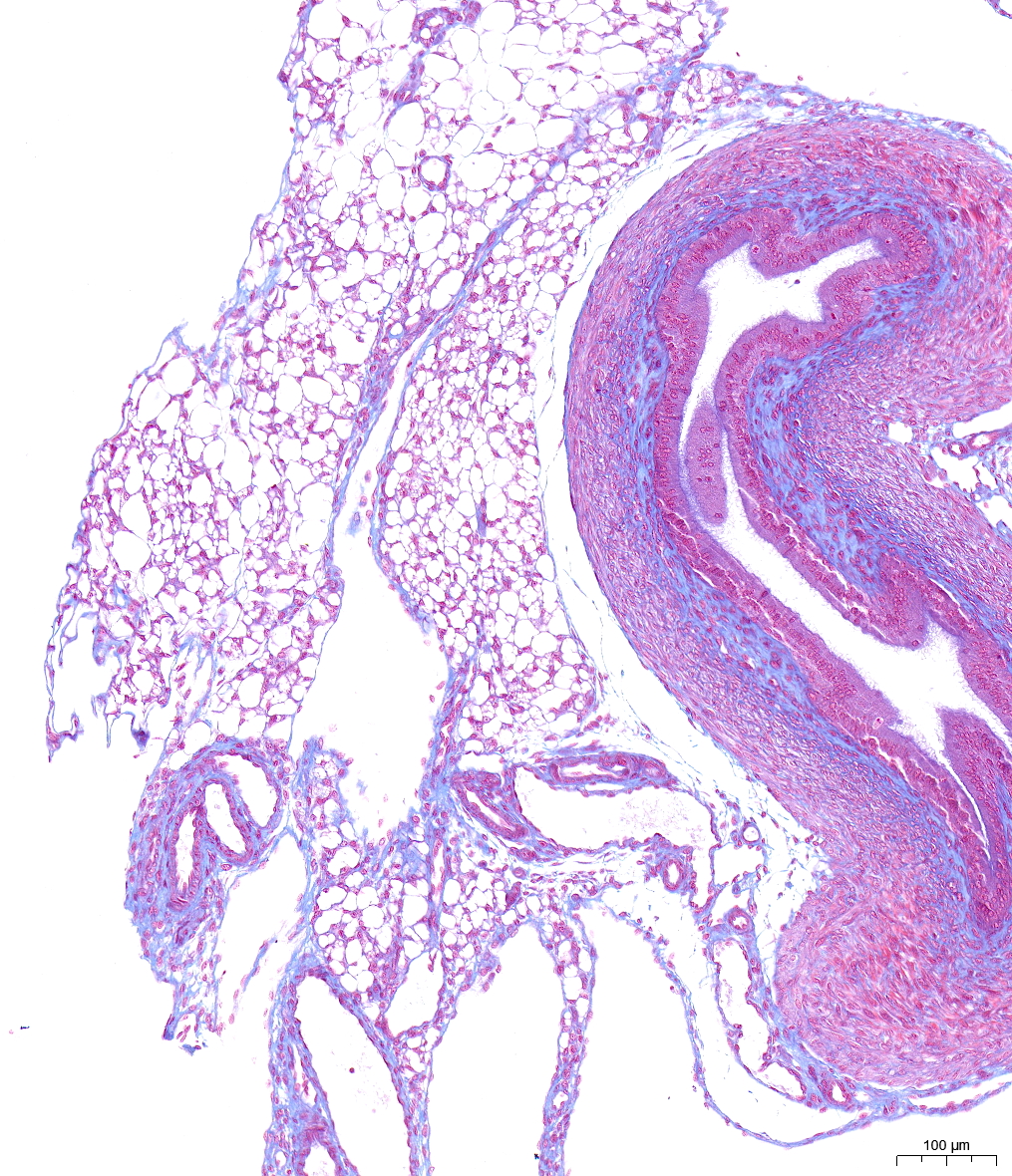

Supplement: Supplementary file 6 — Source data Fig. 3 [file 44319_2025_636_MOESM6_ESM.zip › Figure3/3C/ki.jpg]

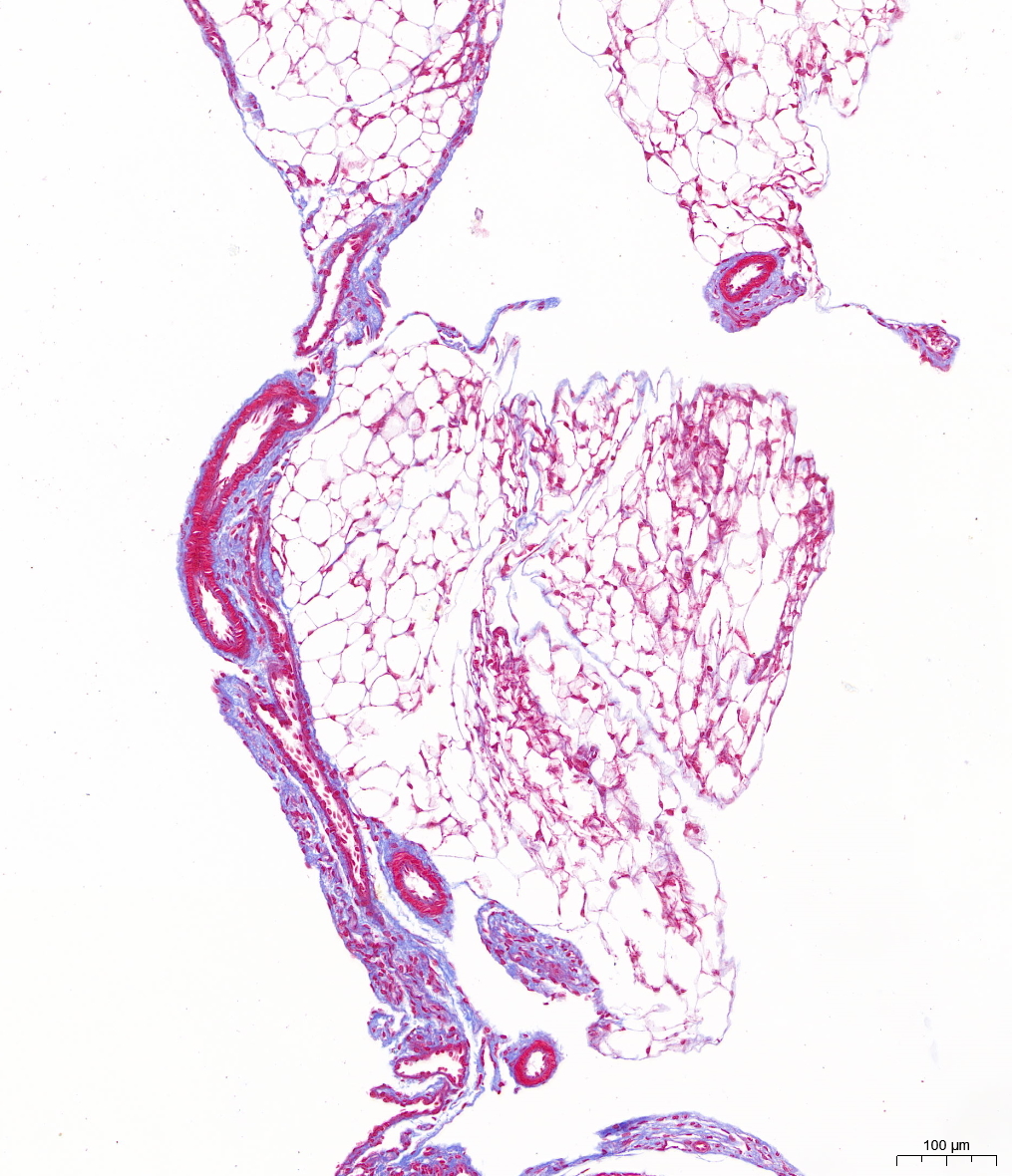

Supplement: Supplementary file 6 — Source data Fig. 3 [file 44319_2025_636_MOESM6_ESM.zip › Figure3/3C/ko.jpg]

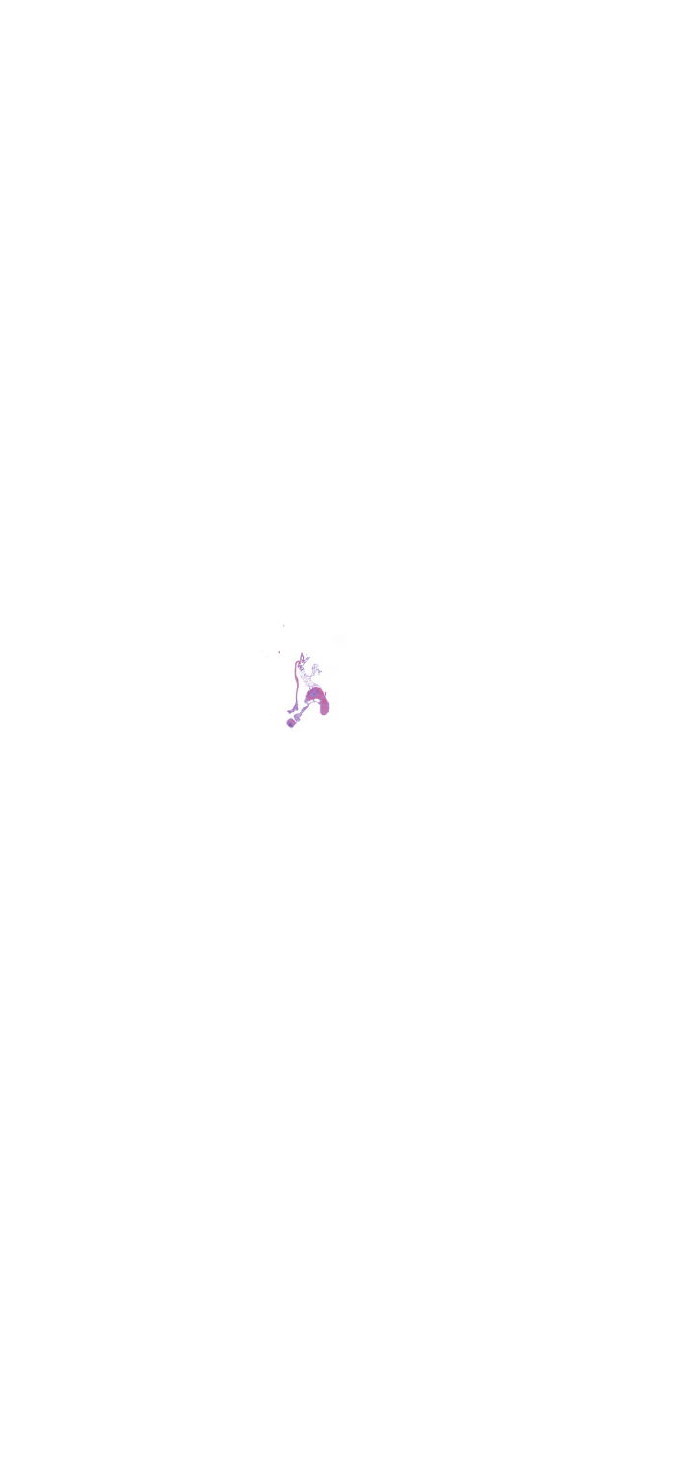

Supplement: Supplementary file 6 — Source data Fig. 3 [file 44319_2025_636_MOESM6_ESM.zip › Figure3/3C/P15 KI-G1 Masson.mrxs]

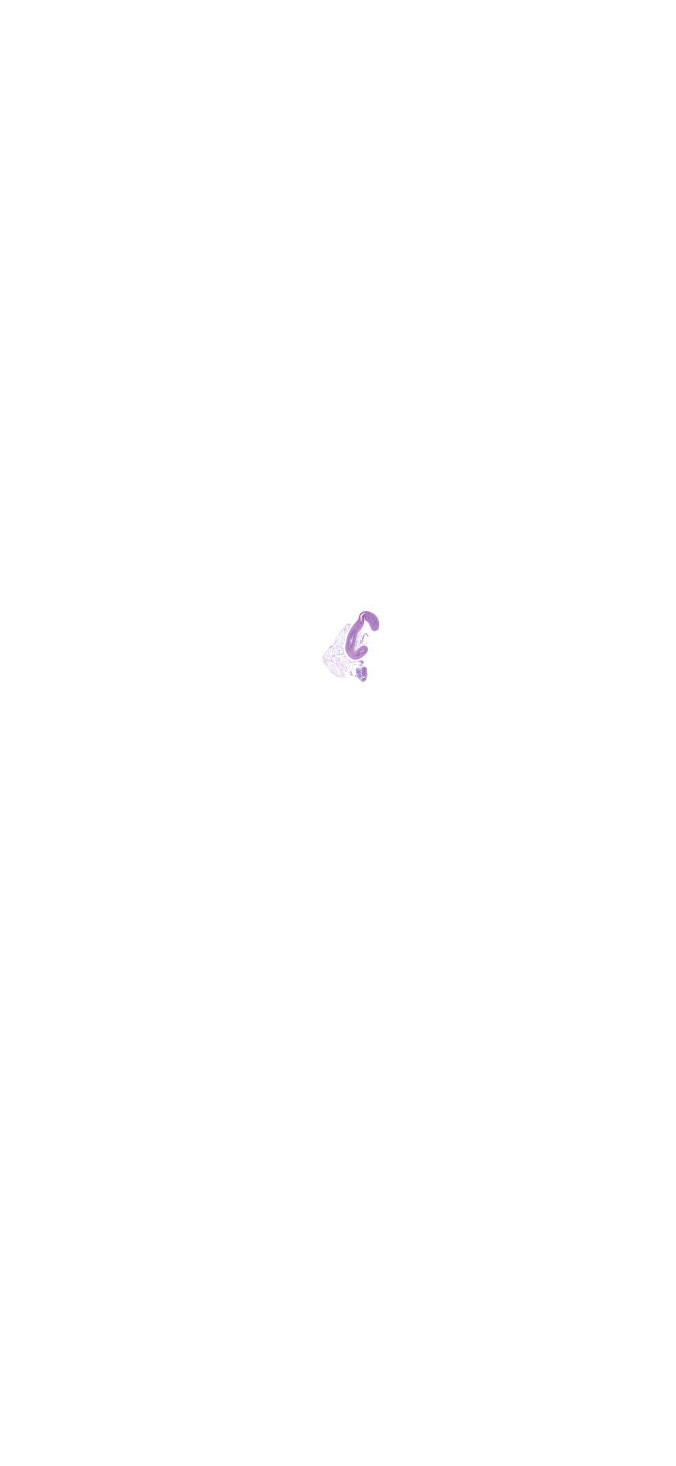

Supplement: Supplementary file 6 — Source data Fig. 3 [file 44319_2025_636_MOESM6_ESM.zip › Figure3/3C/P15 KI-G3 Masson.mrxs]

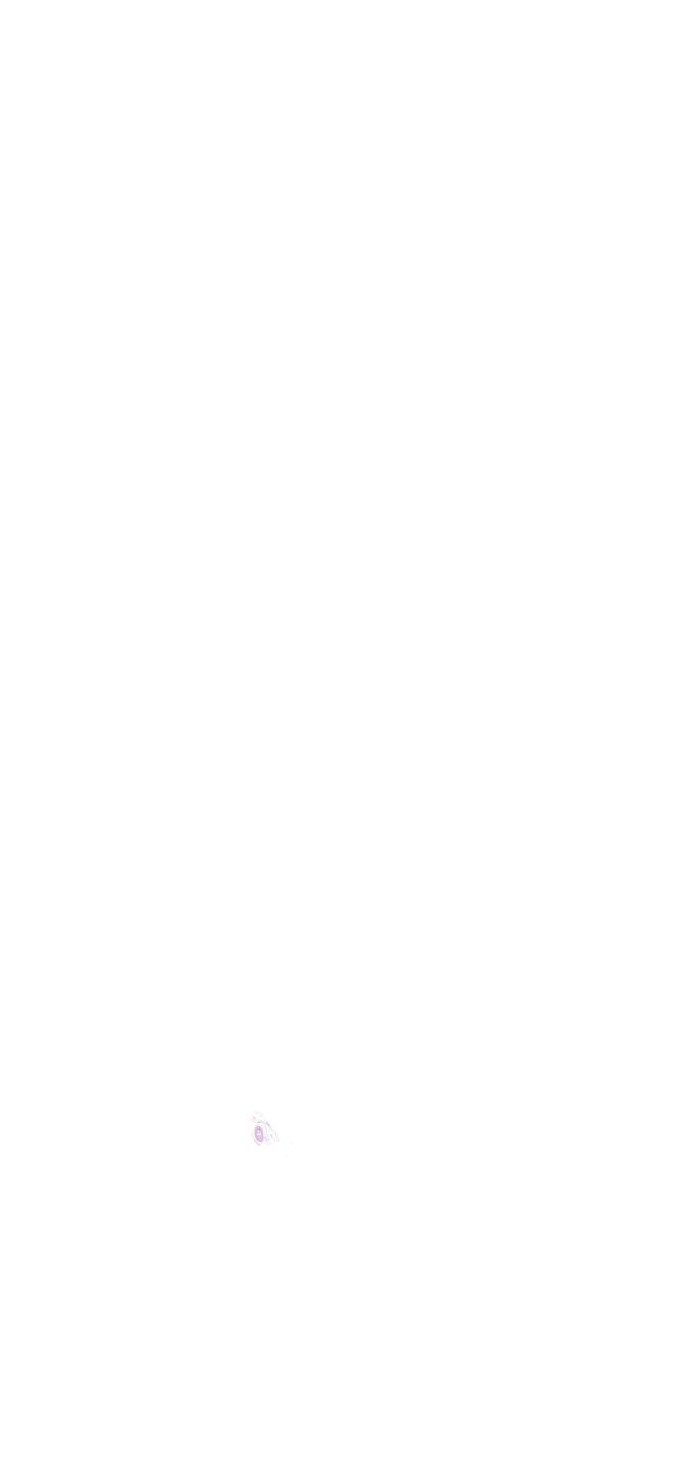

Supplement: Supplementary file 6 — Source data Fig. 3 [file 44319_2025_636_MOESM6_ESM.zip › Figure3/3C/P15 KO 69G masson.mrxs]

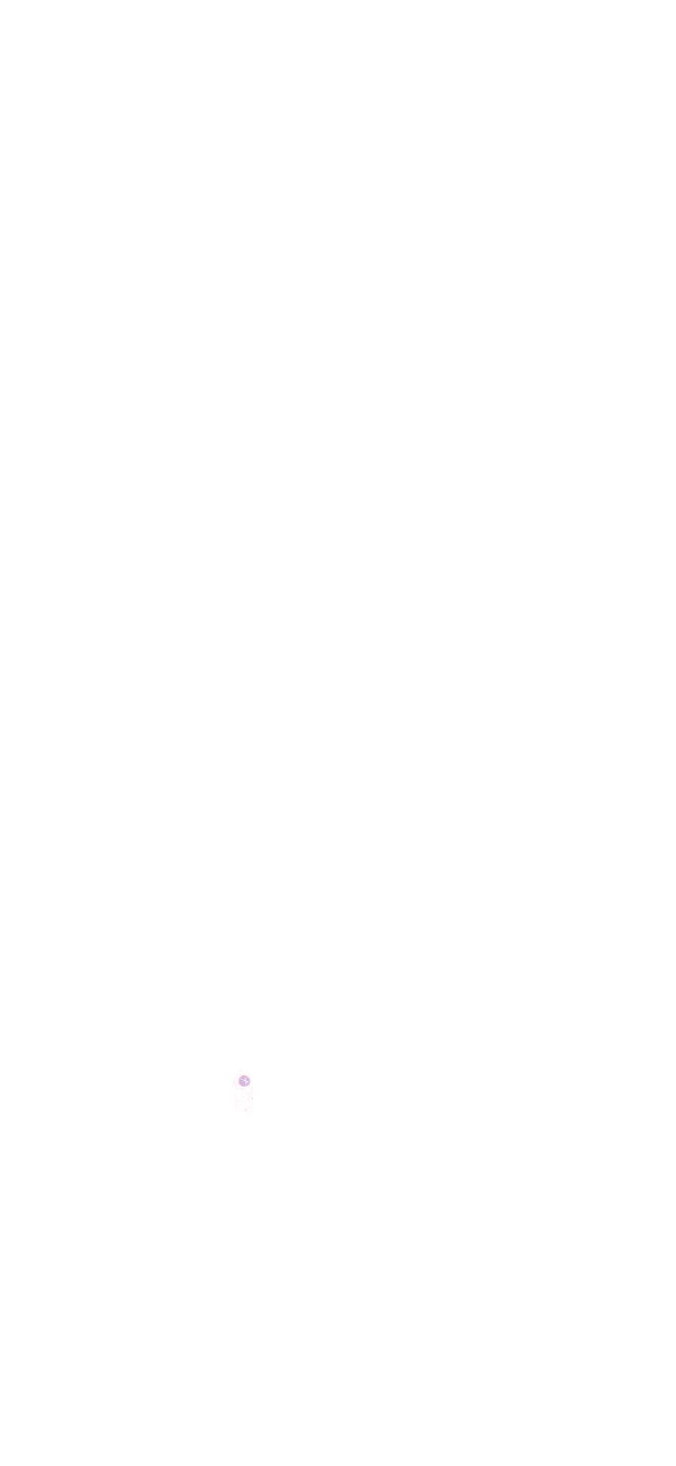

Supplement: Supplementary file 6 — Source data Fig. 3 [file 44319_2025_636_MOESM6_ESM.zip › Figure3/3C/P15 KO G masson.mrxs]

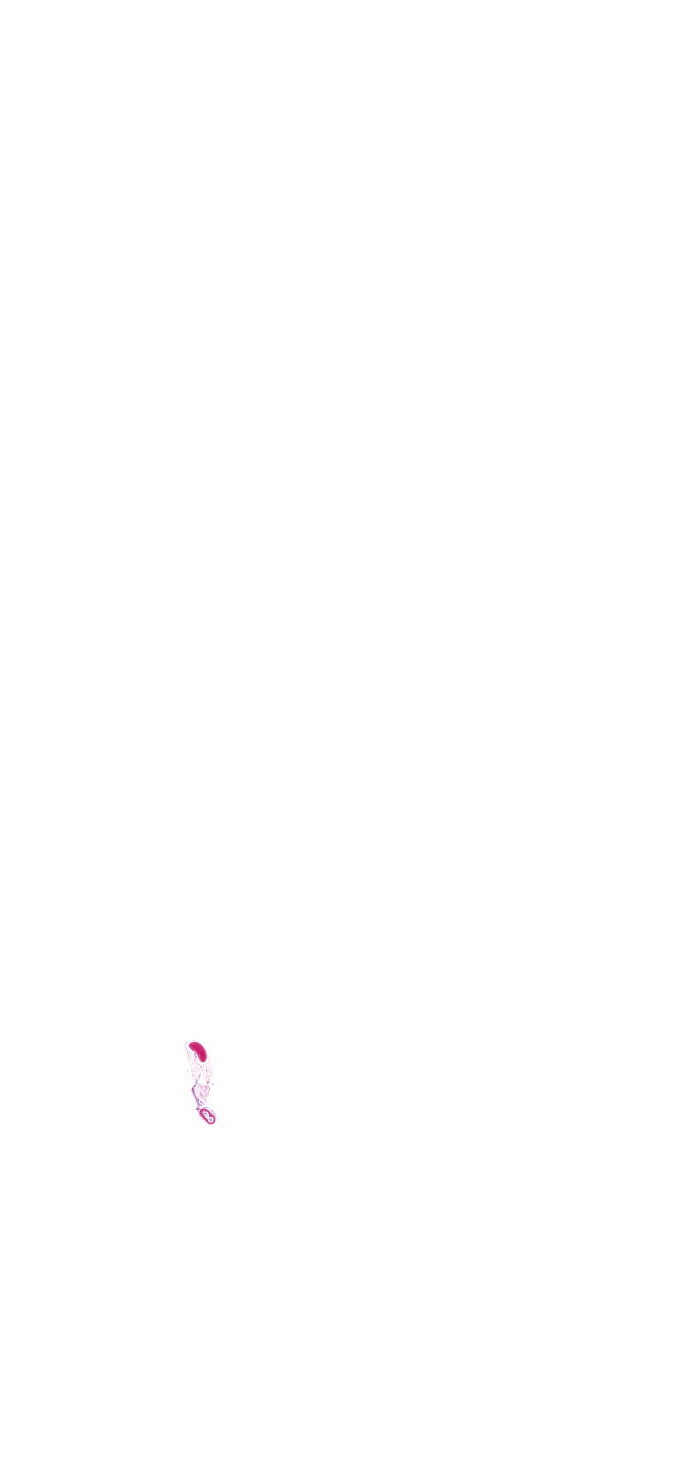

Supplement: Supplementary file 6 — Source data Fig. 3 [file 44319_2025_636_MOESM6_ESM.zip › Figure3/3C/P15 KO.mrxs]

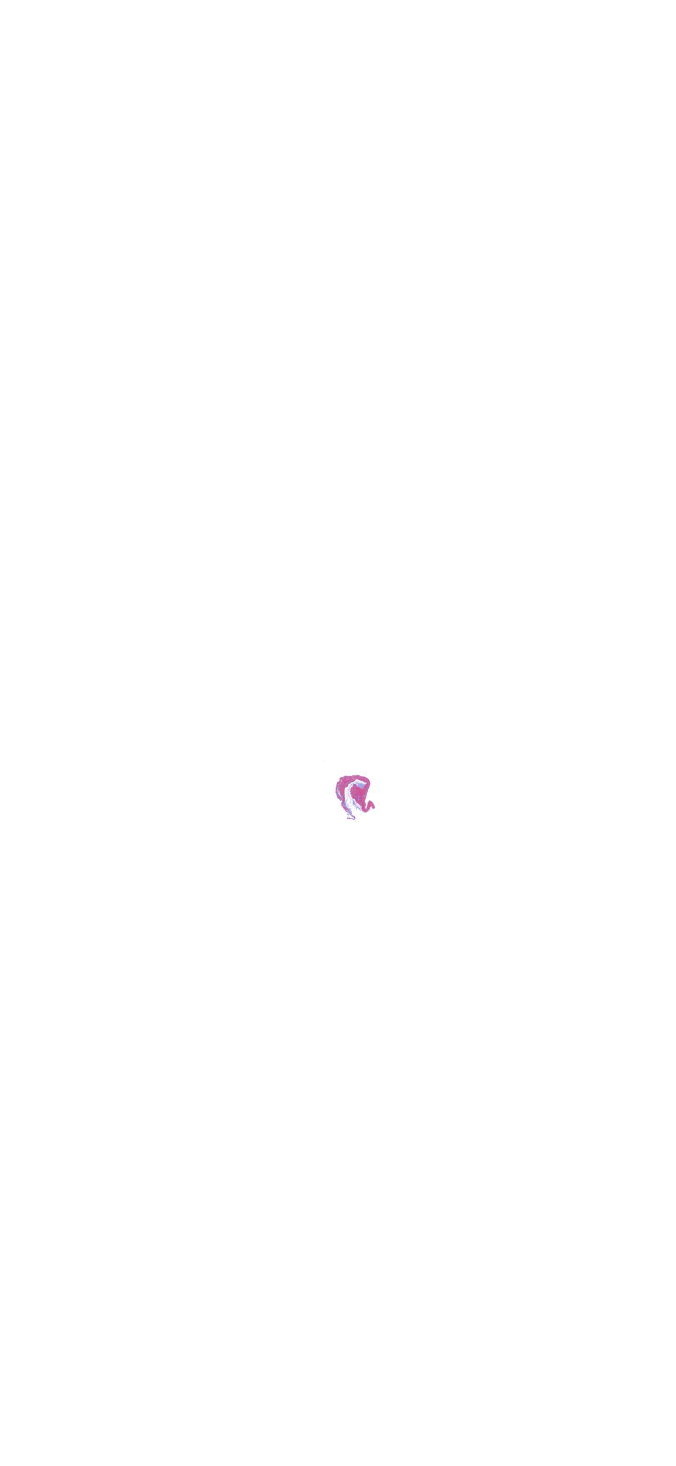

Supplement: Supplementary file 6 — Source data Fig. 3 [file 44319_2025_636_MOESM6_ESM.zip › Figure3/3C/P15 WT-G1 Masson.mrxs]

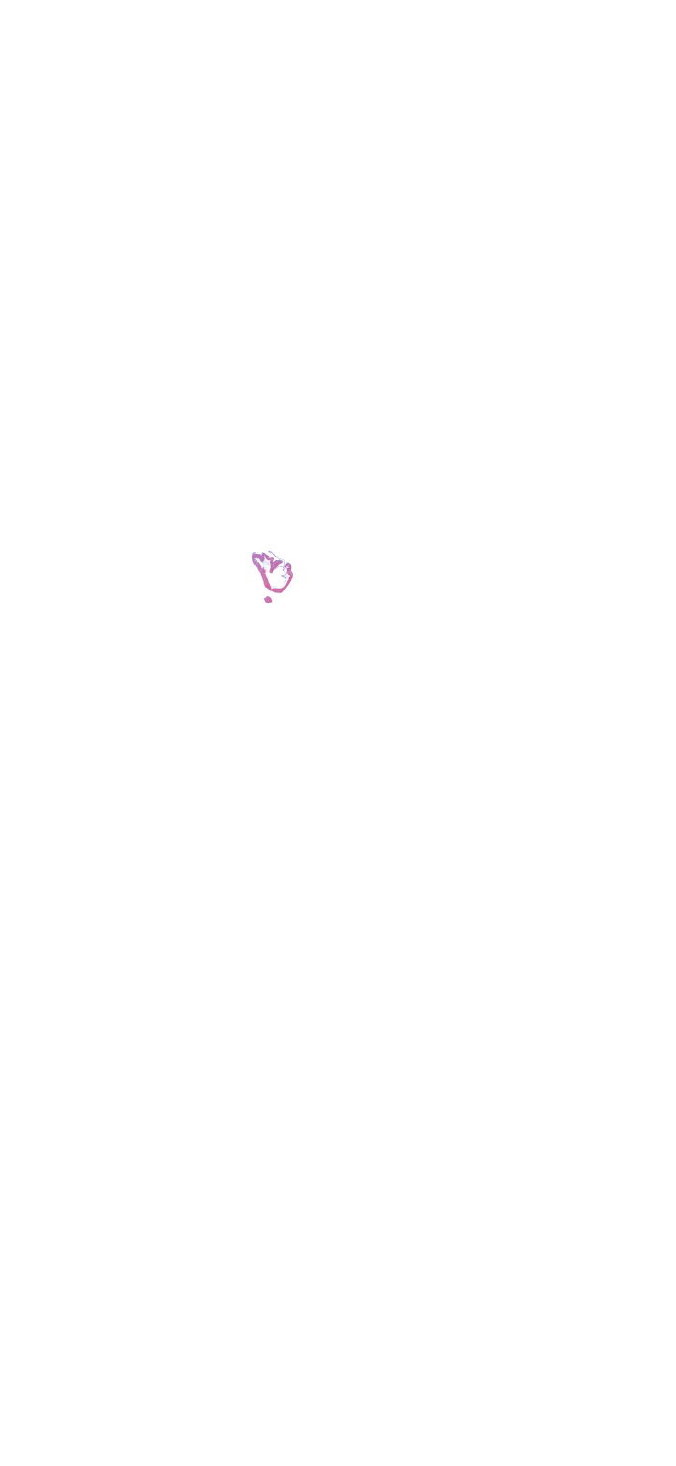

Supplement: Supplementary file 6 — Source data Fig. 3 [file 44319_2025_636_MOESM6_ESM.zip › Figure3/3C/P15 WT-G2 Masson.mrxs]

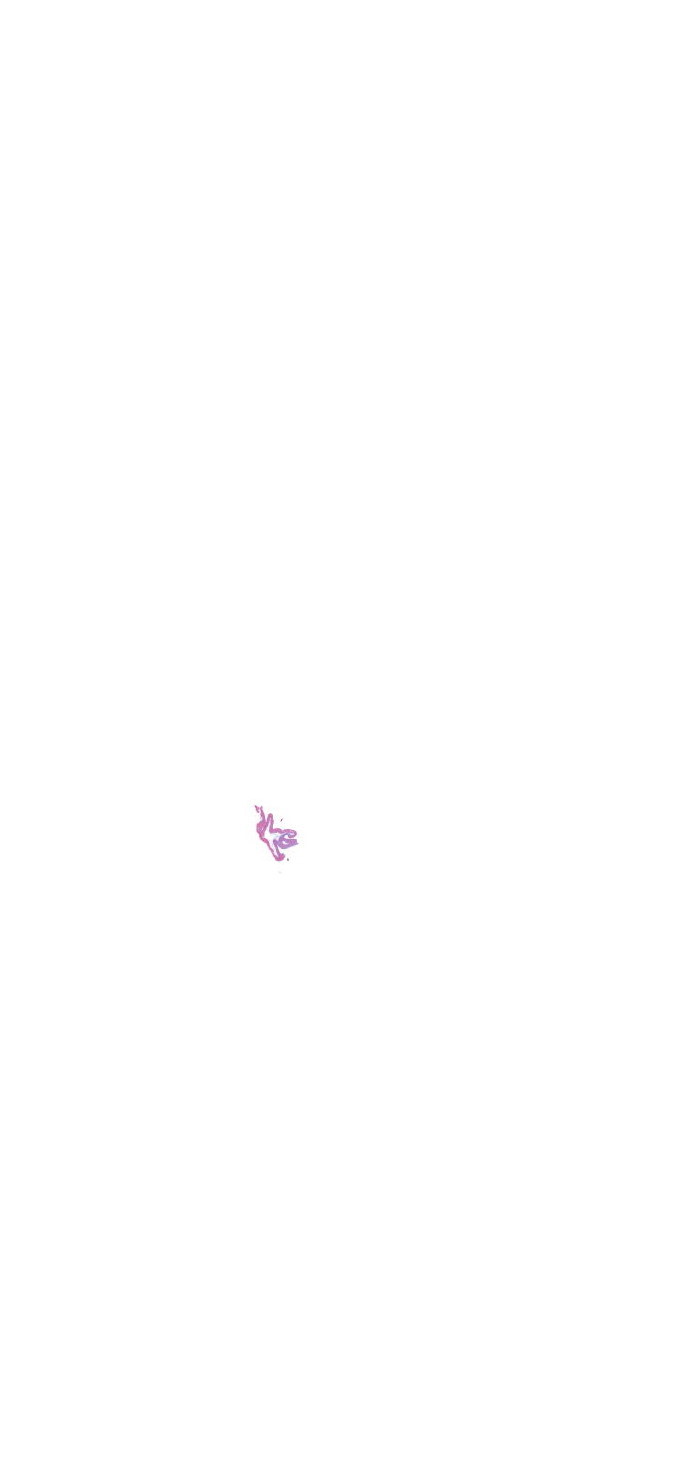

Supplement: Supplementary file 6 — Source data Fig. 3 [file 44319_2025_636_MOESM6_ESM.zip › Figure3/3C/P15 WT-G3 Masson.mrxs]

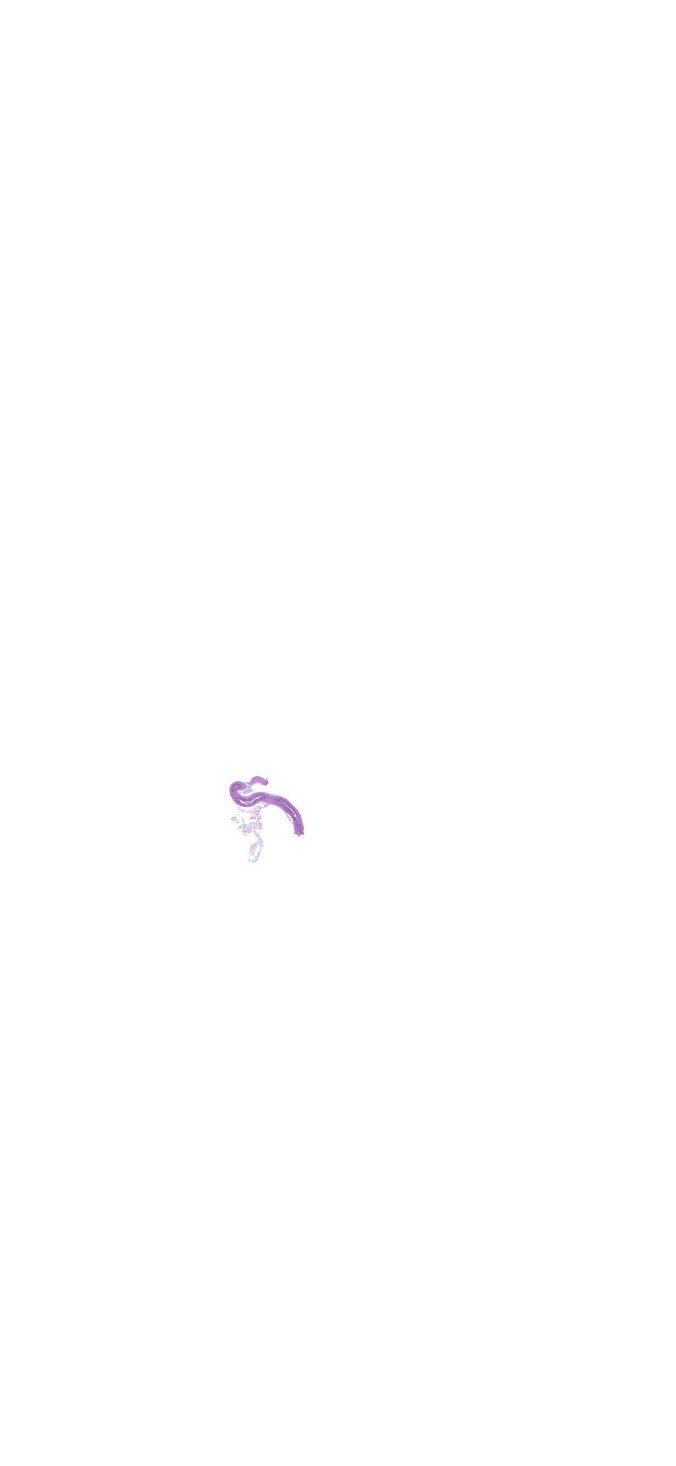

Supplement: Supplementary file 6 — Source data Fig. 3 [file 44319_2025_636_MOESM6_ESM.zip › Figure3/3C/P23 KI-G1 Masson.mrxs]

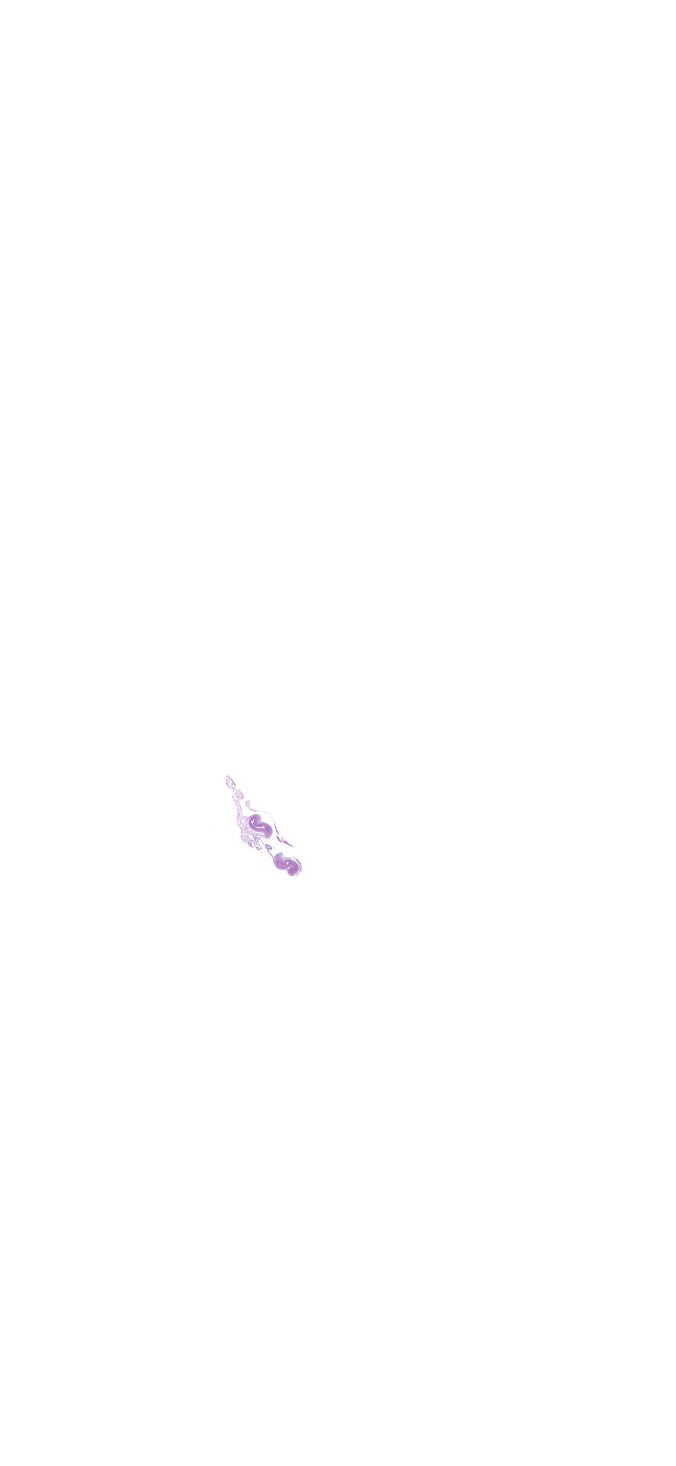

Supplement: Supplementary file 6 — Source data Fig. 3 [file 44319_2025_636_MOESM6_ESM.zip › Figure3/3C/P23 KI-G2 Masson.mrxs]

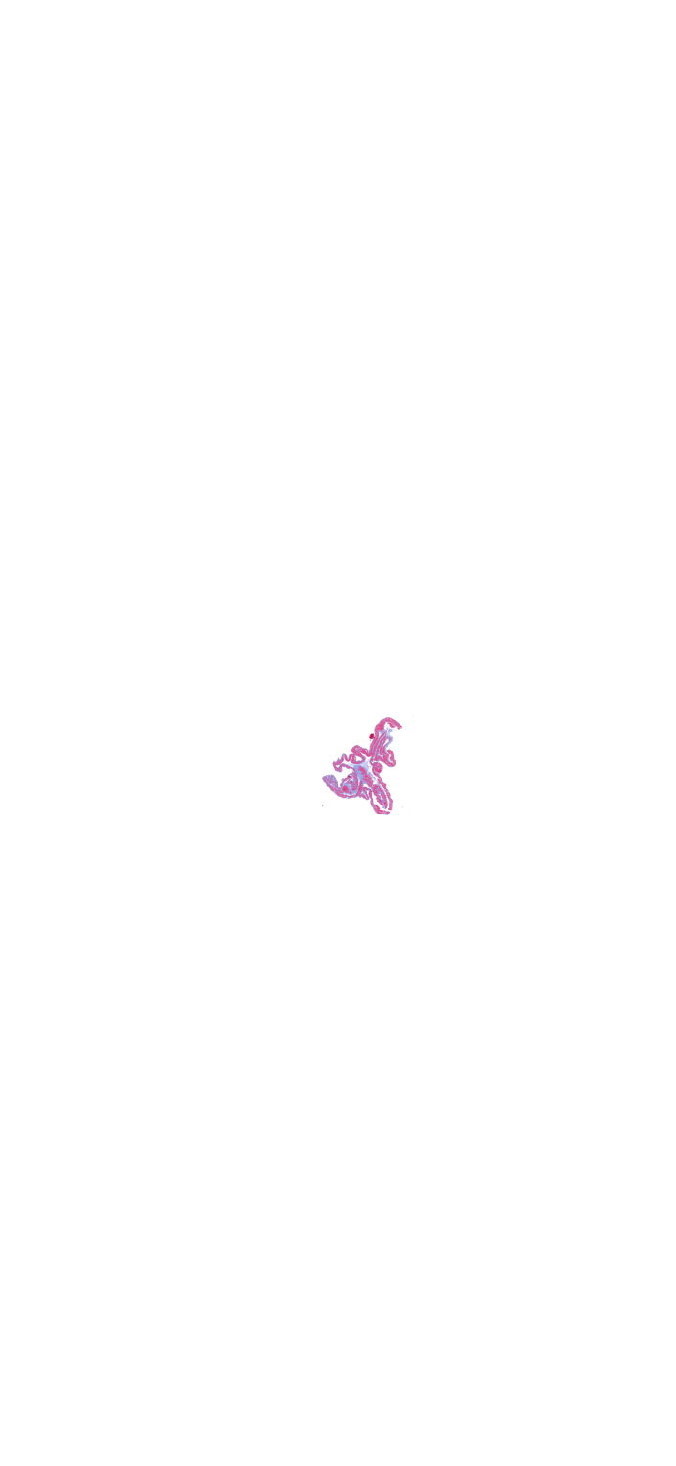

Supplement: Supplementary file 6 — Source data Fig. 3 [file 44319_2025_636_MOESM6_ESM.zip › Figure3/3C/P23 WT-G1 Masson.mrxs]

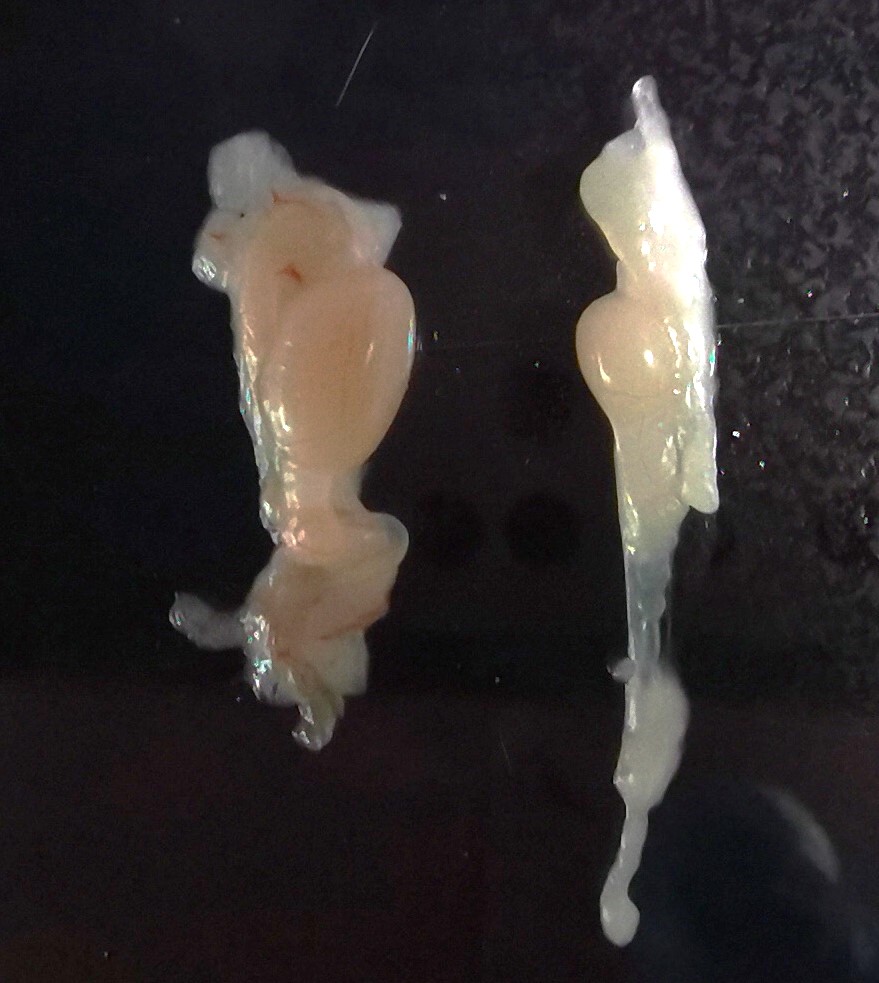

Supplement: Supplementary file 7 — Source data Fig. 5 [file 44319_2025_636_MOESM7_ESM.zip › Figure5/5A-B/WT V.S. KI.jpg]

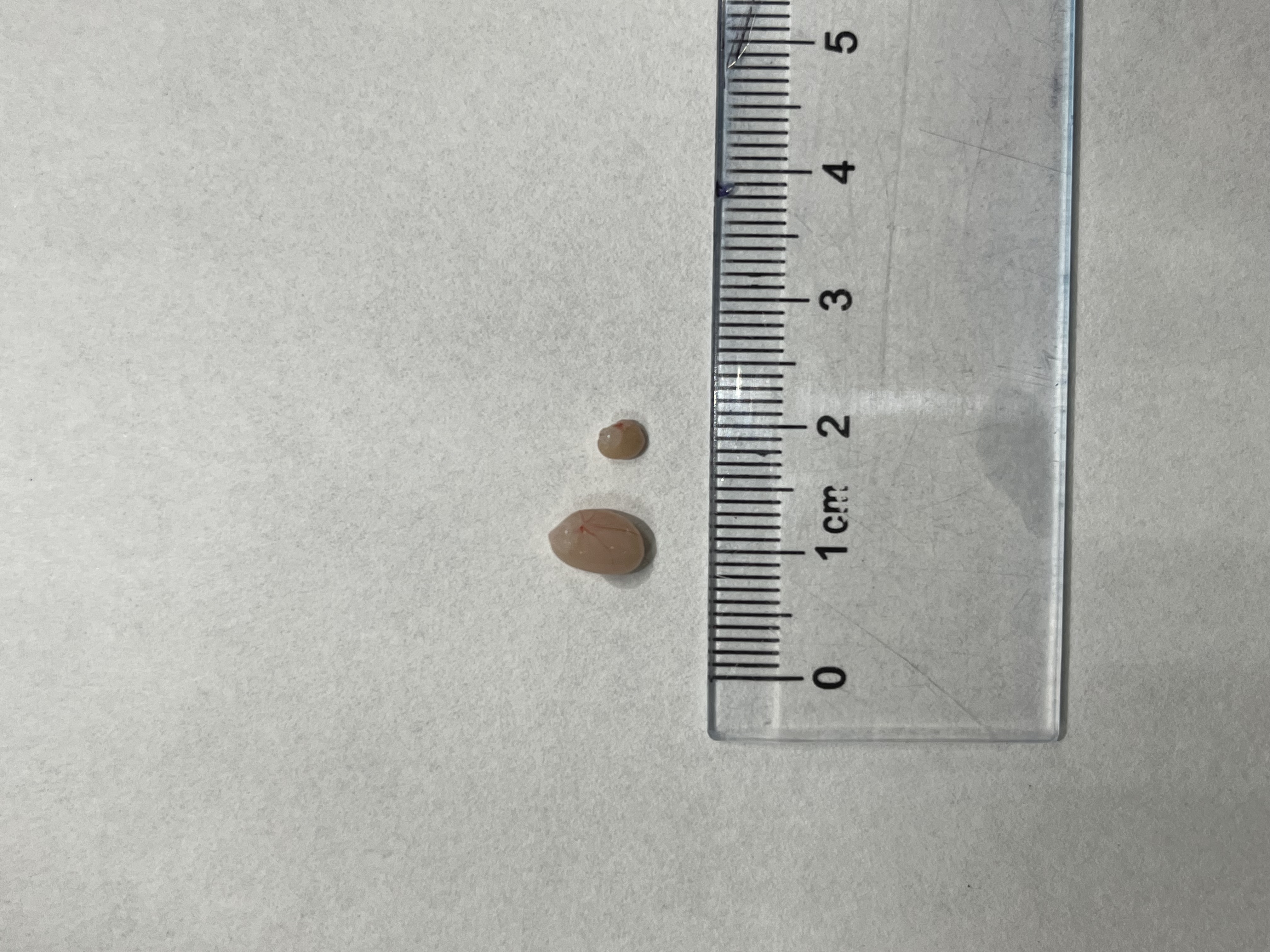

Supplement: Supplementary file 7 — Source data Fig. 5 [file 44319_2025_636_MOESM7_ESM.zip › Figure5/5A-B/睾丸对比.jpg]

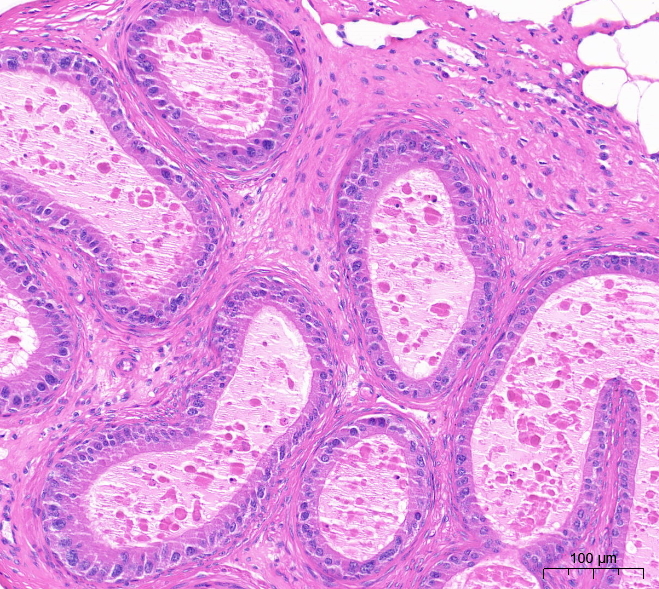

Supplement: Supplementary file 7 — Source data Fig. 5 [file 44319_2025_636_MOESM7_ESM.zip › Figure5/5C-D/1KOA63-F_10.0x.jpg]

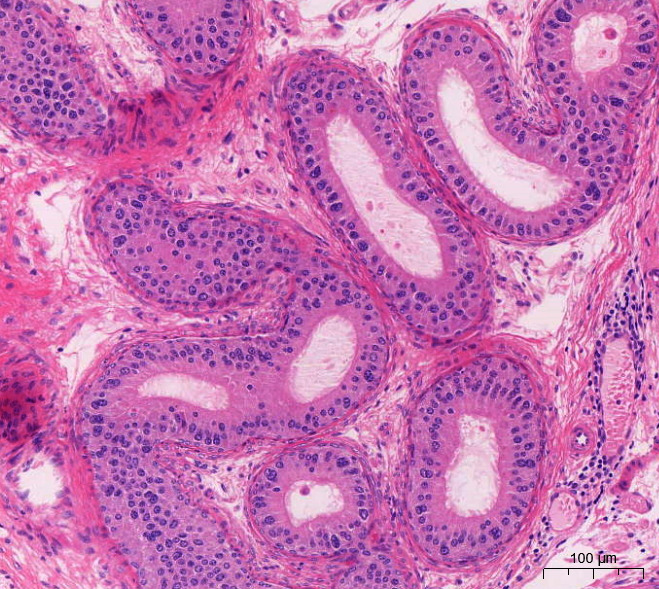

Supplement: Supplementary file 7 — Source data Fig. 5 [file 44319_2025_636_MOESM7_ESM.zip › Figure5/5C-D/KI-1 附睾尾_10.0x.jpg]

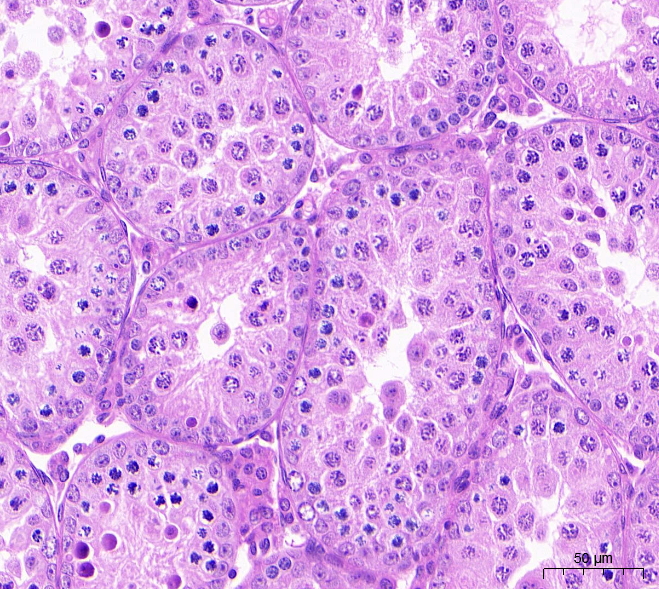

Supplement: Supplementary file 7 — Source data Fig. 5 [file 44319_2025_636_MOESM7_ESM.zip › Figure5/5C-D/P20-KI1 T HE 郑煜_20.0x.jpg]

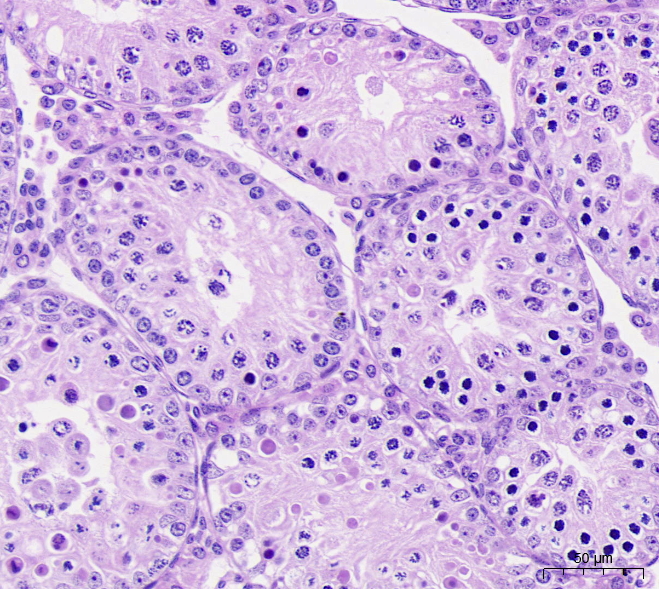

Supplement: Supplementary file 7 — Source data Fig. 5 [file 44319_2025_636_MOESM7_ESM.zip › Figure5/5C-D/P20-KO1 T HE 郑煜_20.0x.jpg]

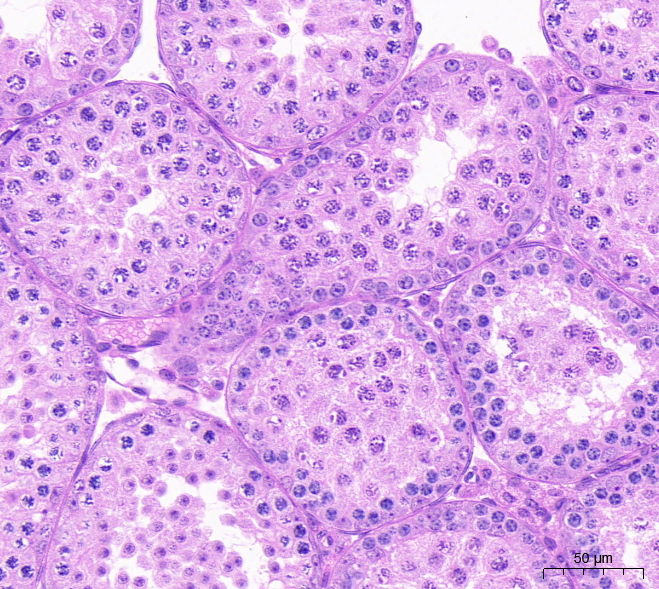

Supplement: Supplementary file 7 — Source data Fig. 5 [file 44319_2025_636_MOESM7_ESM.zip › Figure5/5C-D/P20-WT3 T HE 郑煜_20.0x.jpg]

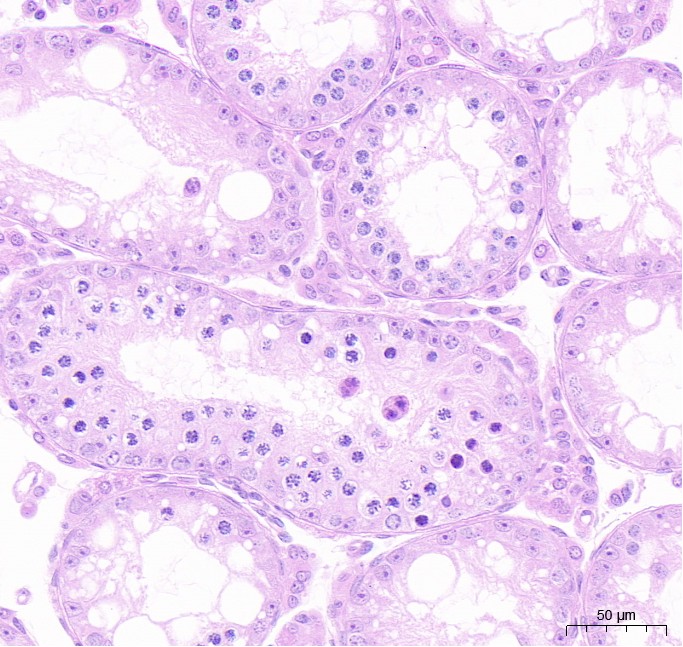

Supplement: Supplementary file 7 — Source data Fig. 5 [file 44319_2025_636_MOESM7_ESM.zip › Figure5/5C-D/P40-KI2 T HE 郑煜_20.0x.jpg]

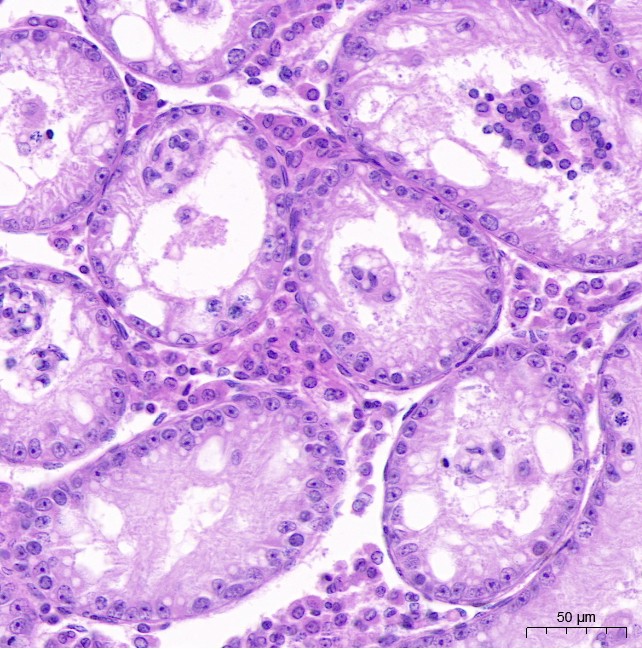

Supplement: Supplementary file 7 — Source data Fig. 5 [file 44319_2025_636_MOESM7_ESM.zip › Figure5/5C-D/P40-KO1 T HE 郑煜_20.0x.jpg]

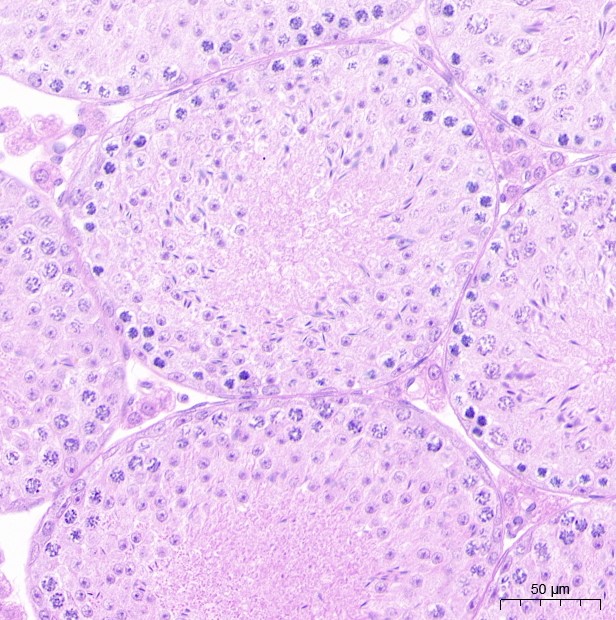

Supplement: Supplementary file 7 — Source data Fig. 5 [file 44319_2025_636_MOESM7_ESM.zip › Figure5/5C-D/P40-WT1 T HE 郑煜_20.0x.jpg]

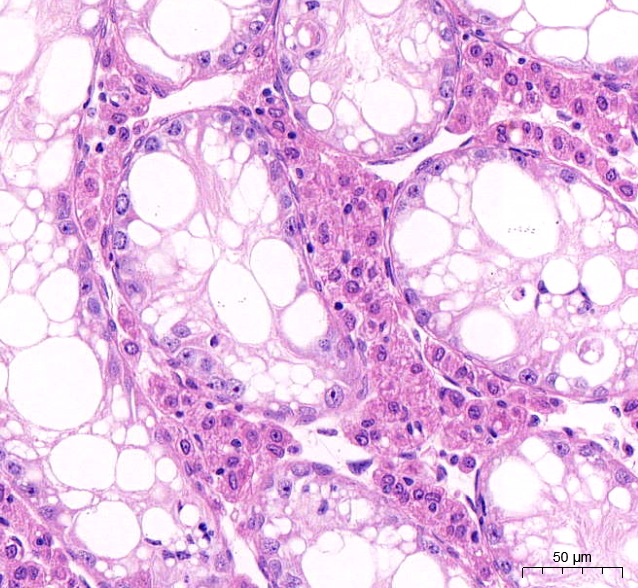

Supplement: Supplementary file 7 — Source data Fig. 5 [file 44319_2025_636_MOESM7_ESM.zip › Figure5/5C-D/P60-KI1 T HE 郑煜_20.0x.jpg]

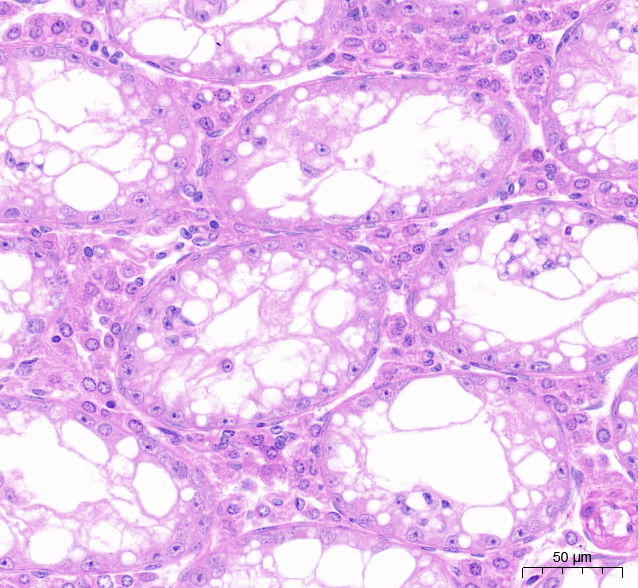

Supplement: Supplementary file 7 — Source data Fig. 5 [file 44319_2025_636_MOESM7_ESM.zip › Figure5/5C-D/P60-KO1 T HE 郑煜_20.0x.jpg]

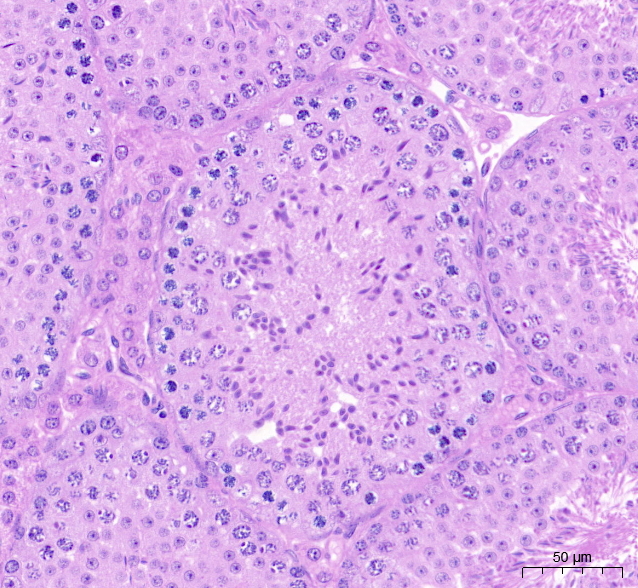

Supplement: Supplementary file 7 — Source data Fig. 5 [file 44319_2025_636_MOESM7_ESM.zip › Figure5/5C-D/P60-WT1 T HE 郑煜_20.0x.jpg]

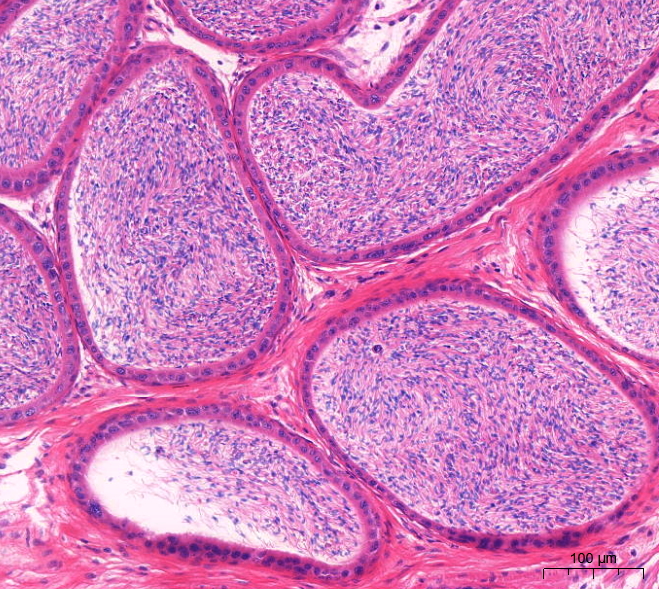

Supplement: Supplementary file 7 — Source data Fig. 5 [file 44319_2025_636_MOESM7_ESM.zip › Figure5/5C-D/WT-2 附睾尾_10.0x.jpg]
