# Supplementary material for: Evolutionary relaxation and functional change of INSL3 and RXFP2 may underlie natural cryptorchidism in mammals
Source: EMBO Rep. 2025 Nov 11;26(24):6418–36. doi: 10.1038/s44319-025-00636-w (PMC12714730; doi:10.1038/s44319-025-00636-w)
Supplement: Supplementary file 8 — Expanded View Figures [file 44319_2025_636_MOESM8_ESM.pdf]

Expanded View Figures

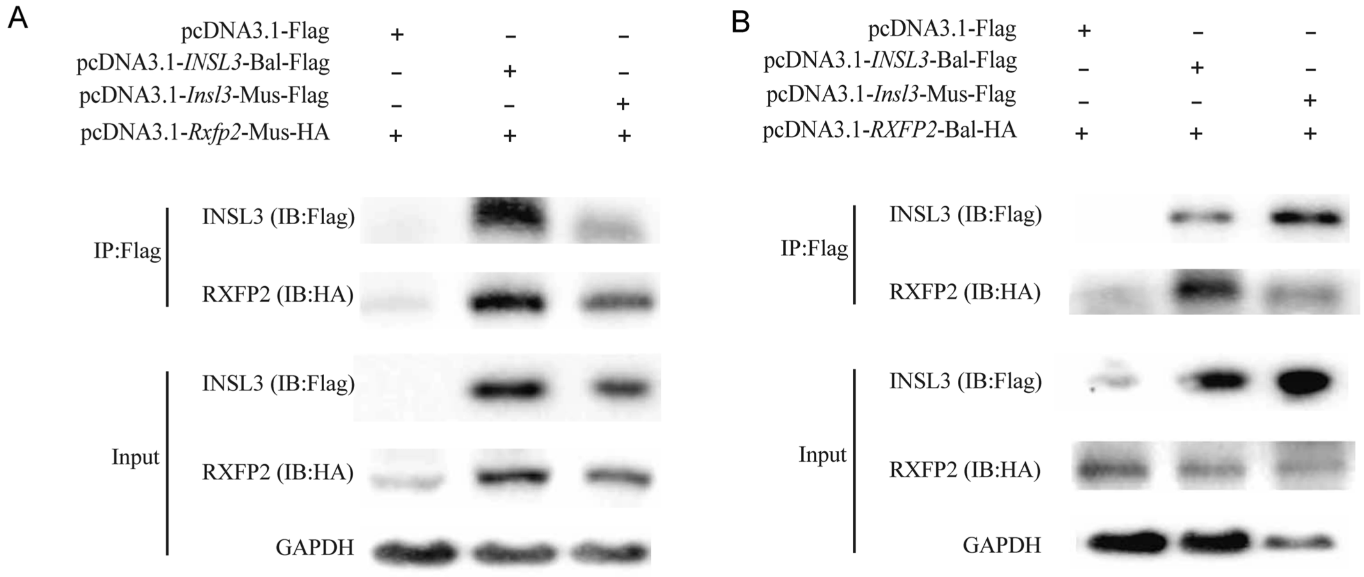

Figure EV1. Co-IP assay of INSL3 and RXFP2 binding capacity in cetaces and mice.

(**A**) Binding ability of the mouse RXFP2 receptor to mouse INSL3 and cetacean INSL3. (**B**) The binding ability of the cetacean RXFP2 receptor to mouse INSL3 and cetacean INSL3, respectively. Data were representative of $n = 3$ independent experiments.

© The Author(s)

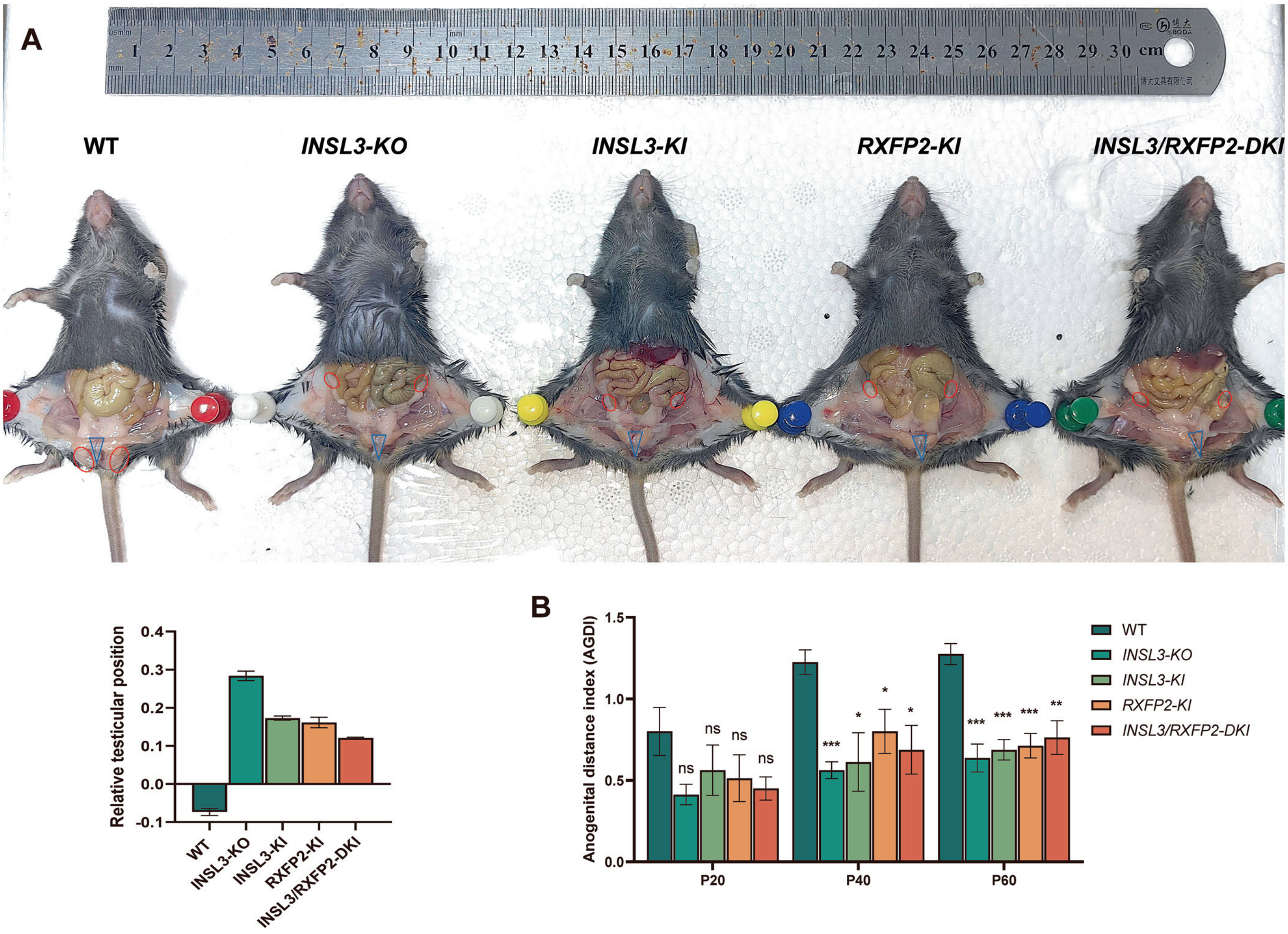

Figure EV2. Cryptorchidism is present in three cetaceanized mice.

(**A**) Anatomical map showing the in situ testicular position in mice (red circle indicates testis, blue triangle indicates penis) with quantification based on the testis-to-penis position ratio (Data were shown as mean ± s.e.m. from $n = 8$ mice, biological replicates). (**B**) AGDI values of mice at different periods (Data are shown as mean ± s.e.m. from $n = 4$ mice, biological replicates. Statistical significance was determined using ANOVA tests. Exact p values: P40 WT vs KO, $p = 0.0009$; P40 WT vs *INSL3*-KI, $p = 0.0444$; P40 WT vs *RXFP2*-KI, $p = 0.0496$; P40 WT vs DKI, $p = 0.0383$; P60 WT vs KO, $p = 0.0008$; P60 WT vs *INSL3*-KI, $p = 0.0005$; P60 WT vs *RXFP2*-KI, $p = 0.0011$; P60 WT vs DKI, $p = 0.0110$. *$p < 0.05$; **$p < 0.01$***$p < 0.005$; ns not significant).

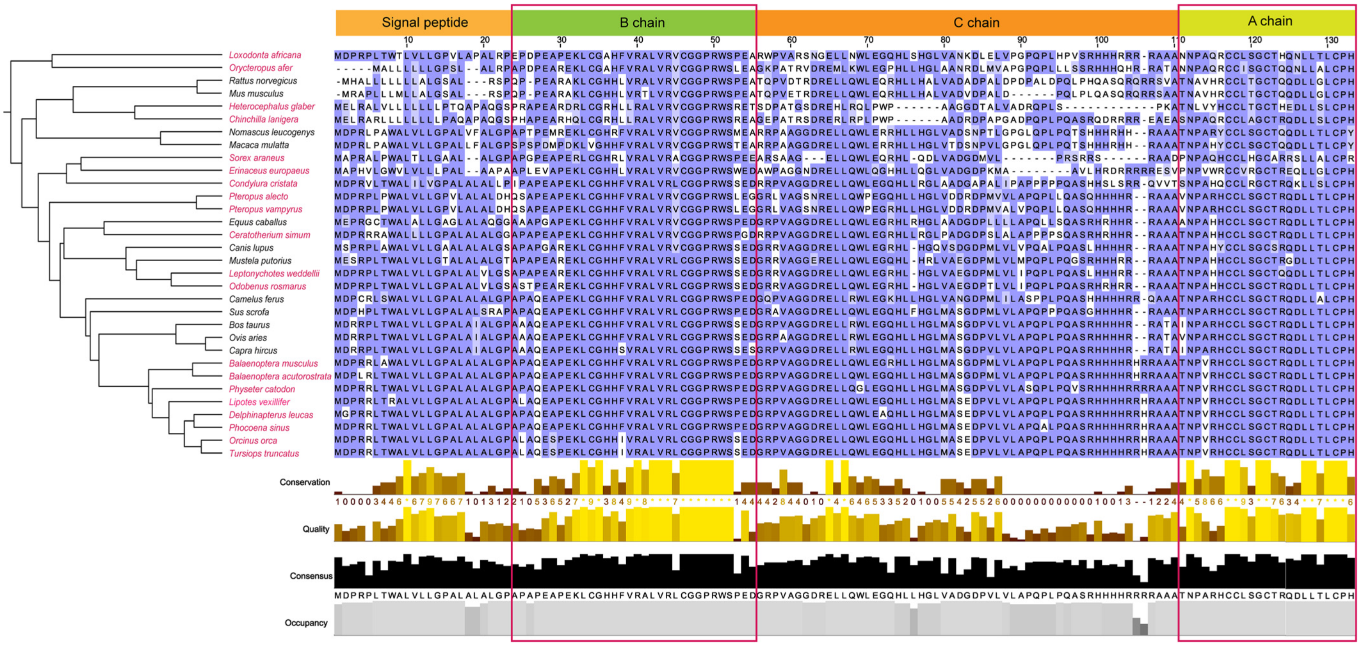

Figure EV3. The sequence of mammalian INSL3.

The red Latin name is cryptorchid mammals, and the black Latin name is scrotal mammals. The two functional domains of the mature INSL3 protein are marked in red boxes.

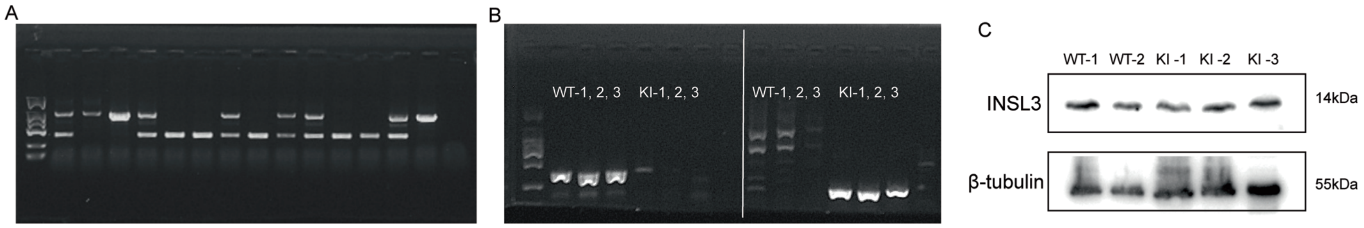

Figure EV4. The cetacean *INSL3* gene knock-in mice were successfully constructed.

(**A**) Agarose gel electrophoresis plot of genotyping. Lane 1: Maker DL2000; Lane 1, 4, 7, 9, 10, 13: heterozygote; Lane 2, 3, 14: homozygote; Lane 5, 6, 8, 11, 12: wildtype; Lane 15: blank control. (**B**) Agarose gel electrophoresis plot of RT-PCR. Lane 1: Maker DL2000; Lane 2–8: the primer was mouse *INSL3*; Lane 9–15: the primer was cetacean *INSL3*. (**C**) WB of mouse testicular proteins. Data were representative of $n = 3$ independent experiments.
